# MicroRNA 144 Impairs Insulin Signaling by Inhibiting the Expression of Insulin Receptor Substrate 1 in Type 2 Diabetes Mellitus

**DOI:** 10.1371/journal.pone.0022839

**Published:** 2011-08-01

**Authors:** Dwi Setyowati Karolina, Arunmozhiarasi Armugam, Subramaniam Tavintharan, Michael T. K. Wong, Su Chi Lim, Chee Fang Sum, Kandiah Jeyaseelan

**Affiliations:** 1 Department of Biochemistry, Yong Loo Lin School of Medicine, National University Health System, National University of Singapore, Singapore, Singapore; 2 Department of Medicine, Khoo Teck Puat Hospital, Singapore, Singapore; 3 Department of Anatomy and Developmental Biology, School of Biomedical Sciences, Faculty of Medicine, Nursing and Health Sciences, Monash University, Clayton, Victoria, Australia; University of Barcelona, Spain

## Abstract

**Background:**

Dysregulation of microRNA (miRNA) expression in various tissues and body fluids has been demonstrated to be associated with several diseases, including Type 2 Diabetes mellitus (T2D). Here, we compare miRNA expression profiles in different tissues (pancreas, liver, adipose and skeletal muscle) as well as in blood samples from T2D rat model and highlight the potential of circulating miRNAs as biomarkers of T2D. In parallel, we have examined the expression profiles of miRNAs in blood samples from Impaired Fasting Glucose (IFG) and T2D male patients.

**Methodology/Principal Findings:**

Employing miRNA microarray and stem-loop real-time RT-PCR, we identify four novel miRNAs, miR-144, miR-146a, miR-150 and miR-182 in addition to four previously reported diabetes-related miRNAs, miR-192, miR-29a, miR-30d and miR-320a, as potential signature miRNAs that distinguished IFG and T2D. Of these microRNAs, miR-144 that promotes erythropoiesis has been found to be highly up-regulated. Increased circulating level of miR-144 has been found to correlate with down-regulation of its predicted target, insulin receptor substrate 1 (*IRS1*) at both mRNA and protein levels. We could also experimentally demonstrate that *IRS1* is indeed the target of miR-144.

**Conclusion:**

We demonstrate that peripheral blood microRNAs can be developed as unique biomarkers that are reflective and predictive of metabolic health and disorder. We have also identified signature miRNAs which could possibly explain the pathogenesis of T2D and the significance of miR-144 in insulin signaling.

## Introduction

The discovery of microRNAs (miRNAs) by Ambros and co-workers in 1993 has introduced another level of intricacy in the regulation of the genome [1]. While miRNAs mainly inhibits translation by binding to the 3′ untranslated region (3′UTR) of their target mRNA [2], they are also known to induce gene activation [3]–[6]. Since their discovery, miRNAs have become the focus of intensive research and indentified as key regulators in governing physiological and pathological processes [7]–[10]. Besides their recognized intracellular regulatory function, growing evidence suggests that miRNAs show stable extracellular existence. These circulating miRNAs are detected in body fluids including saliva, urine and blood [11], [12]. Recently, there have been a growing number of blood-based miRNA profiling studies which reported perturbations in the expression of blood miRNAs and introduced the concept that circulating miRNAs hold much potential as fingerprints of several diseases [13]–[18] including Type 2 diabetes (T2D) [19], [20]. A recent study by Laterza *et al* [21] established the general principle that biomarkers of disease are secreted into the systemic circulation upon tissue injury. The team then demonstrated how circulating miRNAs may serve as potential indicators of what is happening at tissue level. An independent investigation by Kosaka *et al* [22] has also reported how these circulatory miRNAs are released through secretory machinery and then transferred to the recipients where they can resume their functions. One possible secretory machinery is the exosomes, microvesicles that are present in biological fluids such as urine, saliva and blood [23]–[26]. Within these exosomes are cellular gene products including miRNAs, mRNAs, and proteins that can be transferred to recipient cells to carry out specific molecular functions [27]. Such interactions allow exosomes mediate cell-to-cell communication by facilitating the exchange of molecular components. Based on these reports, we can now perform blood-based miRNA profiling to search for fingerprints of diseases. To date, many researchers have proven that a non-invasive approach of circulating blood-based miRNAs identification of biomarkers is extremely valuable and useful in diseases including diabetes [13]–[19].

T2D is one of the most prevalent metabolic disorders, and it is estimated to affect more than 400 million by 2030, of which more than half will be living in Asia. T2D is postulated to arise from an interplay of genetic and environmental/epigenetic factors which leads to a decline in insulin action, followed by a chronic pancreatic beta-cell dysfunction. When reduction in insulin function (insulin resistance) occurs, euglycemia is maintained by increased insulin secretion (hyperinsulinemia). Progressive deterioration in insulin sensitivity and reduction in pancreatic insulin secretion, create a state of relative insulin deficiency, resulting in hyperglycemia, presenting as impaired fasting glucose (IFG) at early stage or T2D at advanced stage [28]. Despite progress made in the study of mechanisms underlying IFG and T2D [29], the understanding of these metabolic disorders at molecular level remains to be elucidated.

Among more than 10000 miRNAs identified in 115 species so far [Sanger miRNA database release 14.0 http://www.mirbase.org/], only a handful have been linked to glucose metabolism and metabolic disorders [30]. Among them, miR-375 that is abundantly expressed in pancreatic and beta cells, negatively regulates glucose-stimulated insulin secretion via myotrophin (*Mtpn*) inhibition [31], [32]. Other miRNAs predicted to target *Mtpn* include miR-124a and let-7b [33]. Up-regulation of miR-29a/b observed in diabetic animal models has also been shown to induce insulin resistance in cell culture [34]. Altered expression of miR-192 and miR-377 has also been reported in diabetic kidney glomeruli and diabetic nephropathy respectively [35], [36]. Most miRNA studies relating to T2D were carried out in either animal or cell culture models [37]–[40] and a few in heterogeneous groups of patients with T2D, who may have had differences in terms of clinical phenotype and medications used for treatment [19], [41], [42]. In this report, we compare the miRNA expression in rat model of T2D with carefully phenotyped newly diagnosed pre-diabetic (IFG) and overt diabetic (T2D) patients who are not on any medication. IFG patients are included in the study so as to gain further understanding in the transition of miRNA expression from pre-diabetes stage to overt T2D. In addition, using animal models we have explored the relevance of circulating blood miRNAs to that of different tissues in T2D pathogenesis. Among the eight miRNAs identified, miR-144 remained highly up-regulated in T2D in both cases. In this study, we have also provided evidence that miR-144 directly inhibits *IRS1*, a key molecule in insulin signaling.

## Results

### Induction of T2D in male Wistar rats

**Figure 1:**
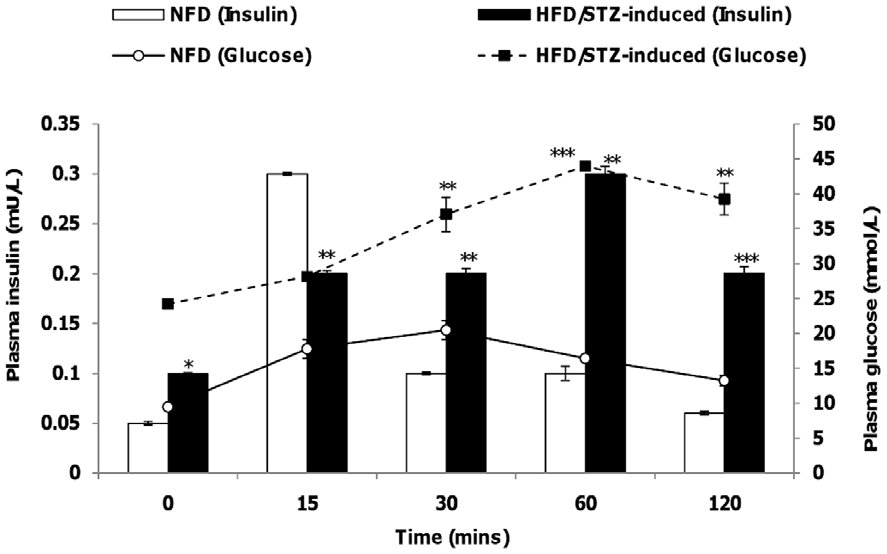
Oral glucose tolerance test (OGTT). Serum glucose and insulin concentrations of both NFD and HFD groups upon OGTT. Data presented as mean ± SEM with n = 6 for each group. Statistically significant differences are tested at p<0.05 significance. ^*^p<0.05, **p<0.01, ***p<0.001.

Animals were fed with either normal diet (NFD) or high fat diet (HFD) for two weeks before a low dose STZ (40 mg/kg) was administered intra-peritoneally to the HFD group. Combination of high-fat diet and low dose of STZ injection has been widely used to induce T2D in rats and proven to effectively induce T2D whereby the pattern of disease onset and development appears to be closely analogous to that in the human syndrome [43]–[45]. This approach is preferred to administrating single high dose of STZ which results in Type 1 Diabetes characteristics [46]–[48]. The effects of the treatment on body weight, glucose, insulin, cholesterol and triglycerides concentrations are listed on Table 1. These results show that the HFD rats weighed approximately 20 g more than the NFD rats. Although the serum cholesterol level of the two groups were similar, HFD rats showed significantly higher serum levels of triglycerides, glucose and insulin. Figure 1 shows the serum glucose and insulin levels after oral glucose tolerance test (OGTT). NFD rats showed a peak increase in their serum glucose concentration at 15 min (∼20mmol/L) after glucose intake but was reduced quickly to normal level within 2 hours (∼12mmol/L). On the contrary, serum glucose concentration of HFD rats increased rapidly and remained at high level even at 2 hours after glucose intake (∼40mmol/L). The insulin response was almost 3-times higher in the HFD rats as compared with their NFD controls. Despite the higher insulin concentration, glucose level was approximately 3-times higher in HFD rats indicating a state of hyperglycemia and insulin resistance.

**Table 1:** Effect of STZ administration on HFD rats as compared to NFD healthy controls.

	Before Treatment		After treatment (2 weeks later)	
	Control (NFD)	HFD/STZ induced T2D	Control (NFD)	HFD/STZ induced T2D
**Weight**	204.67±4.55	211.67±3.78	336.33±5.58	359.33±8.03 *
**Cholesterol (mmol/L)**	2.48±0.11	2.47±0.10	1.99±0.14	2.32±0.10
**Triglycerides (mmol/L)**	1.72±1.05	2.06±0.15	1.22±0.18	3.18±0.17 *
**HDL-C (mmol/L)**	0.94±0.05	0.83±0.06	0.44±0.05	0.55±0.03
**LDL-C (mmol/L)**	0.94±0.09	0.86±0.10	0.87±0.16	0.63±0.15
**Glucose (mmol/L)**	8.27±0.74	8.93±0.26	11.53±1.23	30.08±3.45 *
**Insulin (mU/L)**	0.11±0.01	0.10±0.00	0.13±0.00	0.33±0.02 *

NFD, normal diet healthy controls; HFD, high fat diet; STZ, streptozotocin; LDL-C, low-density lipoprotein cholesterol; HDL-C; high-density lipoprotein cholesterol. Values are mean ± SD with n = 6 for each group. Statistically significant differences are tested at p<0.05 significance, denoted by^*^_._

### miRNA profiling in blood and tissue samples of T2D-induced rats

**Figure 2:**
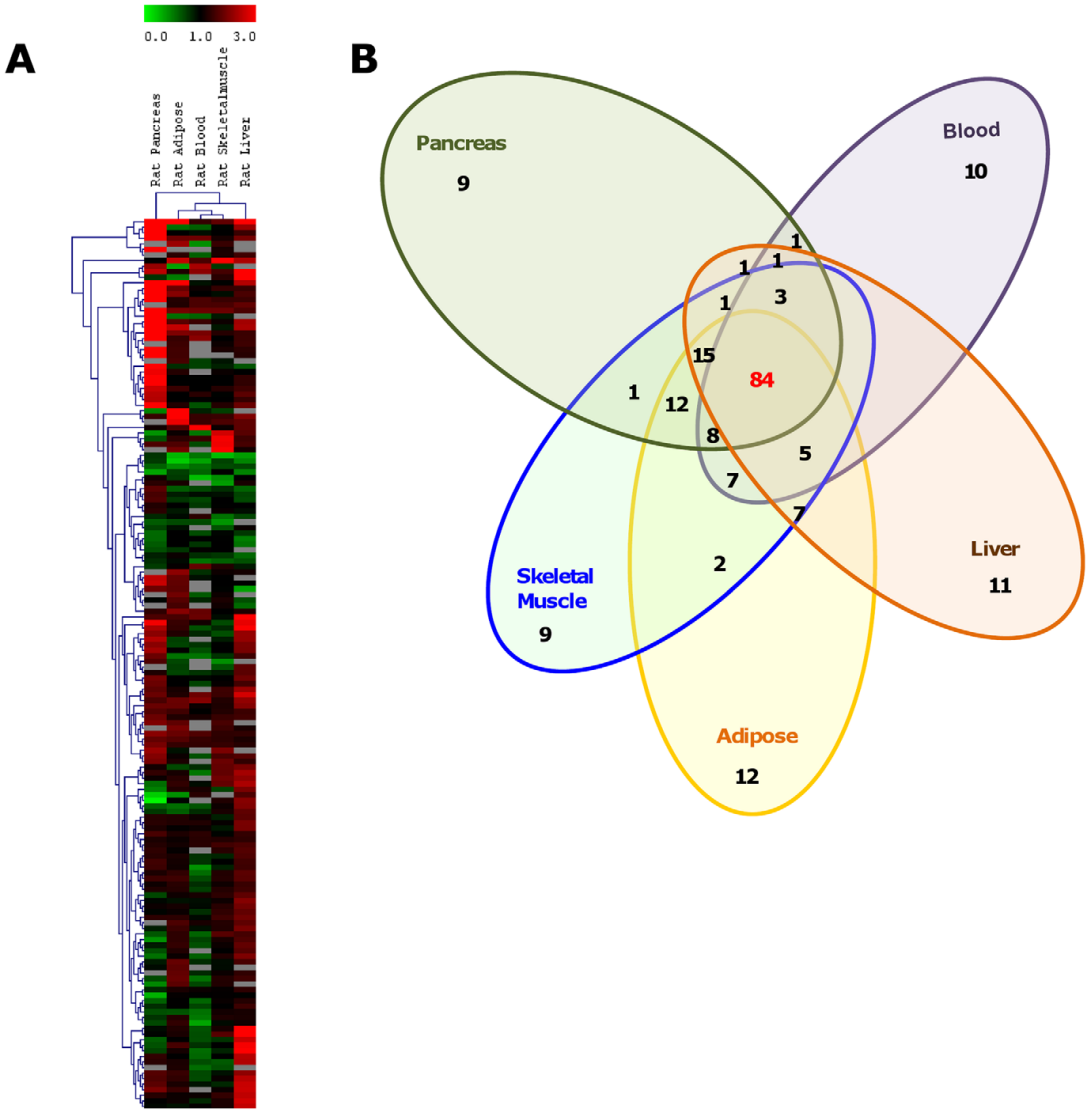
Clustering of miRNA profiles of blood and tissues of HFD rats. ***A.*** **Hierarchical clustering plot (heatmap) of miRNA distribution in blood and tissues of HFD rats (compared to controls).** Only miRNAs conserved in humans with background subtracted mean signal intensities above 300 are included. Expression of blood miRNAs are clustered in the centre closely to the peripheral tissues. Data are expressed as fold change of HFD versus NFD control. Green: down-regulation; Red: up-regulation; Grey: not detected. ***B.*** **Venn diagram showing the number of miRNAs with significant changes detected in blood and the different tissues (Diabetic vs controls).** 84 miRNAs have been detected in all five sources.

To understand the relevance of circulating blood miRNAs expression to those selected tissues, we performed miRNA microarray to compare the miRNA expression in blood and four diabetes-related tissues namely, pancreas, liver, adipose and skeletal muscles. Only miRNAs that are conserved in humans have been taken into account. miRNA microarray data was evaluated after filtering off those miRNAs that showed signal intensities of 300 and below. Approximately 200 miRNAs were detected among the five sources of miRNAs. Differential miRNA expression in the blood and the other four tissues between T2D model (HFD group) compared to that of control (NFD group) are presented in a hierarchical clustering plot (heatmap) generated using the TIGR multiple experimental viewer software (Fig. 2A, Table S1, GEO Accession No. GSE26167; SuperSeries GSE26168). Figure 2A shows that blood miRNAs are clustered closely to that of the insulin-targeted tissues, first being the skeletal muscles followed by adipose and then liver. Pancreas which secretes insulin, has more miRNAs being up-regulated and was clustered away on the left of the heatmap. To focus on the more important miRNAs, we have selected those miRNAs that showed significant changes as compared to control (p<0.05). The number of miRNAs that showed significant changes in any of the five sources is displayed in a Venn diagram (Fig. 2B). On average, each source has approximately 130 miRNAs that showed significant changes in HFD compared to NFD. Among these, 84 miRNAs are detected in all five sources. The heatmap clustered blood miRNAs in the centre of the peripheral tissues and the Venn diagram showed that most miRNAs detected in the peripheral tissues were similarly detected in the blood. Moreover, a recent blood-based miRNA profiling study led by Heneghan *et al* [13] compared the different circulating medium (serum, plasma, or whole blood) to find out which best represented miRNA levels. The team reported that higher yields of miRNAs were obtained from whole blood, compared with either serum or plasma and that there was no significant difference between the mean white cell, hemoglobin or hematocrit levels among the samples used (controls versus cases). This finding further strengthened the notion that the whole blood is the preferred source for studying circulating miRNAs. From the pool of miRNAs that showed significant changes, we focused our investigation on those that showed at least ±1.5 fold change (Table S2). We identified miR-146a, miR-182 and miR-30d to be among the highly down-regulated miRNAs across all five sources. miR-146a expression was the lowest in the adipose (−4.62±0.036) while miR-30d which was postulated to participate in insulin gene transcription [49], showed the lowest expression in pancreas (−2.81±0.004). miR-182 was also down-regulated in all five tissues with the lowest expression observed in skeletal muscle

(−4.23±0.548). In all five sources, miR-144, miR-150, miR-192, miR-29a and miR-320a were found to be highly up-regulated. Association of miR-192 [35], miR-29a [34] and miR-320 [50], [51] in T2D has been reported in previous studies and our study also shows similar results. miR-144 showed the highest up-regulation among other miRNAs in three tissues namely pancreas (7.94±0.171), adipose (4.34±0.178) and liver (4.26±0.174). Similarly, up-regulation of miR-150 was observed to be highest in the adipose (3.21±0.197) followed by liver (2.07±0.388). Using these results, we then extended our study to the peripheral blood samples of individual with IFG or T2D.

### Characteristics of IFG and T2D male patients

Based on the miRNA profiles from animal tissues of T2D model, we observed that expression of circulating blood miRNAs is generally reflective of that in pancreas and insulin-target tissues. IFG patients were included in the study to gain further understanding in the transition of miRNA expression from pre-diabetes stage to T2D. A total 50 males were selected from 158 individuals recruited for health screening. The selected individuals had a mean age of 40.5±9.1 years, fasting glucose 5.1±1.3 mmol/L, SBP 129.0±15.1 mmHg and DBP 82.2±9.6 mmHg. From these, a group of twenty-one age-matched individuals were selected as the first batch (Batch A) and categorized into Control, CTL (n = 7), Impaired Fasting Glucose, IFG (n = 6) and Type 2 Diabetes, T2D (n = 8) for the study. Their BMI was ≤27 [52]. Table 2 describes the demography of these individuals. After investigating the distinctive miRNA expression profiles from this first batch of IFG and T2D individuals, a second batch of individuals (Table 2- Batch B) with CTL (n = 8), IFG (n = 8) and T2D (n = 13) was selected to validate the expression of the potential signature miRNAs in the two metabolic conditions.

**Table 2:** Clinical characteristics of study participants.

	CTL		IFG		T2D	
Batch	A (n = 7)	B (n = 8)	A (n = 6)	B (n = 8)	A (n = 8)	B (n = 13)
**Age (years)**	46.3±7.5	43.3±5.7	49.0±7.6	48.0±10.4	46.7±3.4	41.0±12.1
**BMI**	22.4±2.3	24.4±3.1	24.1±2.7	30.0±3.9	24.5±1.1	28.0±4.9
**Fasting glucose ** ***≤6.0mmol/L***	4.7±0.7^*^	4.7±0.5^*^	6.4±0.1^*^	6.4±0.1^*^	8.8±1.9^*^	10.6±4.3^*^
**TC ** ***≤5.0 mmol/L***	4.6±0.6	4.5±0.8	4.7±0.4	5.2±0.8	4.8±0.2	5.8±1.1
**LDL ** ***≤3.4mmol/L***	2.9±0.4	2.6±0.5	2.9±0.7	3.3±0.8	3.0±0.3	3.4±0.8
**SBP ** ***≤140 mmHg***	121.3±11.9	117.7±7.1	125.8±15.1	125.0±10.5	125±11.2	129±18.6
**DBP ** ***≤90 mmHg***	75.3±9.2	71.3±7.8	80.4±10.0	82.0±9.6	79.7±6.4	78.0±10.6

All participants in batch A were normotensive with desirable cholesterol levels and BMI ≤27. Participants in batch B showed minor differences in values for total cholesterol and BMI.

Normal values for each measurement are indicated in italics. CTL, healthy controls with fasting glucose <6.1 mmol/l; IFG, impaired fasting glucose with fasting glucose above 6.0mmol/l but less than 7.0 mmol/l; T2D, type 2 diabetes with fasting glucose greater or equal to 7.0mmol/l; TC, total cholesterol; LDL-C, low-density lipoprotein cholesterol; HDL-C; high-density lipoprotein cholesterol; SBP, systolic blood pressure; DBP, diastolic blood pressure. Values are mean ± SD. Statistically significant differences are tested at p<0.05 significance, denoted by^*^_._

### miRNA profiles of T2D rat model and T2D patients

**Figure 3:**
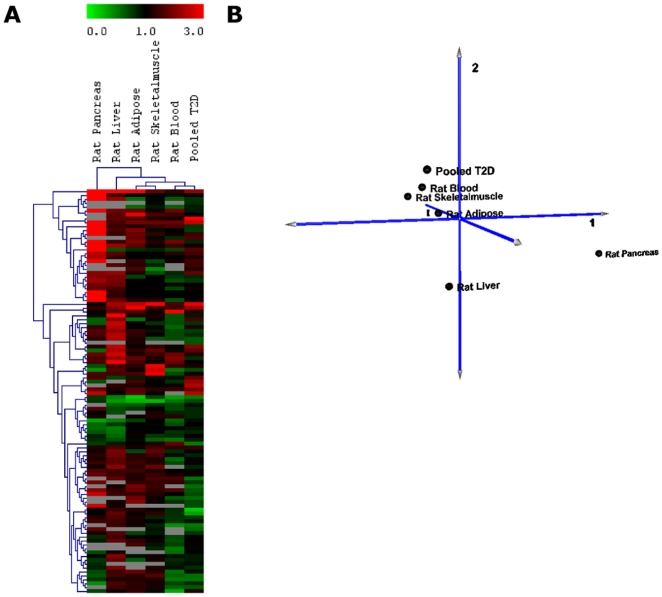
Clustering of miRNA profiles of HFD rats and pooled T2D patients. ***A.*** **Hierarchical clustering plot (heatmap) of miRNA distribution in HFD rats and pooled blood from T2D patients.** Blood samples from HFD rats and pooled T2D patients are clustered closely to one another. Data are expressed as fold change of HFD versus NFD control. Green: down-regulation; Red: up-regulation; Grey: not detected. ***B.*** **Principal component analysis (PCA) showed similar clustering as heatmap.**

Figure 3A displays the hierarchical clustering of the miRNA profiles in T2D rat model and pooled T2D patients. We have shown previously that the rat blood miRNA profile (Fig. 2A) is reflective of those in the tissues under study and from Figure 3A, it is apparent that the blood miRNA profiles of the pooled T2D patients and the T2D rat model are clustered in one branch, with the rat pancreas miRNA profile segregating independent to the others. The PCA plot of the miRNA profiles of these six groups also shows their relationships to each other (Fig. 3B).

### miRNA profiling in patients (Batch A)

**Figure 4:**
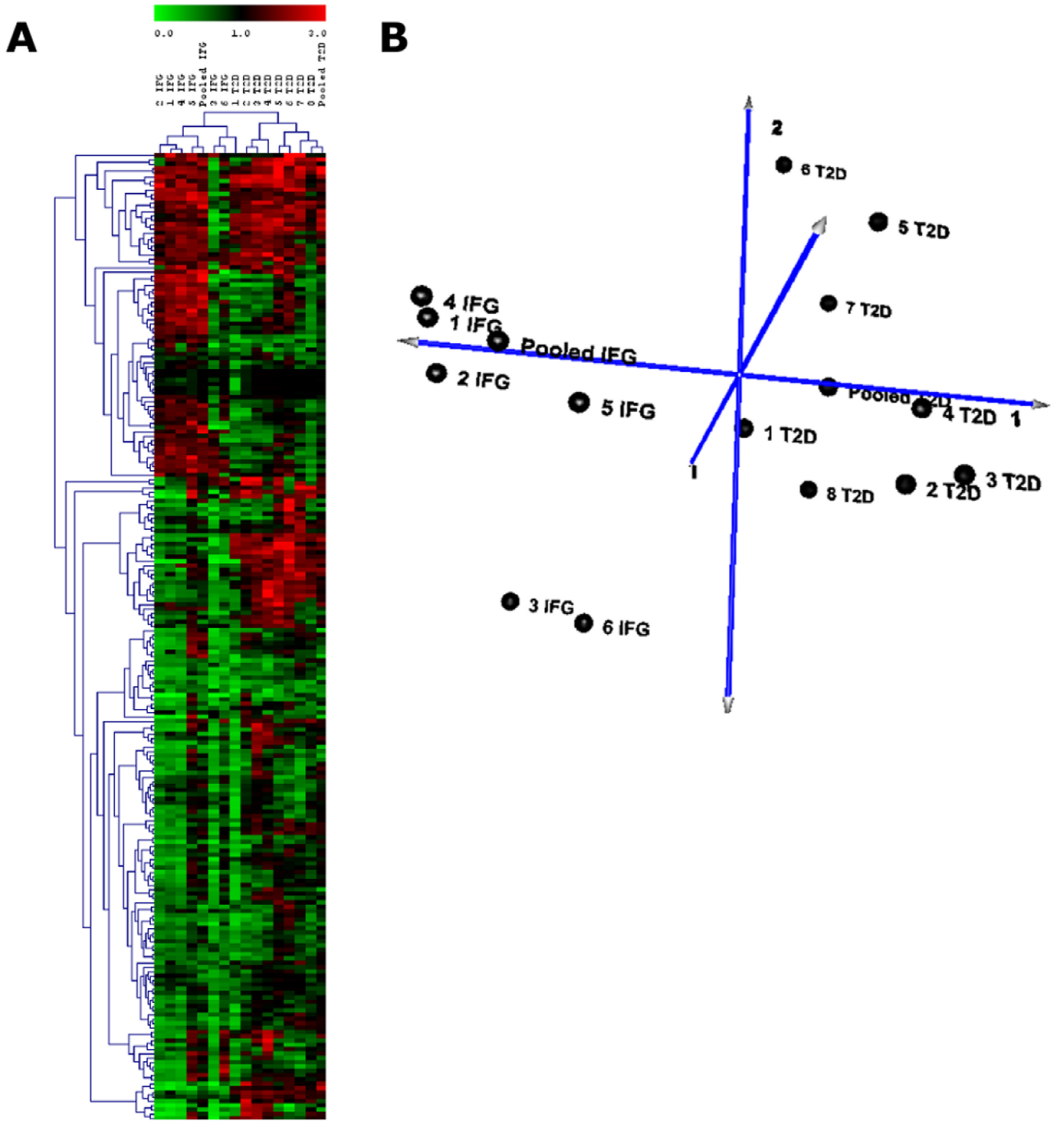
Clustering of miRNA profiles of IFG and T2D patients (Batch A). ***A.*** **Hierarchical clustering plot (heatmap) of miRNA distribution in IFG and T2D patients.** Only miRNAs with background subtracted mean signal intensities above 300 are included. Data are expressed as fold change of case versus healthy control. Green: down-regulation; Red: up-regulation; Grey: not detected. ***B.*** **Principal component analysis (PCA) plot showed a distinguished distribution of IFG and T2D patients based on miRNA expression.** Batch A patients consists of six IFG patients (labeled as 1 IFG to 6 IFG) and eight T2D patients (labeled as 1 T2D to 8 T2D). CTL, healthy controls; IFG, impaired fasting glucose; T2D, type 2 diabetes.

Next, we evaluated the total blood miRNA profile of both IFG and T2D patients to observe how the miRNA expression change as the disease develops. About 200 miRNAs with background subtracted mean signal intensities >300 were detected in each group. The expression patterns of blood miRNAs in IFG and T2D compared to that of CTL are presented in a hierarchical clustering plot (heatmap) generated using the TIGR multiple experimental viewer software (Fig. 4A). This indicated a closer relationship in miRNA expression among patients within the same group (i.e. IFG patients were clustered on one branch and T2D patients on the other). PCA (Fig. 4B) also showed a distinctive distribution of individuals with IFG and T2D. Compared to that of CTL, approximately 50 miRNAs were up-regulated while about 70 miRNAs were down-regulated in the IFG group (Fig. 4A, Table S3). The T2D group showed approximately 70 miRNAs to be up-regulated and about 100 miRNAs to be down-regulated (Fig 4A, Table S3, GEO Accession No. GSE21321; SuperSeries GSE26168).

As the two groups showed differential miRNA profiles, we next explored for significant miRNA changes within each group. This was achieved with the next level of filtering in which we discarded miRNA changes that were not significant (p>0.05) and not replicated in at least 50% of the subjects in any of the two categories (IFG or T2D as compared to CTL). Approximately 120 miRNAs passed through this second stage of filtering (Table S4). Pearson Correlation scatter plots on these miRNAs indicated a weak correlation between IFG and CTL subjects with *R* value of 0.78 (Table S5A) and a much more weaker correlation (*R* = 0.68) between T2D and CTL (Table S5B). We compared the expressions of these miRNAs between IFG and T2D group (Table S4) and identified miRNAs that could distinguish the two metabolic conditions. The eight miRNAs (miR-144, miR-146a, miR-150, mR-182, miR-192, miR-29a, miR-30d and miR-320) which were previously identified in the rat study showed similar expression in the patients' blood miRNAs. These eight miRNAs were then selected for further investigation.

### miRNA stem-loop real-time RT-PCR and mRNA targets

**Figure 5:**
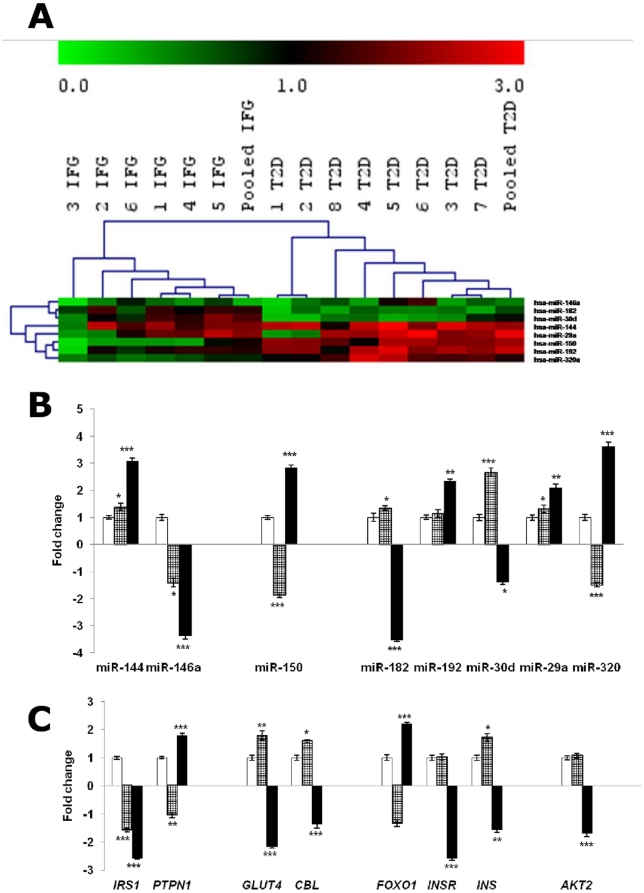
Identification of ‘signature miRNAs’ expressed in IFG and T2D (Batch A). ***A.*** **Heatmap of the potential ‘signature miRNAs’ expressed in IFG and T2D.** ***B.*** **Quantitative stem-loop RT-PCR of eight selected miRNAs in IFG (checkered bars) and T2D (black bars) against CTL (white bars) and** ***C.*** **Real-time PCR of respective predicted mRNA targets in IFG (checkered bars) and T2D (black bars) against CTL (white bars).** Data presented as mean ± SEM of both individual and pooled samples of each category. Fold change below one are expressed as negative values. Validation is done in triplicates. Statistically significant differences are tested at p<0.05 significance. ^*^p<0.05, **p<0.01, ***p<0.001. Batch A patients consists of six IFG patients (labeled as 1 IFG to 6 IFG) and eight T2D patients (labeled as 1 T2D to 8 T2D). CTL, healthy controls; IFG, impaired fasting glucose; T2D, type 2 diabetes. Green: down-regulation; Red: up-regulation; Grey: not detected. Predictions: miR-144/*IRS1*; miR-146a/*PTPN1*; miR-150/*GLUT4* and *CBL*; miR-182/*FOXO1*; miR-192/*INSR*; miR-30d/*INS*; miR-29a and miR-320/*AKT2*.

The eight miRNAs identified could potentially form a signature to distinguish IFG and T2D (Fig. 5A). We validated these microarray data using stem-loop real-time RT-PCR and we observed that the results were consistent with our microarray data (Fig. 5B). miR-144 and miR-192 were among the highly up-regulated miRNAs in T2D. In contrast, miR-192 expression remained close to basal level in the IFG group while miR-144 showed a significant up-regulation against healthy controls but at a lower fold change compared to that in T2D (IFG: 1.385±0.14; T2D: 3.070±0.13). miR-29a and miR-320 that have been previously found to show altered expression in diabetic models [34], [49], [50], also showed a significant up-regulation in the T2D samples in our study. miR-150, miR-182 and miR-30d showed contrasting expressions in IFG and T2D while miR-146a was down-regulated in both cases.

To gain further insight on how these eight miRNAs participate in T2D development, we used the available databases to search for their potential mRNA targets that are related to glucose homeostasis. miR-30d expression has been strongly associated to insulin gene transcription [49], while miR-320 was reported to affect the *AKT* signaling pathway [50]. In this study, we tested this hypothesis by comparing miR-30d and miR-320a expression with respect to insulin (*INS*) and *AKT* mRNA levels respectively. We next queried how the other miRNA alterations relate to mRNA expression. We searched the five best target prediction databases namely RegRNA [http://regrna.mbc.nctu.edu.tw/], MirBase [http://microrna.sanger.ac.uk/cgi-bin/targets/v5/search.pl], TargetScan [http://www.targetscan.org/], Mirgen [http://www.diana.pcbi.upenn.edu/cgi-bin/miRGen/v3/Targets.cgi] and microRNA.org [http://www.microrna.org/microrna/getGeneForm.do] to look for genes that play key roles in the insulin pathway as potential targets of the dysregulated miRNAs. Here, we considered six insulin signaling related mRNAs that encode insulin receptor (*INSR*), glucose transporter 4 (*GLUT4*), insulin receptor substrate 1 (*IRS1*), Cas-Br-M murine ecotropic retroviral transforming sequence homolog (*CBL*), forkhead box O1 (*FOXO1*) and protein-tyrosine phosphatase nonreceptor-type 1 (*PTPN1*) as potential targets for the selected miRNAs (when detected in at least three of the five databases).

To verify the relationship between miRNAs and their potential mRNA targets, we also carried out mRNA microarray on the peripheral blood of IFG and T2D individuals (Table S6 shows expression of genes of interest; GEO Accession No. GSE21321). PCA was then plotted based on the mRNA profiles to study the distribution of the participants (Table S7). We observed that microRNA profiling gives a more accurate prediction of the participants' metabolic status rather than mRNA profiling. With particular focus on the genes identified above, we compared their mRNA expressions with the corresponding predicted miRNA modulators (Table S8). Quantitative real-time PCR was used to validate the mRNA microarray results for these mRNAs. The eight mRNAs have been found to be differentially expressed in IFG and T2D (Fig. 5C). The results also corresponded to the expression patterns of their respective potential miRNA regulators. Most of them showed contrasting results between IFG and T2D, with the exception of insulin receptor substrate 1 (*IRS1*). Besides miR-192 [35] and miR-29a [34] which have previously been shown to be implicated in diabetes, we have also observed an approximately linear relationship between miR-144 expression and increasing glycaemic status (from IFG to T2D).

### Validation of miRNA and mRNA expression with another batch of patients (Batch B)

**Figure 6:**
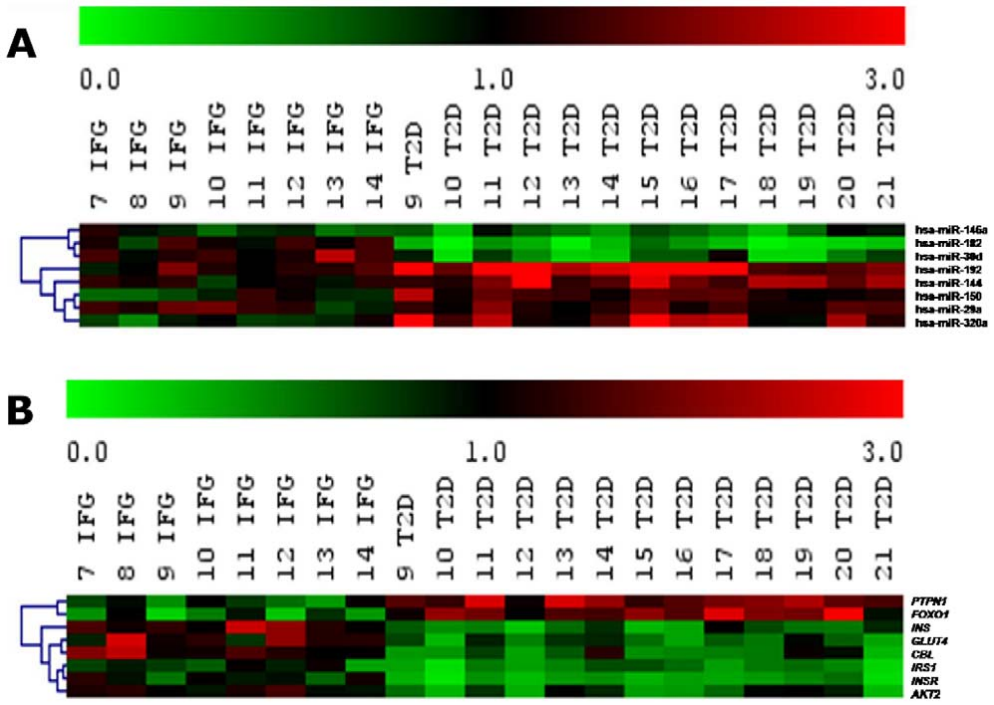
Comparison of miRNA expressions with its corresponding predicted mRNA targets (Batch B). ***A*** **. A heatmap of quantitative stem-loop RT-PCR of eight selected miRNAs and** ***B*** **. A heatmap of real-time PCR of respective predicted mRNA targets.** Validation has been done in another independent batch (Batch B) of participants in triplicates. Statistically significant differences are tested at p<0.05 significance. Batch B patients consists of eight IFG patients (labeled as 7 IFG to 14 IFG) and thirteen T2D patients (labeled as 9 T2D to 21 T2D).CTL, healthy controls; IFG, impaired fasting glucose; T2D, type 2 diabetes. Green: down-regulation; Red: up-regulation; Grey: not detected. Predictions: miR-144/*IRS1*; miR-146a/*PTPN1*; miR-150/*GLUT4* and *CBL*; miR-182/*FOXO1*; miR-192/*INSR*; miR-30d/*INS*; miR-29a and miR-320/*AKT2*.

To verify the potential signature miRNAs in IFG and T2D, we validated the expression of the selected miRNAs and mRNAs in another batch of individuals who were not on medication during the time of recruitment and were newly diagnosed with either IFG or T2D (Table 2, Batch B). Differential miRNA and mRNA expression were observed in the two cases as quantified by stem-loop real-time RT-PCR and real-time PCR (Table S9). The heatmap showed inverse relationship between miRNAs (Fig. 6A) and their potential mRNA targets (Fig. 6B) which was consistent with the observation from the previous batch (Batch A).

### miRNA and mRNA targets in the blood, tissues and exosomes of rat model

Since different tissues may have different abundance levels of the miRNAs of interest, we investigated how each organ contributes to the circulating miRNA pool we observed in the blood. To assess this, we employed stem-loop real-time RT-PCR to measure the abundance levels of miRNAs in each tissue (pancreas, liver, skeletal muscle and adipose), blood and also exosomes from control rats (Table S10). In general, miRNAs are found in greater abundance in the blood as compared to that in exosomes. This could be due to the fact that the blood contains other sources of miRNAs such as leukocytes which could contribute to the miRNA pool. miR-29a, miR-30d, miR-150 and miR-320 are all highly expressed in adipose, skeletal muscle and liver tissues with lower abundance in pancreas. miR-144, miR-146, miR-182 and miR-192 showed a lower expression compared to the other miRNAs in the three insulin target tissues as well as in the pancreas. A more uniform expression level of miRNAs is observed in the insulin-target tissues in contrast to pancreas, the insulin-secreting organ.

**Figure 7:**
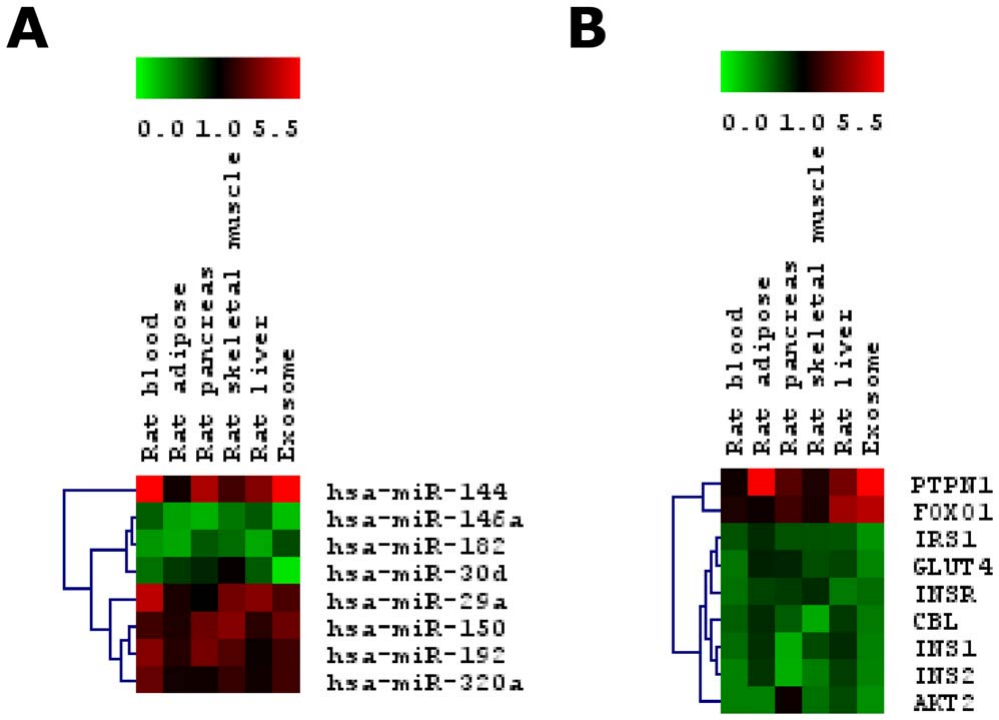
Comparison of miRNA expressions with its corresponding predicted mRNA targets (in rat's blood, tissues and exosomes). ***A*** **. A heatmap of quantitative stem-loop RT-PCR and real-time PCR of selected miRNAs and mRNAs respectively.** Samples are obtained from a combination of low dose STZ and high-fat diet induced T2D rat model. ***B*** **. A heatmap of quantitative stem-loop RT-PCR and real-time PCR of selected miRNAs and mRNAs respectively.** Samples are obtained from high-fat diet induced T2D rat model. Validation has been done in triplicates. Statistically significant differences are tested at p<0.05 significance. Green: down-regulation; Red: up-regulation; Grey: not detected. Predictions: miR-144/*IRS1*; miR-146a/*PTPN1*; miR-150/*GLUT4* and *CBL*; miR-182/*FOXO1*; miR-192/*INSR*; miR-30d/*INS*; miR-29a and miR-320/*AKT2*.

We have also quantitated the mRNA abundance levels in all tissues along with blood and exosomes (Table S10). We also measured both *INS1* and *INS2* mRNA levels since rats carry these two insulin genes. Interestingly, the expression levels of nine mRNAs (*AKT2, CBL, FOXO1, GLUT4, INS1, INS2, INSR, IRS1 and PTPN1*) was found to be highest in skeletal muscle and liver, followed by pancreas and lowest in the adipose tissue. Subsequently, when the tissues from diabetic rats were subjected to quantitative stem-loop PCR, we observed a significantly dysregulated pattern of expression for the eight miRNAs under investigation (Fig. 7A). miR-29a, miR-144, miR-150, miR-192 and miR-320 showed an up-regulation from that of control samples whereas miR-30d, miR-146a and miR-182 showed down-regulation. As expected, the expression of respective target mRNAs exhibited an inverse expression pattern (Fig.7B)

### Expression of potential ‘signature miRNAs’ in islets subjected to high glucose treatment

**Figure 8:**
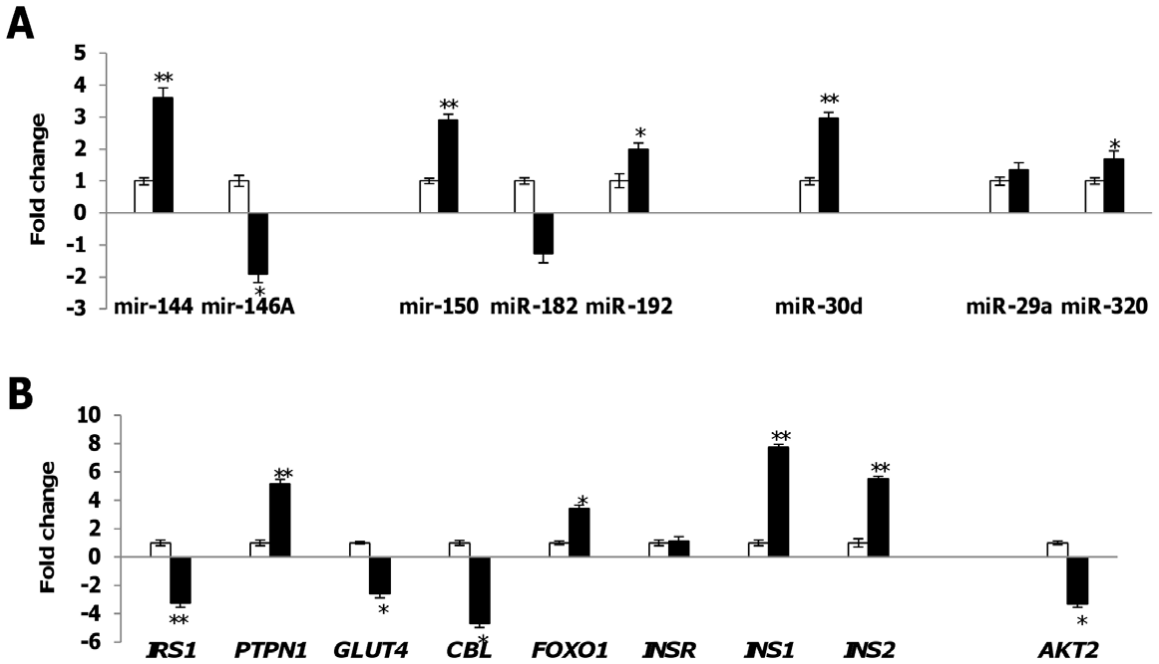
*A*. Quantitative stem-loop RT-PCR of selected miRNAs in islets with basal glucose concentration of 5 mM (white bars) and high glucose concentration of 25 mM (black bars) and *B.* Real-time PCR of respective predicted mRNA targets in with basal glucose concentration of 5 mM (white bars) and high glucose concentration of 25 mM (black bars). Data presented as mean ± SEM with n = 3 for each group. Statistically significant differences are tested at p<0.05 significance. ^*^p<0.05, **p<0.01, ***p<0.001. Predictions: miR-144/*IRS1*; miR-146a/*PTPN1*; miR-150/*GLUT4* and *CBL*; miR-182/*FOXO1*; miR-192/*INSR*; miR-30d/*INS*; miR-29a and miR-320/*AKT2*.

In addition to animal and human studies, we also performed primary cell culture studies using islets isolated from the pancreas of normal rats. These islets were then subjected to two different glucose concentrations; 5 mM (basal glucose) and 25 mM (high glucose). Total RNA extracted from these two treatment groups were then used to quantitate the eight miRNAs under study along with their predicted mRNA targets (Fig. 8, Table S11). In contrast to our previous observations, miR-30d was found to be highly up-regulated (2.969±0.18) which corresponded to increased expression of *INS1* and *INS2*, 7.757±0.19 and 5.500±0.21 respectively in response to high glucose condition. This difference could be due to the early onset of insulin deficiency in T2D in which β-cell dysfunction has not fully occurred like in the case of our animal model where low dose of STZ could initiate β-cell destruction as observed in overt T2D. Correspondingly, in the high glucose media, expression of miR-144 was found to be highest (3.605±0.21) accompanied by a down-regulation of *IRS1* (−3.257±0.29), followed by miR-150 (2.904±0.19) and miR-192 (1.989±0.20) which are predicted to target GLUT4 (−2.604±0.25) and CBL (−4.672±0.31) and INSR (1.150±0.28) respectively. Likewise, down-regulation of miR-146a (1.905±0.26) was followed by an increased expression of its target mRNA, PTPN1 (5.175±0.28). Marginal expression levels were observed for miR-182 (−1.267±0.26) and miR-29a (1.351±0.23) which inversely correlated to their respective predicted mRNA targets FOXO1 and AKT2 respectively.

### Differential expression of IRS-1 and phosphorylated IRS-1 in IFG and T2D

**Figure 9:**
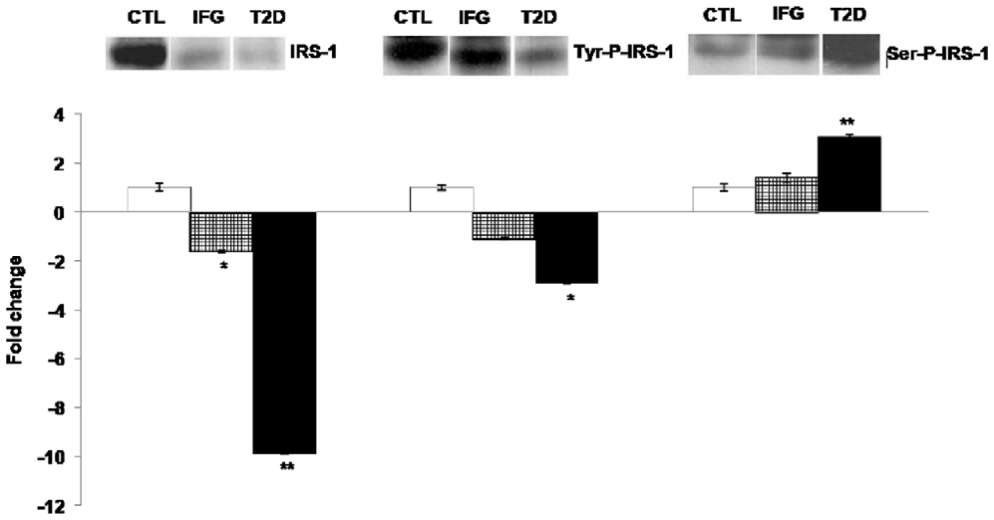
Western Blot analysis of IRS1, Tyr^612^-phospho-IRS1 and Ser^636+639^-phospho-IRS1 in CTL (white bars), IFG (checkered bars) and T2D (black bars) individuals. The data presented here is a representative of three independent experiments (both individual and pooled serum samples showed similar profiles) and as mean ± SEM. Fold change below one are expressed as negative values. Statistically significant differences are tested at p<0.05 significance. ^*^p<0.05, **p<0.01, ***p<0.001. CTL, healthy controls; IFG, impaired fasting glucose; T2D, type 2 diabetes.

Since insulin-dependent glucose uptake could be compromised by the over expression of miR-144, we further investigated miR-144 and its predicted target, *IRS1*. We compared the IRS1 protein expression in the two case groups against normal control. As phosphorylation leads to IRS1 activation, we examined the protein content for Tyr^612^-phospho-IRS1 and Ser^636+639^-phospho-IRS1. These two sites of phosphorylation are selected considering their opposing effects on IRS1 activation. While the phosphorylation of IRS1 on tyrosine residues is needed to mediate insulin signaling, phosphorylation of IRS1 on certain serine residues (Ser-636 and Ser-639) have been shown to terminate insulin effects [53], [54]. As shown in Fig. 9, IRS1 protein content displayed a decreasing expression from healthy control to disease groups with T2D. This can imply that IRS1 is highly down-regulated in T2D in conjunction with its predicted negative modulator, miR-144 which was up-regulated in T2D. Western blot of serum samples showed a decreased expression of Tyr^612^-P-IRS1 protein in T2D as compared to that of CTL and IFG, while the expression of Ser^636+639^-P-IRS1 in T2D was highest among the three groups (Fig. 9).

### IRS1 is a direct target of miR-144

**Figure 10:**
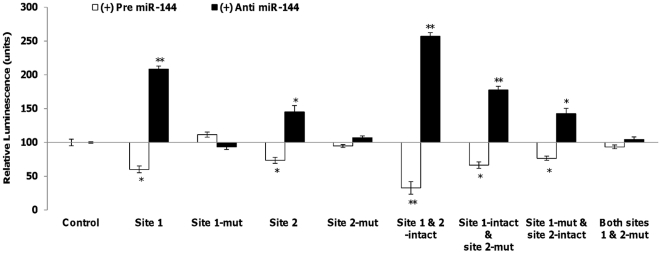
Direct inhibitory effect of miR-144 on *IRS1*. Dual luciferase assay. Quantitation of the effects of pre- or anti- miR-144 interaction with the 3′ UTR of *IRS1*. Binding sites of miR-144 at 3′UTR of *IRS1* and mutated constructs of the binding sites are listed in Table S12. The plasmid constructs together with pre- or anti- miR-144 were co-transfected into HeLa cells. Luminescence for luciferase gene activity in treated samples (pre or anti miR-144) were obtained 48 hours post-transfection. Relative luminescence was obtained by normalizing the values against control plasmids, pMIR-REPORT™ without any 3′UTR insert. Data presented as mean ± SEM with n = 3. Fold change below one are expressed as the negative values. Statistically significant differences are tested at p<0.05 significance. ^*^p<0.05, **p<0.01, ***p<0.001.

There are two different binding sites of miR-144 at the 3′ UTR of *IRS1*. Table S12 shows the bindings sites of miR-144 at the 3′UTR of *IRS1* and the different constructs designed to show that miR-144 directly targets *IRS1*. To demonstrate that *IRS1* is a direct target of miR-144, we constructed independent reporter vectors that contained the different combinations of binding sites of miR-144 at the 3′UTR of *IRS1* (Table S12) downstream of a firefly luciferase reporter gene. These constructs were then co-transfected with anti- or pre-miR-144 into HeLa cells. Cells transfected with pre-miR-144 exhibited a significant reduction in the luciferase activity which was reduced further when both miR-144 binding sites are present (Fig. 10). Likewise, transfection with anti miR-144 showed an increasing pattern of luciferase activity from constructs where only one binding site is present to that where both binding sites are included (Fig. 10). Site-directed mutagenesis of the miR-144 binding sites abolished interactions between miR-144 and IRS1 accordingly. These findings indicate *IRS1* forms a direct target of miR-144.

### miR-144 regulates IRS1 mRNA expression

**Figure 11:**
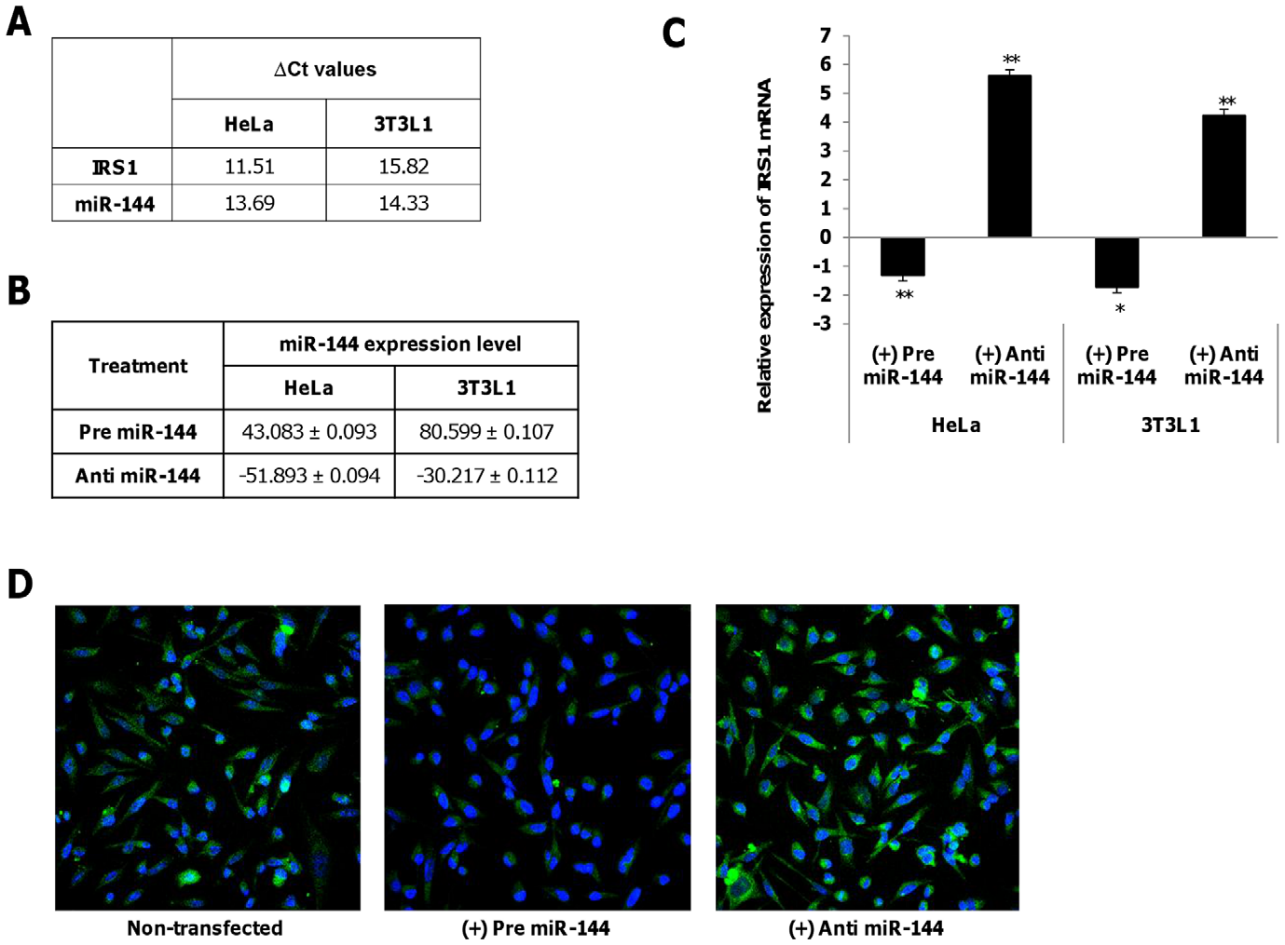
Expression of IRS1 in HeLa/3T3L1 cells transfected with pre- or anti- miR-144. ***A.*** **Endogenous levels of miR-144 and** ***IRS1*** **in HeLa/3T3L1 cells as delta threshold cycle (ΔCt) value with respect to 18S rRNA.** Previous reports [42]–[44] have also confirmed that miR-144 and IRS1 are expressed in HeLa cells. ***B.*** **Relative miR-144 expression in HeLa/3T3L1 cells transfected with either pre- or anti- miR-144 at a concentration of 30 nM.** ***C.*** **Relative** ***IRS1*** **mRNA expression in HeLa/3T3L1 cells transfected with either pre- or anti- miR-144at a concentration of 30 nM.** Data presented as mean ± SEM (n = 3). Fold change below one are expressed as the negative values. Statistically significant differences are tested at p<0.05 significance. ^*^p<0.05, **p<0.01, ***p<0.001. ***D.*** **IRS1 immunoreactives in HeLa cells transfected with either pre- or anti- miR-144.** The cells were fixed and immunolabeled with IRS1 antibody (green) and nuclear stain DAPI (blue). Left panel: Non-transfected cells; Middle panel: Pre miR-144 transfected cells showed reduced fluorescence intensity; Right panel: Anti miR-144 transfected cells showed increased fluorescence intensity. The data presented here is a representative of three independent experiments.

Previous reports [55]–[57] have confirmed that HeLa cells express miR-144 and *IRS1*. HeLa cells were then used to assess the regulatory function of miR-144 on *IRS1* and in addition to that, we also performed the same experiment in an insulin-responsive cell line, 3T3L1 adipocytes. Basal levels of miR-144 and IRS1 were measured in both cell lines (Fig. 11A) prior to our subsequent assays. Based on the prediction that miR-144 binds to the 3′UTR of *IRS1*, changes in miR-144 expression should be reflected in *IRS1* mRNA levels accordingly. In order to alter miR-144 expression, HeLa and 3T3L1 cells were transfected with pre-miR-144 and anti-miR-144 independently, followed by quantification of the changes in miRNA and mRNA levels (Fig. 11B, 11C). Introduction of pre-miR-144 increased the miR-144 levels by 43 fold and simultaneous reduction in the *IRS1* mRNA expression (−1.628±0.18) in HeLa. In 3T3L1 adipocytes treated with pre-miR-144, miR-144 levels increased by approximately 80 fold which corresponded to decreased IRS1 mRNA level (−1.737±0.18). Likewise, in anti-miR-144 transfected HeLa and 3T3L1 cells, the relative expression of miR-144 was reduced to 0.019±0.09 and 0.033±0.11 respectively while a significant increase in *IRS1* mRNA level was observed (HeLa: 5.607±0.217; 3T3L1: 4.232±0.22). Regulation of *IRS1* by miR-144 was further verified by immunocytochemistry and complementarily, the *IRS1* protein expression correlated to that of the gene expression studies. Pre-miR-144 transfected HeLa cells displayed reduced fluorescence intensity for *IRS1* (Fig. 11D, middle panel) while the opposite was observed in anti-miR-144 transfected cells (Fig. 11D, right panel). Altogether, these results suggest that miR-144 is a modulator of *IRS1*.

## Discussion

### Circulating blood miRNAs are reflective of those in tissues

It has been reported that miRNAs circulating in the blood can potentially serve as novel noninvasive biomarkers of diseases including diabetes [13]–[19]. This hypothesis is supported by a recent study by Kosaka *et al* [22] which generated the concept of miRNAs-mediated intercellular communication. He reported that circulatory miRNAs are released through a secretory mechanism and conveyed to recipient cells to exert their function. Furthermore, Laterza *et al* [21] have demonstrated that aberrant expression of circulating miRNAs corresponds to (origin) tissue injury. In our T2D animal model, we profiled miRNA expression in pancreas, three insulin-targeted tissues (adipose, skeletal muscle and liver) as well as blood to observe the relevance of blood miRNAs to those in tissues. A similar study has also been carried out Zhao *et al* [38], in which the group performed miRNA profiling in the islets, liver and adipose of diabetes-resistant (B6) and diabetes-susceptible (BTBR) mice. Zhao *et al* identified different groups of miRNAs in different organs which could potentially participate in the pathogenesis of T2D. Some of the miRNAs identified by them showed similar expression pattern in our study as well. miRNAs such as miR-133a, miR-185, miR-152, miR-34a and miR-342 which were reported to show increased expression were also up-regulated in our rat model. While miR-184 and miR-204 which were down-regulated in at least one mouse strain in their study, also showed down-regulation in our model. However, there were striking differences in the expression of miRNAs such as miR-135a, miR-429, miR-200a/b and miR-215 which were not detected in our samples but were reported to be significantly decreased by Zhao *et al* [38]. A possible reason for these differences could be that these miRNAs were reduced to a level that is beyond detection. Alternatively, the different species and methodology being used to induce T2D could also explain the differences. Zhao *et al* used the genetic modification approach while we stimulated T2D by using a combination of high-fat diet and low dose STZ (40 mg/kg). As indicated in the heatmap (Fig. 2A), the rat blood miRNA profile was clustered closely to those from the insulin-targeted tissues. Furthermore the Venn diagram (Fig. 2B) showed that most of the miRNA changes detected in the tissues involved in the insulin signaling pathway can also be detected in the blood miRNA profile. These findings strengthened the observations made by Kosaka [22] and Laterza [21] that miRNAs can interact intercellularly and that tissue injury may result in the secretion of miRNAs. These miRNAs that are packaged in exosomes are known to be nuclease resistant and stable in peripheral blood [27]. Based on these reports, we extended our investigation to profile the miRNAs in human blood samples.

### miRNAs involved in the insulin signaling pathway

**Figure 12:**
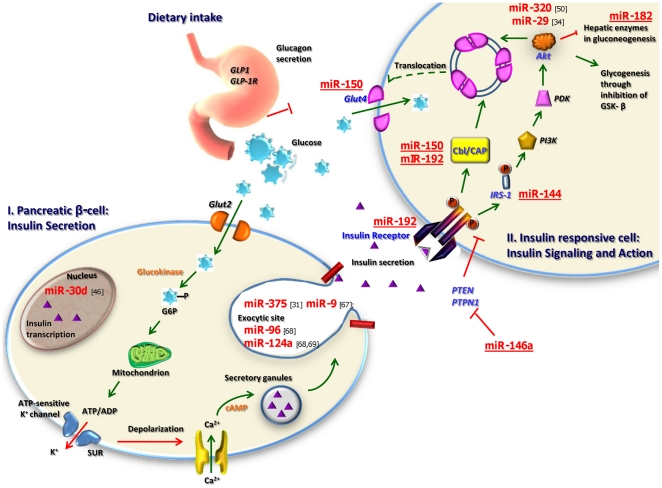
Possible mode of mechanism on miRNA-regulatory networks. Possible ways in which miRNA govern different stages of the insulin pathway in the pathogenesis of Type 2 Diabetes. miRNAs that have been reported in previous studies are indicated in brackets while those underlined are predicted by the five best target prediction databases namely RegRNA, MirBase, TargetScan, Mirgen and microRNA.org.

The first part of our investigation in T2D animal model study revealed eight miRNAs that were highly dysregulated in T2D. These eight miRNAs showed similar expression across all five sources (pancreas, liver, skeletal muscle, adipose and blood) that were profiled. Among these eight miRNAs, four of them namely miR-192 [35], miR-29a [34], miR-30d [49] and miR-320a [50], [51] had previously been reported in earlier studies and our results were consistent with them. Among the novel miRNAs (miR-144, miR-146a, miR-150 and miR-182) identified, miR-144 had the highest up-regulation upon T2D in most tissues. However during the course of our study, involvement of miR-146a in diabetes was reported by another group of researchers along with other diabetes-related miRNAs [58], [59]. Following that, we carried out a cross-sectional study involving Asian males and found these shortlisted eight miRNAs showing differential expression patterns in the blood samples of IFG and T2D patients. Hence these miRNAs could correspond to the disease development in these individuals. Similar to the animal data, miR-144 was again among the highly up-regulated miRNA that showed a linear increase in expression with increasing glycaemic status. With the help of published reports and bioinformatics-based databases, we identified the potential insulin signaling-related mRNA targets for these miRNAs (Fig. 5B, 5C). Based on these findings we have proposed a mechanism on how circulating miRNAs may potentially control the expression corresponding targets and how their regulatory networks may govern different stages of the insulin pathway in the pathogenesis of T2D (Fig. 12) [31], [34], [46], [50], [67]–[69]. miRNAs such as miR-144, miR-29a and miR-192 that were up-regulated in both IFG and T2D patients (Table S8) are predicted to target *IRS1*, *AKT2* and *INSR* respectively. These genes are involved in the insulin signaling pathway. Of the above three, only the expression pattern for miR-144 and its predicted mRNA target, *IRS1*, are concordant for both IFG and T2D (Fig. 5B, 5C). miR-144 also exhibited an approximately linear relationship with increasing glycaemic status as observed in IFG and T2D patients (Fig. 5B). We hypothesize that this may represent the primary abnormality in the pathogenesis of development of abnormal glucose tolerance. The changes in expression patterns observed in the other mRNA targets we identified (comparing IFG and T2D with healthy controls) are either inconsistent or only significantly different in T2D but not IFG (Fig. 5B, 5C). This may be due to a secondary effect that could have resulted from the primary abnormality of down-regulation of *IRS1*. The differences in expression patterns in IFG and T2D may represent the loss or impaired adaptive changes to increased blood glucose in T2D, ultimately resulting in hyperglycemia. miR-30d and miR-320 which have been shown to regulate *INS* and *PIK3/Akt* signaling [50] exhibited opposite expression in the two disease groups (Table S8). Insulin production mainly involves insulin gene transcription which is indicated by the insulin mRNA levels. The quantitative real-time PCR results showed that *INS* mRNA increased in IFG but was negatively regulated in T2D (Fig. 5C, 6B). This supports the transition of hyperinsulinemia to relative insulin deficiency seen in patients with IFG and T2D respectively. Inhibition of miR-30d has been found to abolish glucose stimulated insulin gene transcription in cell culture studies [49]. Our data is in support of this theory in which reduced expression of miR-30d correlates with down-regulation of insulin gene in the T2D samples, while, up-regulation of miR-30d in the IFG group is accompanied with increased levels of insulin mRNA (Fig. 5B, 6A). Our data suggests unlike most other miRNAs which are inhibitory, miR-30d may be functioning as an RNA activator to insulin gene expression. The final stage of insulin signaling is the translocation of glucose transporter-4 (*GLUT4*) from the intracellular storage to the plasma membrane of the insulin responsive cells to allow uptake of glucose [60]. V-akt murine thymoma viral oncogene homolog 2 (*AKT2*) and Cas-Br-M (murine) ecotropic retroviral transforming sequence (*CBL*) are two molecules responsible for this event. We found mRNA for *AKT2* to be maintained at basal level in IFG but greatly reduced in T2D (Fig. 5C, 6B). This differential expression suggests a mechanism in which, insulin signaling is impaired in T2D. On the other hand, *CBL* (a second cue for *GLUT4* translocation) was up-regulated in IFG but reduced in T2D (Fig. 5C, 6B). This could imply a compensatory mechanism to maintain effective *GLUT4* translocation in IFG, lost in T2D. These are associated with the down-regulation of *GLUT4*, a key effector of insulin action, in T2D. *CBL* and *GLUT4* are potential targets of miR-150, while miR-320 inhibits *PI3-K/Akt* signaling [50]. Once again, the inverse pattern between miRNA and their mRNA target expression is demonstrated by both miR-150 and miR-320a up-regulation in T2D subjects (Fig. 5B, 6A).

Binding of insulin to insulin receptor may be suppressed by several inhibitors such as *PTPN1*, a potential mRNA target of miR-146a. When expressed at normal levels, this miRNA may act as a suppressor of *PTPN1* to allow insulin signaling. However, reduced expression of miR-146a may lift the suppression of *PTPN1* gene, allowing the latter to impair insulin signaling. We have found that down-regulation of miR-146a to be greater in T2D than IFG (Fig. 5B, 6A). This observation correlates with the expression of *PTPN1* which remained at basal level in IFG but increased in T2D (Fig. 5C, 6B). Increased levels of *PTPN1* will inhibit insulin signaling by removing phosphate groups of the effector molecules thus aborting the downstream events. Hence these observations further suggest that insulin signaling is inhibited to a greater degree in T2D, even in newly diagnosed stage, as in our study.

In addition to promoting glucose uptake, insulin inhibits production and release of glucose by blocking hepatic gluconeogenesis. Forkhead box O1 (*FOXO1*), a member of the forkhead family of transcription factors, plays a role in hepatic enzyme regulation in gluconeogenesis. We found miR-182, a potential modulator of *FOXO1* to be down-regulated in T2D but was slightly up-regulated in IFG (Fig. 5B, 6A). Correspondingly, *FOXO1* mRNA was negatively regulated in IFG while showing positive fold change in T2D (Fig. 5C, 6B), suggesting hepatic gluconeogenesis to have a compensatory reduction in IFG, but increased in T2D.

Although our results were in conjunction with earlier studies which identified miR-192, miR-29a, miR-320 and miR-30d to be potential key players in the pathogenesis of T2D, we do observe differences in the recent study reported by Zampetaki *et al* [20]. The authors discussed the loss of endothelial miR-126 and other miRNAs in T2D. Among the miRNAs they identified, similarities in miRNA changes were observed in miR-20b, miR-191 and miR-197 which were down-regulated. We also observe the same in almost all of our IFG samples and more than 50% of our T2D samples. Zampetaki *et al* [20] identified miR-126 as a characteristic miRNA in T2D and demonstrated that it is involved in endothelial dysfunction. We do not observe a similar down-regulation of miR-126 in our samples. This could be explained by the fact that we have exclusively used subjects that were not on any medications and do not show abnormalities in cholesterol, blood pressure and BMI. A separate study by Gallagher *et al* [61] performed miRNA profiling using total RNA extracted from muscle tissue biopsies of individuals with insulin resistance. The samples that were used by Gallagher *et al* were different from ours in terms of ethnicity and BMI (individuals in Gallagher *et al* study are considered obese), also some of them were receiving medications. These differences could again contribute to the discrepancies in our results. Nonetheless, the authors identified a pool of dysregulated miRNAs (∼50 miRNAs) in which some of them such as miR-144, miR-106b, miR-185, miR-30e, miR-589, miR-665 and many more showed similarities in expression as observed in our study (either humans/animals or both). Moreover, miR-144 was also found to be the most highly expressed miRNA in their T2D samples. Our study correlated quite closely with another study reported recently by Kong *et al* [59] which is based on a Chinese population. The authors investigated the expression of seven diabetes-related miRNAs (miR-9, miR-29a, miR-30d, miR-34a, miR-124a, miR-146a and miR-375), four (miR-29a, miR-30d, miR-175 and miR-146a) of which were also found to be dysregulated in our study.

### Exosomal miRNAs and mRNAs

Several studies have reported how microvesicles such as exosomes could serve as a transport machinery of miRNAs, mRNAs and proteins [27] to mediate cell-to-cell communication. In our study, the whole blood was used as a source of miRNAs since it will include all possible sources of circulating miRNAs such as the leukocytes, plasma, serum and exosomes. Additionally, we also studied the expression of the potential ‘signature miRNAs’ of T2D in exosomes isolated from serum of our rat T2D model. As expected, the expression pattern of exosomal miRNAs was shown to be consistent with that we observed in whole blood but at a lower level. This could be due to the whole blood encompassing the other sources of miRNAs apart from those of exosomes alone.

### miR-144 as an important key player in T2D

Among the eight miRNAs identified as potential signature miRNAs, miR-144 has shown to be a potential novel key player in T2D. Here, miR-144 expression is highly up-regulated in T2D and it also seemed to exhibit a linear relationship with increasing glycaemic status (Fig. 5B, 6A). Focusing our interest in miR-144, we next looked into its corresponding *IRS1* target at protein level. Using Western blot analysis, we have clearly demonstrated a decreased IRS1 protein content in IFG and T2D (Fig. 9). This result is in conjunction with our hypothesis that miR-144 negatively modulates *IRS1*. Tyr^612^-phospho-IRS1 was markedly reduced in T2D, while Ser^636+639^-phospho-IRS1, responsible in inhibiting the insulin signaling cascade [53], [54] was increased in T2D (Fig. 9).

To confirm that *IRS1* is a target of miR-144, we observed the corresponding changes in IRS1 mRNA level upon treatment with either pre- or anti- miR-144 (Fig. 11B, 11C). Introduction of pre-miR-144 greatly inhibited *IRS1* mRNA level while treatment with anti-miR-144 resulted in increased *IRS1* mRNA expression (Fig. 11C). Similar observations were obtained in the immunocytochemistry assay which indicates that inhibition of *IRS1* by miR-144 at protein level (Fig. 11D). These observations together with the luciferase binding assays (Fig. 10) demonstrate that miR-144 functions as a modulator of *IRS1* expression. Hence, miR-144 could serve as a potential therapeutic target in T2D.

In our study, we have shown that circulating blood miRNAs are appropriate indicators of disease development. We have also identified eight important miRNAs (miR-144, miR-146a, miR-150, miR-182, miR-192, mir-29a, miR-30d and miR-320) that could participate in the regulation of insulin signaling as well as useful in distinguishing different stages of diabetes progression. These findings can contribute to the existing knowledge on the role of miRNAs in glucose metabolism, diabetes and its related complications. Among these miRNAs, we have identified miR-144 as a direct modulator of *IRS1* and hence a potential therapeutic target of T2D. Nonetheless, further investigations using animal models as well as more human samples have to be carried out to validate our findings and to further elucidate the mechanisms of miRNA regulatory network in the pathogenesis of T2D.

## Methods

### Animal care and sample collection

Male Wistar rats, 6 weeks of age and weighing approximately 150 g were used for all studies. The animals were handled according to the guidelines of the Council for International Organization of Medical Sciences on Animal Experimentation (World Health Organization, Geneva, Switzerland) and the National University of Singapore. The animal protocols were approved (approved code 062/09) by the National University of Singapore Institutional Animal Care and Use committee. Induction of Type 2 diabetes was done according to Reed *et al* [45] with some modifications. After one week of acclimatization, twelve rats were randomly divided into two groups equally. One group was fed with normal fat diet (NFD) consisting of (as percentage of total kcal) 18% fat, 58% carbohydrate and 24% protein (Harlan Teklad, 2018, Madison, WI) and the other with high fat diet (HFD) consisting of 39% fat, 40% carbohydrate and 21% protein (Specialty Feeds, SF10-019, Western Australia). After two weeks the animals on HFD were injected with low dose of STZ (40 mg/kg) intra-peritoneally after an overnight fast. Both groups of animals were continued on their respective diets (NFD or HFD) for another week [43], [45]. A combination of high fat diet and low dose of STZ has been found to show characteristics closely related to T2D in contrast to administration of high STZ alone which mimics Type 1 diabetes [46]–[48]. At the end of the experimental period (on the 8^th^ day after STZ administration), animals were euthanized using CO_2_ and blood and tissue samples (pancreas, liver, adipose and skeletal muscle) were harvested and washed in cold saline. Serum was separated by centrifugation and analyzed for concentrations of glucose, insulin and triglycerides. Exosomes were isolated from serum samples using ExoQuick™ exosome precipitation kit (SystemBio, CA, USA) followed by exosomal total RNA isolation according to the manufacturer's protocol. Both blood and tissue samples were preserved at −80°C for subsequent analysis.

### Oral glucose tolerance test

To assess oral glucose tolerance, animals were fasted overnight and their serum glucose response to the oral administration (by oral gavage) of a solution of 20% glucose (2 g/kg) was determined. Blood was drawn from tail at time 0, 15, 30, 60 and 120 minutes after administration of glucose. Glucose and insulin concentrations were analyzed.

### Islet isolation and culture

Pancreatic islets were isolated by collagenase digestion as previously described [62], [63]. Briefly, the pancreas was perfused with a solution of 0.5 mg/ml Collagenase Type V (Sigma, USA) dissolved in Hanks Balanced Salt Solution (HBSS). The digestion was performed at 37°C for 20–30 min after which the collagenase was neutralized with HBSS supplemented with 1% FBS. The collagenase treated pancreas was then sequentially filtered through 1.5 mm and 0.8 mm metal mesh filters. Islets were subsequently enriched by centrifugation in Histopaque 1077 (Sigma, USA) and hand-picked under direct light microscopic visualization. Isolated islets were cultured in 24-well plate at 100 islets per well containing DMEM medium (Sigma, USA) supplemented with 2 mM L-glutamine (Gibco, USA), 10% fetal bovine serum (Gibco, USA), 100 U/ml penicillin and 100 µg/ml streptomycin (Pen-Strep; Bio-Whittaker Europe, Verviers, Belgium), and 5.0 mmol/l glucose. After 24 h incubation at basal glucose concentration (5.0mmol/l), islets were transferred to either basal (5.0 mM) or high (25.0 mM) glucose in DMEM for 24 h. Total RNA was isolated from islets using the Trizol reagent according to the manufacturer's protocol.

### Patients

We consecutively enrolled all consenting male adult subjects (21 to 70 years of age) with no past medical history, seen in Alexandra Hospital (Singapore) for health screening during the period of July 2008 to April 2009. This study was approved by our institutional ethics review board (Ministry of Health, Singapore; MH95:03/1–11) and all volunteers signed written informed consent. All subjects were not on any medications and health-screening involved physical examination including waist circumference, other anthropometric measures and blood pressure. Blood was collected in fluoride oxalate tubes after an overnight fast of 10 to 12 hours. Plasma glucose was obtained by enzymatic methods (ADVIA 2400, Bayer Diagnostics), while serum total cholesterol (TC), triglyceride (TG), low-density lipoprotein (LDL) and high-density lipoprotein (HDL) levels were measured using an automated analyzer (ADVIA 2400, Bayer Diagnostics). Blood samples were then categorized into 3 distinct groups, based on the World Health Organization (WHO) and International Diabetes Federation (IDF) [64]: Healthy controls (CTL, n = 15) with fasting glucose <6.1 mmol/L, Impaired Fasting Glucose (IFG, n = 14) with fasting glucose above 6.0mmol/l but less than 7.0 mmol/L and Type 2 Diabetes (T2D, n = 21) with fasting glucose greater or equal to 7.0mmol/L (Table 2- Batches A and B). All were normotensive (blood pressure ≤140/90), with desirable cholesterol levels (LDL ≤3.4 mmol/L) and body mass index (BMI ≤27 which indicates that they are not obese) [52]. For miRNA studies, whole blood samples collected separately in tubes containing RNALater were used. miRNA signatures in Batch A samples were confirmed independently by quantitative real-time PCR with samples from Batch B. All controls were age-matched to the respective cohort (Table 2).

### Total RNA Isolation

Total RNA (+ miRNAs) was isolated using a modification of the RiboPure™-Blood kit from Ambion (Austin,TX) according to the manufacturer's protocol. The concentration of total RNA and integrity were determined by using Nano-Drop ND-1000 Spectrophotometry (NanoDrop Tech, Rockland, Del) and gel electrophoresis respectively.

### mRNA and microRNA microarray

The oligonucleotide (DNA) microarray was performed according to manufacturer's protocol (Illumina, SanDiego, USA) using 500 ng of total RNA. Biotin-labelled complementary RNA probes were synthesized with the Illumina TotalPrep RNA Amplification kit (Ambion). Gene profiling was performed with Sentrix BeadChip Array HumanRef-8 v2 (Illumina). Data were analysed with the BeadStudio software. Differentially expressed genes were selected based on the following parameters as suggested by the manufacturers were used: p value <0.05, Diff score >20, Average signal >100. LNA-modified oligonucleotide (Exiqon, USA) probes for human, mouse and rat miRNAs annotated in miRBase version 11.0 were used in the microarray. Total RNA (1 µg) was 3′-end –labeled with Hy3 dye using the miRCURY LNA ™ Power Labeling Kit (Exiqon, USA) and hybridized on miRCURY LNA™ Arrays, using MAUI® hybridization system. Background-subtracted mean intensity of 300 was selected as a threshold value before normalization against the U6 snRNA [65]. For comparisons of miRNA profiles, data were expressed as fold change of case versus healthy control. Hierarchical clustering plot (heatmap) and Principal Component Analysis (PCA) were generated as shown by Tan *et al* [18], using TIGR multiple experimental viewer software. Microarray data were deposited in Gene Expression Omnibus (GEO) with accession numbers stated accordingly under the ‘Results’ section.

### Stem-loop real-time RT-PCR and real-time PCR

The miRNA microarray results were validated with stem-loop real-time RT-PCR [66], [67]. Eight miRNAs were selected for this validation using specific stem-loop primers (Applied Biosystems, USA). Expression of corresponding predicted mRNA targets to these selected miRNAs were also validated by real-time PCR using gene specific primers. Both reverse transcription and PCRs were performed in triplicates in at least 3 independent experiments on an Applied Biosystems 7900 sequence detection system.

### SDS-PAGE and Western Blot Analysis

Serum samples (40 ug) depleted of albumin and IgG were resolved using 10% SDS-PAGE. Western blot was carried out as described in Jeyaseelan *et al* [66]. The primary rabbit polyclonal anti-human antibodies against IRS1, Tyr^612^-phospho-IRS1 and Ser^636+639^-phospho-IRS1 were added at a 1∶2000 dilution in PBST containing 2.5% non-fat dry milk for 60 min to identify specific proteins. The membranes were washed in PBST followed by incubation in secondary goat anti-rabbit antibody (1∶5000 dilution) in PBST containing 2.5% non-fat dry milk for 60 min. The membranes were washed again with PBST and visualized with Super-Signal West-Dura Extended Duration Substrate (Pierce, Biotechnology, USA) and developed in Kodak Biomax film. Films of Western blots were scanned (Acer SWZ3300U), and the labeling intensities of the bands were quantified using ImageJ software (National Institutes of Health). Relative expression of protein is the quantity of band intensity expressed as a proportion to that in control samples. All band images were representatives of at least three independent experiments.

### Dual Luciferase reporter assay

HeLa cells were used in our immunocytochemistry and dual luciferase reporter assays [55]–[57]. The 3′UTR of *IRS1* containing the putative target site for miR-144 was amplified by PCR using gene specific primers. PCR products were cloned into *Firefly*-luc-expressing vector pMIR-REPORT™, Ambion (Austin,TX)) at the Spe1/HindIII site. Plasmid transfection procedure was adapted from Cheng *et al* [55]. HeLa cells cultured in 24-well plates were transfected with 50 nM anti miR-144 or pre miR-144 for 3hrs followed by 200 ng/well pMIR-REPORT constructs for 3 hrs. The cells were left to recover for 48 hrs before being lysed for measurement of luciferase activity. Dual luciferase assay (Promega, USA) was used to quantitate the effects of anti or pre miR-144 interaction with the 3′ UTR of *IRS1*. The assay was performed according to manufacturer's protocol. In all experiments, transfection efficiencies were normalized to those of cells co-transfected with the *Renilla*-luc-expressing vector pRL-CMV (Promega, USA) at 10 ng/well.

### Immunocytochemistry

Immunocytochemistry was also performed on HeLa cells transfected with either pre miR-144 or anti miR-144 [66]. The cells were fixed with 4% paraformaldehyde in PBS and blocked with 5% FBS before incubation with rabbit anti-human antibody IRS1 for 1 h at room temperature. Goat anti-rabbit FITC labeled secondary antibody was used. Cells were mounted and nuclei were counterstained with Hoechst 33342 and viewed under a fluorescence microscope (Carl Zeiss LSM 510 META).

### Statistical analysis

Microarray Analysis was carried out according to our previously published report [18] which involved multiple sample analysis including background subtraction, *t*-Test/One-way ANOVA analysis, hierarchical clustering and principal component analysis. Normalization was performed against U6 snRNA [18]. *t*-Test was performed between “control” and “test” sample groups and t-values were calculated for each miRNA. *p*-values were computed from the theoretical t-distribution. The microarray data reported in this manuscript is described in accordance with MIAME guidelines. The clustering using hierarchical method was performed with average linkage and Euclidean distance metric. The clustering and principal component analysis (PCA) plot was generated using TIGR MeV (Multiple Experimental Viewer) software [18]. Other statistical data evaluation was performed using two-tailed t-tests or in case of multiple comparisons using One-way ANOVA along with Fisher's least significance difference test [65]. The significance level was *p*<0.05.

## Supporting Information

Table S1 **MicroRNA microarray results of T2D rat model (** **Fig. 2A** **).** Only miRNAs that are conserved in both human and rats, and with background subtracted mean signal intensities above 300 are included. Filtered signal intensities were normalized against U6snRNA. Values shown are fold changes calculated as a ratio of T2D versus control ± SEM. The grey boxes indicate negative expression values. Array data can be accessed from Gene Expression Omnibus (GEO) with accession no. GSE26167; SuperSeries GSE26168. T2D, type 2 diabetes.
(DOC)

Table S2 **microRNAs expression in rat blood, liver, pancreas, skeletal muscle and adipose tissues.** miRNAs that were filtered using the statistical analysis as described in the “Methods section” were further subjected to fold change analysis. Statistically significant differences are tested using Student's t-test at p<0.05 significance Fold change values are calculated as a ratio of T2D versus respective control ± SEM. Fold change values below 1 are expressed in negative values. miRNAs that exhibited at least±1.5 fold change have been included. miRNAs that are highly up/down regulated in all sources are marked in bold. Only miRNAs conserved in both humans and rats are shown. T2D, type 2 diabetes.
(DOC)

Table S3 **MicroRNA microarray results of both individual and pooled samples (** **Fig. 4A** **).** miRNA that were filtered using the statistical analysis as described in the “Methods section” were further subjected to fold change analysis. Filtered signal intensities were normalized against U6snRNA. Values are fold changes calculated as a ratio of IFG or T2D versus control (CTL) ± SEM. Array have been deposited in the Gene Expression Omnibus (GEO) database and can be accessed with accession no. GSE21321; SuperSeries GSE26168. IFG, impaired fasting glucose; T2D, type 2 diabetes.
(XLS)

Table S4 **miRNAs expressed in IFG and T2D from batch A.** miRNAs whose significant changes replicated in at least 50% of the subjects in any of the two categories (IFG; n = 6, or T2D; n = 8) as compared to CTL; (n = 7) are shown. Values of miRNAs that were differentially expressed but showed no significant fold change (p>0.05) in at least 50% of the subjects in any of the two categories were not included. Values are fold changes calculated as a ratio of IFG or T2D versus control (CTL) with p-values in 2 decimal places. Fold change values below 1 are expressed as the negative values. Statistically significant differences are tested using Student's t-test at p<0.05 significance. miRNAs that are significantly expressed in both IFG and T2D are shown in bold. CTL, healthy controls; IFG, impaired fasting glucose; T2D, type 2 diabetes.
(DOC)

Table S5 **Pearson correlation scatter plot of miRNA expressions (BatchA).** (**A**). miRNA profiles of IFG patients do not correlate strongly to those of CTL at *R* = 0.78; in white circles while (**B**). the miRNA profiles of T2D patients showed a much weaker correlation to CTL at a further reduced *R* = 0.68; in white squares. The scatter plot of ∼100 detected miRNAs is drawn to provide a snap shot of the distribution and the correlation between the two variables. Points closely clustered to a straightline indicate a stronger correlation between the variables.The plot shows that IFG and CTL has stronger association than T2D and CTL. CTL,healthy controls; IFG,impaired fasting glucose; T2D,type 2 diabetes.
(PDF)

Table S6 **Table shows mRNA microarray fold change values of IFG or T2D vs healthy control.** The values were used to construct the Principal Component Analysis (PCA) plot as in Table S7. Biotin-labelled complementary RNA probes were synthesized with the Illumina TotalPrep RNA Amplification kit (Ambion). Gene profiling was performed with Sentrix BeadChip Array HumanRef-8 v2 (Illumina). Data were analysed with the BeadStudio software. Differentially expressed genes were selected based on the following parameters as suggested by the manufacturer: p value <0.05, Diff score >20, Average signal >100. All array data can be accessed from Gene Expression Omnibus (GEO) with accession no. GSE21321; SuperSeries GSE26168. CTL, healthy controls; IFG, impaired fasting glucose; T2D, type 2 diabetes.
(XLS)

Table S7 **PCA plot of mRNA profiles of IFG and T2D patients (Batch A).** Principal component analysis (PCA) plot based on mRNA expression showed a less distinguished classification of IFG and T2D patients. Batch A patients consists of six IFG patients (labeled as 1IFG to 6IFG) and eight T2D patients (labeled as 1T2D to 8T2D). IFG,impaired fasting glucose; T2D,type 2 diabetes.
(PDF)

Table S8 **Validation of microRNAs and mRNAs microarray.** The microarray data for the expression of the 8 “signature” miRNAs and their respective target mRNAs were validated using quantitative real-time PCR. Each miRNA/mRNA were assayed in triplicates for 3 separate experiments and the relative expression values are stated ± SEM. Fold change for the real-time PCR data was computed from the 2^−ΔΔCt^ values. Statistically significant differences are tested using Student's t-test at p<0.05 significance. CTL, healthy controls; IFG, impaired fasting glucose; T2D, type 2 diabetes.
(DOC)

Table S9 **Quantitative Real-time PCR results for Batch B individuals (** **Fig. 6A&B** **).** The 8 “signature” miRNAs (A) and their respective target mRNAs (B) were validated using quantitative real-time PCR in the Batch B blood samples. Each miRNA/mRNA were assayed in triplicates for 3 separate experiments and the relative expression values are stated ± SEM. Fold change for the real-time PCR data was computed from the 2^−ΔΔCt^ values. Statistically significant differences are tested using Student's t-test at p<0.05 significance. CTL, healthy controls; IFG, impaired fasting glucose; T2D, type 2 diabetes.
(DOC)

Table S10 **Expression of miRNAs and mRNAs in rat models.** (A) Endogenous expression levels of (abundance) of miRNAs and mRNAs in control tissues (adipose, pancreas, skeletal muscle and liver), blood and exosomes of rat model were assayed using quantitative real-time PCR analysis and expressed as delta threshold cycle (ΔCt) value with respect to 18S rRNA. Lower ΔCt value indicates higher expression levels. (B) Stem-loop RT-PCR results for T2D rat model induced by a combination of low dose STZ (40 mg/kg) and high-fat diet (Fig. 7A) (C) Quantitative Real-time PCR results for the 8 “signature” miRNA (B) respective target mRNA in T2D rat model (Fig. 7B). Expression values (B & C) are tabulated as fold change calculated as a ratio of T2D versus control, 2^−ΔΔCt^ ± SEM. Fold change below 1 are expressed as the negative reciprocal value. Validation for each miRNA/mRNA were assayed in triplicates for 3 separate experiments. T2D, type 2 diabetes.
(DOC)

Table S11 **Quantitative Real-time PCR miRNA (A) and mRNA (B) analysis for islet primary culture (** **Fig. 8A&B** **).** Expression values are tabulated as fold change calculated as a ratio of high glucose (25 mM) versus basal glucose control (5 mM) ratio of T2D versus control, 2^−ΔΔCt^ ± SEM. Fold change below 1 are expressed as the negative reciprocal value. Validation for each miRNA/mRNA was assayed in triplicates for 3 separate experiments.
(DOC)

Table S12 **Binding sites of miR-144 at 3′UTR of** ***IRS1*** **and mutated constructs of the binding sites.** 3′UTR of IRS1 contains two miR-144 binding sites (highlighted). Fragment with 1^st^ binding site of miR-144 alone includes 3′UTR of IRS1 from 3781-4201bp, while fragment with 2^nd^ binding site of miR144 alone includes 3′UTR of IRS1 from 4501-4921bp. Fragment with both binding sites of miR-144 includes 3′UTR of IRS1 from 3781-4921bp. Seed region if miR-144 is shown in *bold italics*. Mutations are marked with asterisks.
(DOC)
