# Correction: MicroRNA 144 Impairs Insulin Signaling by Inhibiting the Expression of Insulin Receptor Substrate 1 in Type 2 Diabetes Mellitus

**DOI:** 10.1371/annotation/698b7123-174f-4a09-95c9-fd6f5017d622

**Published:** 2011-09-02

**Authors:** Dwi Setyowati Karolina, Arunmozhiarasi Armugam, Subramaniam Tavintharan, Michael T. K. Wong, Su Chi Lim, Chee Fang Sum, Kandiah Jeyaseelan

The authors would like to state a misinterpretation of the findings of Professor Timmons and his coworkers in Reference 61 [Gallagher et al 2010. Genome Med. 2(2):9]. The authors wish to add: "A separate miRNA profiling study by Gallagher et al [61] using muscle tissue biopsies of impaired glucose tolerance (IGT) and T2D individuals showed 62 dysregulated miRNAs of which miR-144, miR-106b, miR-142-3p, miR-185, miR-451, miR-589, miR-665, miR-668, miR-126*, miR-199a-3p, miR-208a, miR-30e, miR-331-3p, miR-342-3p, miR-374a and miR-374b showed similar expression pattern as observed in our human/animal model studies. Moreover, miR-144 was also found to be highly up-regulated (with a fold change of 2.6) in their T2D samples." 

