# Supplementary material for: MicroRNA 144 Impairs Insulin Signaling by Inhibiting the Expression of Insulin Receptor Substrate 1 in Type 2 Diabetes Mellitus
Source: PLoS One. 2011 Aug 1;6(8):e22839. doi: 10.1371/journal.pone.0022839 (PMC3148231; doi:10.1371/journal.pone.0022839)
Supplement: Table S1 — MicroRNA microarray results of T2D rat model ( Fig. 2A ). Only miRNAs that are conserved in both human and rats, and with background subtracted mean signal intensities above 300 are included. Filtered signal intensities were normalized against U6snRNA. Values shown are fold changes calculated as a ratio of T2D versus control ± SEM. The grey boxes indicate negative expression values. Array data can be accessed from Gene Expression Omnibus (GEO) with accession no. GSE26167; SuperSeries GSE26168. T2D, type 2 diabetes. (DOC) [file pone.0022839.s001.doc]

**S1: MicroRNA microarray results of T2D rat model (Fig. 2A).** Only miRNAs that are conserved in both human and rats, and with background subtracted mean signal intensities above 300 are included. Filtered signal intensities were normalized against U6snRNA. Values shown are fold changes calculated as a ratio of T2D versus control ± SEM. The grey boxes indicate negative expression values. Array data can be accessed from Gene Expression Omnibus (GEO) with accession no. GSE26167; SuperSeries GSE26168 . T2D, type 2 diabetes.

|  | **Fold change±SEM (T2D/control)** | | | | | | | | | | | | | | | | | | | | | | | | | | | | | | | | | | | | | | | | | | | | | | | | | | | | | | | | | | | | | | | | | | | | | | | | | | | | | | | | | | | |
| --- | --- | --- | --- | --- | --- | --- | --- | --- | --- | --- | --- | --- | --- | --- | --- | --- | --- | --- | --- | --- | --- | --- | --- | --- | --- | --- | --- | --- | --- | --- | --- | --- | --- | --- | --- | --- | --- | --- | --- | --- | --- | --- | --- | --- | --- | --- | --- | --- | --- | --- | --- | --- | --- | --- | --- | --- | --- | --- | --- | --- | --- | --- | --- | --- | --- | --- | --- | --- | --- | --- | --- | --- | --- | --- | --- | --- | --- | --- | --- | --- | --- | --- | --- | --- |
| **Name** | **Rat blood** | | | | | | | | | | | | | | | | **Rat adipose** | | | | | | | | | | | | | | | | | | | **Rat pancreas** | | | | | | | | | | | | | | | | | | **Rat skeletal muscle** | | | | | | | | | | | | | | | | | **Rat liver** | | | | | | | | | | | | | |
| **rno-let-7b*** | 0.527 | | | ± | | | | | | 0.057 | | | | | | | 1.288 | | | | | | ± | | | | | | 0.268 | | | | | | | 0.799 | | | | | | | | | ± | | | | 0.287 | | | | | 1.249 | | | | | | | | ± | | | | 0.618 | | | | | 1.206 | | | | | | | | ± | | | 0.479 | | |
| **rno-let-7b** | 0.712 | | | ± | | | | | | 0.055 | | | | | | | 1.444 | | | | | | ± | | | | | | 0.080 | | | | | | | 1.670 | | | | | | | | | ± | | | | 0.097 | | | | | 0.933 | | | | | | | | ± | | | | 0.065 | | | | | 2.126 | | | | | | | | ± | | | 0.238 | | |
| **rno-let-7c** | 0.806 | | | ± | | | | | | 0.080 | | | | | | | 1.209 | | | | | | ± | | | | | | 0.011 | | | | | | | 1.325 | | | | | | | | | ± | | | | 0.052 | | | | | 0.858 | | | | | | | | ± | | | | 0.080 | | | | | 1.616 | | | | | | | | ± | | | 0.083 | | |
| **rno-let-7d*** | 0.690 | | | ± | | | | | | 0.048 | | | | | | | 1.408 | | | | | | ± | | | | | | 0.031 | | | | | | | 0.893 | | | | | | | | | ± | | | | 0.099 | | | | | 1.108 | | | | | | | | ± | | | | 0.023 | | | | | 1.780 | | | | | | | | ± | | | 0.219 | | |
| **rno-let-7d** | 1.594 | | | ± | | | | | | 0.106 | | | | | | | 1.286 | | | | | | ± | | | | | | 0.130 | | | | | | | 5.459 | | | | | | | | | ± | | | | 0.710 | | | | | 1.462 | | | | | | | | ± | | | | 0.066 | | | | | 1.469 | | | | | | | | ± | | | 0.022 | | |
| **rno-let-7e** | 1.794 | | | ± | | | | | | 0.047 | | | | | | | 1.049 | | | | | | ± | | | | | | 0.001 | | | | | | | 0.768 | | | | | | | | | ± | | | | 0.088 | | | | | 1.272 | | | | | | | | ± | | | | 0.071 | | | | | 0.896 | | | | | | | | ± | | | 0.020 | | |
| **rno-let-7f** | 0.986 | | | ± | | | | | | 0.071 | | | | | | | 1.005 | | | | | | ± | | | | | | 0.001 | | | | | | | 3.061 | | | | | | | | | ± | | | | 0.351 | | | | | 1.000 | | | | | | | | ± | | | | 0.001 | | | | | 1.386 | | | | | | | | ± | | | 0.069 | | |
| **rno-let-7i** | 0.574 | | | ± | | | | | | 0.061 | | | | | | | 1.390 | | | | | | ± | | | | | | 0.127 | | | | | | | 1.227 | | | | | | | | | ± | | | | 0.902 | | | | | 1.281 | | | | | | | | ± | | | | 0.054 | | | | | 1.726 | | | | | | | | ± | | | 0.117 | | |
| **rno-miR-100** |  | | |  | | | | | |  | | | | | | | 1.193 | | | | | | ± | | | | | | 0.106 | | | | | | | 1.729 | | | | | | | | | ± | | | | 0.336 | | | | | 1.493 | | | | | | | | ± | | | | 0.067 | | | | | 2.221 | | | | | | | | ± | | | 0.203 | | |
| **rno-miR-101a** | 0.626 | | | ± | | | | | | 0.069 | | | | | | | 0.561 | | | | | | ± | | | | | | 0.020 | | | | | | | 6.964 | | | | | | | | | ± | | | | 0.706 | | | | | 0.982 | | | | | | | | ± | | | | 0.014 | | | | | 2.214 | | | | | | | | ± | | | 0.130 | | |
| **rno-miR-103** | 0.709 | | | ± | | | | | | 0.059 | | | | | | | 1.380 | | | | | | ± | | | | | | 0.030 | | | | | | | 1.622 | | | | | | | | | ± | | | | 0.500 | | | | | 1.086 | | | | | | | | ± | | | | 0.031 | | | | | 2.449 | | | | | | | | ± | | | 0.280 | | |
| **rno-miR-106b*** | 0.718 | | | ± | | | | | | 0.021 | | | | | | | 0.646 | | | | | | ± | | | | | | 0.364 | | | | | | |  | | | | | | | | |  | | | |  | | | | | 0.823 | | | | | | | | ± | | | | 0.036 | | | | | 0.613 | | | | | | | | ± | | | 0.061 | | |
| **rno-miR-106b** | 1.107 | | | ± | | | | | | 0.002 | | | | | | | 1.828 | | | | | | ± | | | | | | 0.070 | | | | | | | 4.882 | | | | | | | | | ± | | | | 0.674 | | | | | 1.413 | | | | | | | | ± | | | | 0.085 | | | | | 1.438 | | | | | | | | ± | | | 0.215 | | |
| **rno-miR-10a-3p** |  | | |  | | | | | |  | | | | | | | 1.266 | | | | | | ± | | | | | | 0.039 | | | | | | | 0.500 | | | | | | | | | ± | | | | 0.146 | | | | | 0.738 | | | | | | | | ± | | | | 0.090 | | | | |  | | | | | | | |  | | |  | | |
| **rno-miR-10a-5p** |  | | |  | | | | | |  | | | | | | | 1.121 | | | | | | ± | | | | | | 0.056 | | | | | | |  | | | | | | | | |  | | | |  | | | | | 1.365 | | | | | | | | ± | | | | 0.319 | | | | | 1.758 | | | | | | | | ± | | | 0.131 | | |
| **rno-miR-10b** |  | | |  | | | | | |  | | | | | | | 1.754 | | | | | | ± | | | | | | 0.034 | | | | | | | 1.998 | | | | | | | | | ± | | | | 0.385 | | | | | 1.577 | | | | | | | | ± | | | | 0.065 | | | | |  | | | | | | | | ± | | |  | | |
| **rno-miR-122** | 0.250 | | | ± | | | | | | 0.010 | | | | | | | 0.773 | | | | | | ± | | | | | | 0.660 | | | | | | | 1.149 | | | | | | | | | ± | | | | 0.366 | | | | | 0.440 | | | | | | | | ± | | | | 0.065 | | | | | 1.012 | | | | | | | | ± | | | 0.003 | | |
| **rno-miR-124*** |  | | |  | | | | | |  | | | | | | |  | | | | | |  | | | | | |  | | | | | | | 18.856 | | | | | | | | | ± | | | | 0.260 | | | | |  | | | | | | | |  | | | |  | | | | |  | | | | | | | |  | | |  | | |
| **rno-miR-124** | 1.259 | | | ± | | | | | | 0.017 | | | | | | | 1.259 | | | | | | ± | | | | | | 0.017 | | | | | | | 1.323 | | | | | | | | | ± | | | | 0.211 | | | | | 1.057 | | | | | | | | ± | | | | 0.012 | | | | |  | | | | | | | |  | | |  | | |
| **rno-miR-125a-3p** | 0.712 | | | ± | | | | | | 0.007 | | | | | | | 0.712 | | | | | | ± | | | | | | 0.306 | | | | | | | 0.643 | | | | | | | | | ± | | | | 0.050 | | | | | 0.417 | | | | | | | | ± | | | | 0.095 | | | | |  | | | | | | | |  | | |  | | |
| **rno-miR-125a-5p** | 1.103 | | | ± | | | | | | 0.019 | | | | | | | 1.103 | | | | | | ± | | | | | | 0.002 | | | | | | | 1.318 | | | | | | | | | ± | | | | 0.254 | | | | | 1.410 | | | | | | | | ± | | | | 0.074 | | | | | 2.508 | | | | | | | | ± | | | 0.080 | | |
| **rno-miR-125b-5p** | 1.334 | | | ± | | | | | | 0.093 | | | | | | | 1.000 | | | | | | ± | | | | | | 0.002 | | | | | | | 2.446 | | | | | | | | | ± | | | | 0.182 | | | | | 0.989 | | | | | | | | ± | | | | 0.006 | | | | | 1.726 | | | | | | | | ± | | | 0.117 | | |
| **rno-miR-125b-3p** | 1.461 | | | ± | | | | | | 0.017 | | | | | | | 1.355 | | | | | | ± | | | | | | 0.026 | | | | | | | 0.680 | | | | | | | | | ± | | | | 0.028 | | | | | 2.932 | | | | | | | | ± | | | | 0.030 | | | | | 1.502 | | | | | | | | ± | | | 0.828 | | |
| **rno-miR-125b*** |  | | |  | | | | | |  | | | | | | | 1.173 | | | | | | ± | | | | | | 0.024 | | | | | | | 1.106 | | | | | | | | | ± | | | | 0.088 | | | | | 1.323 | | | | | | | | ± | | | | 0.130 | | | | | 1.906 | | | | | | | | ± | | | 0.077 | | |
| **rno-miR-126*** |  | | |  | | | | | |  | | | | | | | 1.945 | | | | | | ± | | | | | | 0.039 | | | | | | |  | | | | | | | | |  | | | |  | | | | | 1.482 | | | | | | | | ± | | | | 0.108 | | | | | 1.206 | | | | | | | | ± | | | 0.039 | | |
| **rno-miR-126** | 0.942 | | | ± | | | | | | 0.091 | | | | | | | 1.001 | | | | | | ± | | | | | | 0.001 | | | | | | | 7.835 | | | | | | | | | ± | | | | 0.750 | | | | | 1.001 | | | | | | | | ± | | | | 0.001 | | | | | 1.456 | | | | | | | | ± | | | 0.070 | | |
| **rno-miR-129** | 0.609 | | | ± | | | | | | 0.027 | | | | | | | 1.368 | | | | | | ± | | | | | | 0.108 | | | | | | | 0.660 | | | | | | | | | ± | | | | 0.090 | | | | | 1.693 | | | | | | | | ± | | | | 0.047 | | | | | 1.553 | | | | | | | | ± | | | 0.044 | | |
|  | **Fold change±SEM (T2D/control)** | | | | | | | | | | | | | | | | | | | | | | | | | | | | | | | | | | | | | | | | | | | | | | | | | | | | | | | | | | | | | | | | | | | | | | | | | | | | | | | | | | |  |
| **Name** | **Rat blood** | | | | | | | | | | | | | | **Rat adipose** | | | | | | | | | | | | | | | | | | | **Rat pancreas** | | | | | | | | | | | | | | | | | | **Rat skeletal muscle** | | | | | | | | | | | | | | | | | **Rat liver** | | | | | | | | | | | | | | |  |
| **rno-miR-130a** | 0.945 | | | | | ± | | | 0.075 | | | | | | 0.886 | | | | | | | ± | | | | | | 0.038 | | | | | | 1.565 | | | | | | ± | | | | | | 0.259 | | | | | | 0.937 | | | | | | ± | | | | | 0.066 | | | | | | 0.733 | | | | | ± | | | | | 0.028 | | | | |  |
| **rno-miR-130b** | 0.805 | | | | | ± | | | 0.072 | | | | | | 1.563 | | | | | | | ± | | | | | | 0.029 | | | | | | 0.680 | | | | | | ± | | | | | | 0.217 | | | | | | 1.007 | | | | | | ± | | | | | 0.039 | | | | | |  | | | | |  | | | | |  | | | | |  |
| **rno-miR-133a** |  | | | | |  | | |  | | | | | | 1.785 | | | | | | | ± | | | | | | 0.115 | | | | | | 2.515 | | | | | | ± | | | | | | 0.755 | | | | | | 1.002 | | | | | | ± | | | | | 0.001 | | | | | |  | | | | |  | | | | |  | | | | |  |
| **rno-miR-135b** |  | | | | |  | | |  | | | | | | 0.961 | | | | | | | ± | | | | | | 0.218 | | | | | | 0.842 | | | | | | ± | | | | | | 0.276 | | | | | | 0.550 | | | | | | ± | | | | | 0.036 | | | | | | 0.725 | | | | | ± | | | | | 0.118 | | | | |  |
| **rno-miR-136** |  | | | | |  | | |  | | | | | | 1.201 | | | | | | | ± | | | | | | 0.219 | | | | | | 1.341 | | | | | | ± | | | | | | 0.285 | | | | | | 0.380 | | | | | | ± | | | | | 0.001 | | | | | |  | | | | |  | | | | |  | | | | |  |
| **rno-miR-138** | 0.601 | | | | | ± | | | 0.180 | | | | | | 1.018 | | | | | | | ± | | | | | | 0.063 | | | | | | 0.622 | | | | | | ± | | | | | | 0.020 | | | | | | 1.085 | | | | | | ± | | | | | 0.040 | | | | | |  | | | | |  | | | | |  | | | | |  |
| **rno-miR-138*** | 0.823 | | | | | ± | | | 0.058 | | | | | | 1.324 | | | | | | | ± | | | | | | 0.055 | | | | | | 1.124 | | | | | | ± | | | | | | 0.331 | | | | | | 1.127 | | | | | | ± | | | | | 0.037 | | | | | | 1.461 | | | | | ± | | | | | 0.103 | | | | |  |
| **rno-miR-140*** | 0.826 | | | | | ± | | | 0.073 | | | | | | 0.717 | | | | | | | ± | | | | | | 0.101 | | | | | | 1.154 | | | | | | ± | | | | | | 0.879 | | | | | | 1.786 | | | | | | ± | | | | | 0.071 | | | | | | 1.945 | | | | | ± | | | | | 0.075 | | | | |  |
| **rno-miR-140** | 1.346 | | | | | ± | | | 0.033 | | | | | | 1.237 | | | | | | | ± | | | | | | 0.015 | | | | | | 1.579 | | | | | | ± | | | | | | 0.141 | | | | | | 0.981 | | | | | | ± | | | | | 0.034 | | | | | | 1.410 | | | | | ± | | | | | 0.054 | | | | |  |
| **rno-miR-142-3p** | 0.999 | | | | | ± | | | 0.003 | | | | | | 2.051 | | | | | | | ± | | | | | | 0.055 | | | | | | 4.153 | | | | | | ± | | | | | | 0.281 | | | | | | 0.946 | | | | | | ± | | | | | 0.009 | | | | | | 1.588 | | | | | ± | | | | | 0.066 | | | | |  |
| **rno-miR-142-5p** | 1.007 | | | | | ± | | | 0.003 | | | | | | 1.454 | | | | | | | ± | | | | | | 0.085 | | | | | | 2.596 | | | | | | ± | | | | | | 0.550 | | | | | | 1.296 | | | | | | ± | | | | | 0.054 | | | | | | 1.502 | | | | | ± | | | | | 0.094 | | | | |  |
| **rno-miR-143** | 2.087 | | | | | ± | | | 0.097 | | | | | | 1.438 | | | | | | | ± | | | | | | 0.001 | | | | | | 3.509 | | | | | | ± | | | | | | 0.236 | | | | | | 1.000 | | | | | | ± | | | | | 0.001 | | | | | | 1.472 | | | | | ± | | | | | 0.051 | | | | |  |
| **rno-miR-144** | 1.826 | | | | | ± | | | 0.001 | | | | | | 4.343 | | | | | | | ± | | | | | | 0.178 | | | | | | 7.942 | | | | | | ± | | | | | | 0.171 | | | | | | 1.765 | | | | | | ± | | | | | 0.067 | | | | | | 4.263 | | | | | ± | | | | | 0.174 | | | | |  |
| **rno-miR-145** |  | | | | |  | | |  | | | | | | 1.821 | | | | | | | ± | | | | | | 0.047 | | | | | | 1.825 | | | | | | ± | | | | | | 0.007 | | | | | | 1.242 | | | | | | ± | | | | | 0.019 | | | | | | 2.050 | | | | | ± | | | | | 0.098 | | | | |  |
| **rno-miR-146a** | 0.359 | | | | | ± | | | 0.085 | | | | | | 0.216 | | | | | | | ± | | | | | | 0.036 | | | | | | 0.769 | | | | | | ± | | | | | | 0.063 | | | | | | 0.605 | | | | | | ± | | | | | 0.030 | | | | | | 0.476 | | | | | ± | | | | | 0.017 | | | | |  |
| **rno-miR-146b** | 1.594 | | | | | ± | | | 0.123 | | | | | | 1.264 | | | | | | | ± | | | | | | 0.052 | | | | | |  | | | | | |  | | | | | |  | | | | | | 1.581 | | | | | | ± | | | | | 0.101 | | | | | | 1.776 | | | | | ± | | | | | 0.195 | | | | |  |
| **rno-miR-148b-3p** | 0.946 | | | | | ± | | | 0.086 | | | | | | 0.933 | | | | | | | ± | | | | | | 0.069 | | | | | | 0.973 | | | | | | ± | | | | | | 0.418 | | | | | | 1.121 | | | | | | ± | | | | | 0.044 | | | | | | 2.053 | | | | | ± | | | | | 0.057 | | | | |  |
| **rno-miR-150** | 1.641 | | | | | ± | | | 0.029 | | | | | | 3.213 | | | | | | | ± | | | | | | 0.197 | | | | | | 1.447 | | | | | | ± | | | | | | 0.115 | | | | | | 1.567 | | | | | | ± | | | | | 0.015 | | | | | | 2.068 | | | | | ± | | | | | 0.388 | | | | |  |
| **rno-miR-151** | 1.202 | | | | | ± | | | 0.036 | | | | | | 1.607 | | | | | | | ± | | | | | | 0.060 | | | | | |  | | | | | | ± | | | | | |  | | | | | | 1.293 | | | | | | ± | | | | | 0.055 | | | | | | 1.760 | | | | | ± | | | | | 0.302 | | | | |  |
| **rno-miR-152** |  | | | | |  | | |  | | | | | | 1.293 | | | | | | | ± | | | | | | 0.086 | | | | | | 2.890 | | | | | | ± | | | | | | 0.417 | | | | | | 1.088 | | | | | | ± | | | | | 0.058 | | | | | | 1.000 | | | | | ± | | | | | 0.069 | | | | |  |
| **rno-miR-15b** | 0.994 | | | | | ± | | | 0.000 | | | | | | 1.505 | | | | | | | ± | | | | | | 0.031 | | | | | | 1.657 | | | | | | ± | | | | | | 0.628 | | | | | | 1.277 | | | | | | ± | | | | | 0.042 | | | | | | 1.473 | | | | | ± | | | | | 0.087 | | | | |  |
| **rno-miR-16** | 0.999 | | | | | ± | | | 0.009 | | | | | | 1.016 | | | | | | | ± | | | | | | 0.002 | | | | | | 3.082 | | | | | | ± | | | | | | 0.923 | | | | | | 1.000 | | | | | | ± | | | | | 0.003 | | | | | | 1.391 | | | | | ± | | | | | 0.052 | | | | |  |
| **rno-miR-17-3p** | 0.829 | | | | | ± | | | 0.006 | | | | | | 1.803 | | | | | | | ± | | | | | | 0.076 | | | | | |  | | | | | |  | | | | | |  | | | | | | 0.954 | | | | | | ± | | | | | 0.148 | | | | | |  | | | | |  | | | | |  | | | | |  |
| **rno-miR-181a** | 0.696 | | | | | ± | | | 0.042 | | | | | | 1.217 | | | | | | | ± | | | | | | 0.013 | | | | | | 1.329 | | | | | | ± | | | | | | 0.079 | | | | | | 0.648 | | | | | | ± | | | | | 0.051 | | | | | | 2.403 | | | | | ± | | | | | 0.126 | | | | |  |
| **rno-miR-182** | 0.539 | | | | | ± | | | 0.042 | | | | | | 0.284 | | | | | | | ± | | | | | | 0.009 | | | | | | 0.794 | | | | | | ± | | | | | | 0.077 | | | | | | 0.237 | | | | | | ± | | | | | 0.548 | | | | | | 0.383 | | | | | ± | | | | | 0.010 | | | | |  |
| **rno-miR-183** | 1.152 | | | | | ± | | | 0.593 | | | | | | 1.442 | | | | | | | ± | | | | | | 0.055 | | | | | | 2.928 | | | | | | ± | | | | | | 0.122 | | | | | | 0.709 | | | | | | ± | | | | | 0.029 | | | | | |  | | | | |  | | | | |  | | | | |  |
| **rno-miR-184** | 0.678 | | | | | ± | | | 0.008 | | | | | | 1.260 | | | | | | | ± | | | | | | 0.033 | | | | | | 0.954 | | | | | | ± | | | | | | 0.037 | | | | | | 1.181 | | | | | | ± | | | | | 0.010 | | | | | | 3.606 | | | | | ± | | | | | 0.144 | | | | |  |
| **rno-miR-185** | 1.326 | | | | | ± | | | 0.117 | | | | | | 1.158 | | | | | | | ± | | | | | | 0.060 | | | | | | 1.286 | | | | | | ± | | | | | | 0.522 | | | | | | 1.524 | | | | | | ± | | | | | 0.144 | | | | | | 1.595 | | | | | ± | | | | | 0.096 | | | | |  |
| **rno-miR-186** | 0.738 | | | | | ± | | | 0.294 | | | | | | 1.368 | | | | | | | ± | | | | | | 0.073 | | | | | | 0.433 | | | | | | ± | | | | | | 0.042 | | | | | | 1.200 | | | | | | ± | | | | | 0.033 | | | | | | 1.622 | | | | | ± | | | | | 0.328 | | | | |  |
| **rno-miR-190** |  | | | | |  | | |  | | | | | | 1.262 | | | | | | | ± | | | | | | 0.028 | | | | | |  | | | | | |  | | | | | |  | | | | | | 0.876 | | | | | | ± | | | | | 0.100 | | | | | | 1.720 | | | | | ± | | | | | 0.095 | | | | |  |
| **rno-miR-191** | 0.967 | | | | | ± | | | 0.071 | | | | | | 1.353 | | | | | | | ± | | | | | | 0.046 | | | | | | 1.356 | | | | | | ± | | | | | | 0.287 | | | | | | 1.499 | | | | | | ± | | | | | 0.028 | | | | | | 1.889 | | | | | ± | | | | | 0.044 | | | | |  |
|  | **Fold change±SEM (T2D/control)** | | | | | | | | | | | | | | | | | | | | | | | | | | | | | | | | | | | | | | | | | | | | | | | | | | | | | | | | | | | | | | | | | | | | | | | | | | | | | | | | | |  | |
| **Name** | **Rat blood** | | | | | | | | | | | | | **Rat adipose** | | | | | | | | | | | | | | | | | | **Rat pancreas** | | | | | | | | | | | | | | | | | | **Rat skeletal muscle** | | | | | | | | | | | | | | | | | **Rat liver** | | | | | | | | | | | | | | | |  | |
| **rno-miR-192** | 1.848 | | | | | ± | | 0.126 | | | | | | 1.923 | | | | | | | ± | | | | | 0.084 | | | | | | 5.360 | | | | | | | ± | | | 0.783 | | | | | | | | 1.844 | | | | | | ± | | | 0.178 | | | | | | | | 1.591 | | | | | ± | | | | 0.038 | | | | | | |  | |
| **rno-miR-193** | 0.798 | | | | | ± | | 0.063 | | | | | | 1.166 | | | | | | | ± | | | | | 0.001 | | | | | | 1.512 | | | | | | | ± | | | 0.089 | | | | | | | | 1.016 | | | | | | ± | | | 0.051 | | | | | | | | 1.510 | | | | | ± | | | | 0.047 | | | | | | |  | |
| **rno-miR-194** | 1.095 | | | | | ± | | 0.427 | | | | | | 1.206 | | | | | | | ± | | | | | 0.144 | | | | | |  | | | | | | |  | | |  | | | | | | | | 1.052 | | | | | | ± | | | 0.062 | | | | | | | | 1.361 | | | | | ± | | | | 0.102 | | | | | | |  | |
| **rno-miR-195** | 0.426 | | | | | ± | | 0.063 | | | | | | 1.145 | | | | | | | ± | | | | | 0.002 | | | | | |  | | | | | | |  | | |  | | | | | | | | 0.869 | | | | | | ± | | | 0.036 | | | | | | | | 1.488 | | | | | ± | | | | 0.043 | | | | | | |  | |
| **rno-miR-196a*** |  | | | | |  | |  | | | | | | 1.731 | | | | | | | ± | | | | | 0.078 | | | | | | 0.959 | | | | | | | ± | | | 0.121 | | | | | | | | 0.960 | | | | | | ± | | | 0.117 | | | | | | | |  | | | | |  | | | |  | | | | | | |  | |
| **rno-miR-199a-3p** |  | | | | |  | |  | | | | | | 0.751 | | | | | | | ± | | | | | 0.039 | | | | | |  | | | | | | |  | | |  | | | | | | | | 0.686 | | | | | | ± | | | 0.005 | | | | | | | | 1.715 | | | | | ± | | | | 0.348 | | | | | | |  | |
| **rno-miR-19a** | 1.633 | | | | | ± | | 0.042 | | | | | | 1.816 | | | | | | | ± | | | | | 0.140 | | | | | | 2.082 | | | | | | | ± | | | 0.280 | | | | | | | | 1.376 | | | | | | ± | | | 0.032 | | | | | | | | 1.387 | | | | | ± | | | | 0.069 | | | | | | |  | |
| **rno-miR-19b** | 1.949 | | | | | ± | | 0.010 | | | | | | 2.002 | | | | | | | ± | | | | | 0.038 | | | | | | 3.543 | | | | | | | ± | | | 0.508 | | | | | | | | 1.302 | | | | | | ± | | | 0.035 | | | | | | | | 1.468 | | | | | ± | | | | 0.017 | | | | | | |  | |
| **rno-miR-200a** |  | | | | |  | |  | | | | | |  | | | | | | |  | | | | |  | | | | | | 3.603 | | | | | | | ± | | | 0.106 | | | | | | | |  | | | | | |  | | |  | | | | | | | | 1.310 | | | | | ± | | | | 0.127 | | | | | | |  | |
| **rno-miR-203** | 0.560 | | | | | ± | | 0.050 | | | | | | 0.443 | | | | | | | ± | | | | | 0.017 | | | | | | 0.623 | | | | | | | ± | | | 0.153 | | | | | | | | 0.709 | | | | | | ± | | | 0.646 | | | | | | | | 0.723 | | | | | ± | | | | 0.044 | | | | | | |  | |
| **rno-miR-204** | 1.325 | | | | | ± | | 0.329 | | | | | | 0.432 | | | | | | | ± | | | | | 0.353 | | | | | | 0.194 | | | | | | | ± | | | 0.072 | | | | | | | |  | | | | | |  | | |  | | | | | | | |  | | | | |  | | | |  | | | | | | |  | |
| **rno-miR-205** |  | | | | |  | |  | | | | | | 3.921 | | | | | | | ± | | | | | 0.025 | | | | | | 0.493 | | | | | | | ± | | | 0.117 | | | | | | | | 0.750 | | | | | | ± | | | 0.351 | | | | | | | |  | | | | |  | | | |  | | | | | | |  | |
| **rno-miR-206** | 0.929 | | | | | ± | | 0.065 | | | | | | 3.420 | | | | | | | ± | | | | | 0.016 | | | | | | 4.723 | | | | | | | ± | | | 0.232 | | | | | | | | 0.991 | | | | | | ± | | | 0.002 | | | | | | | | 2.420 | | | | | ± | | | | 0.345 | | | | | | |  | |
| **rno-miR-208** | 0.693 | | | | | ± | | 0.013 | | | | | | 1.018 | | | | | | | ± | | | | | 0.048 | | | | | | 0.637 | | | | | | | ± | | | 0.048 | | | | | | | | 0.799 | | | | | | ± | | | 0.029 | | | | | | | | 2.583 | | | | | ± | | | | 0.361 | | | | | | |  | |
| **rno-miR-21** | 1.869 | | | | | ± | | 0.041 | | | | | | 1.824 | | | | | | | ± | | | | | 0.004 | | | | | | 9.198 | | | | | | | ± | | | 0.777 | | | | | | | | 1.186 | | | | | | ± | | | 0.053 | | | | | | | | 1.916 | | | | | ± | | | | 0.003 | | | | | | |  | |
| **rno-miR-210** | 0.431 | | | | | ± | | 0.069 | | | | | | 2.227 | | | | | | | ± | | | | | 0.192 | | | | | |  | | | | | | |  | | |  | | | | | | | | 1.441 | | | | | | ± | | | 0.052 | | | | | | | |  | | | | |  | | | |  | | | | | | |  | |
| **rno-miR-212** |  | | | | |  | |  | | | | | | 1.266 | | | | | | | ± | | | | | 0.285 | | | | | | 0.682 | | | | | | | ± | | | 0.049 | | | | | | | | 1.003 | | | | | | ± | | | 0.147 | | | | | | | | 1.676 | | | | | ± | | | | 0.143 | | | | | | |  | |
| **rno-miR-214** | 0.844 | | | | | ± | | 0.114 | | | | | | 1.104 | | | | | | | ± | | | | | 0.023 | | | | | | 1.015 | | | | | | | ± | | | 0.080 | | | | | | | | 1.227 | | | | | | ± | | | 0.081 | | | | | | | | 2.636 | | | | | ± | | | | 0.029 | | | | | | |  | |
| **rno-miR-216a** |  | | | | |  | |  | | | | | |  | | | | | | |  | | | | |  | | | | | | 2.198 | | | | | | | ± | | | 0.079 | | | | | | | | 1.979 | | | | | | ± | | | 0.389 | | | | | | | |  | | | | |  | | | |  | | | | | | |  | |
| **rno-miR-22*** | 1.210 | | | | | ± | | 0.286 | | | | | | 1.261 | | | | | | | ± | | | | | 0.026 | | | | | | 1.843 | | | | | | | ± | | | 0.268 | | | | | | | | 1.446 | | | | | | ± | | | 0.035 | | | | | | | | 1.514 | | | | | ± | | | | 0.017 | | | | | | |  | |
| **rno-miR-22** | 0.780 | | | | | ± | | 0.118 | | | | | | 1.000 | | | | | | | ± | | | | | 0.001 | | | | | | 1.268 | | | | | | | ± | | | 0.093 | | | | | | | | 0.996 | | | | | | ± | | | 0.002 | | | | | | | | 1.015 | | | | | ± | | | | 0.002 | | | | | | |  | |
| **rno-miR-221** | 0.741 | | | | | ± | | 0.042 | | | | | | 0.821 | | | | | | | ± | | | | | 0.050 | | | | | | 3.450 | | | | | | | ± | | | 0.386 | | | | | | | | 0.868 | | | | | | ± | | | 0.078 | | | | | | | | 0.664 | | | | | ± | | | | 0.300 | | | | | | |  | |
| **rno-miR-222** | 0.672 | | | | | ± | | 0.034 | | | | | | 0.563 | | | | | | | ± | | | | | 0.064 | | | | | | 0.630 | | | | | | | ± | | | 0.265 | | | | | | | | 1.121 | | | | | | ± | | | 0.060 | | | | | | | | 2.012 | | | | | ± | | | | 0.075 | | | | | | |  | |
| **rno-miR-223** | 0.536 | | | | | ± | | 0.043 | | | | | | 1.095 | | | | | | | ± | | | | | 0.077 | | | | | | 0.424 | | | | | | | ± | | | 0.544 | | | | | | | | 2.656 | | | | | | ± | | | 0.145 | | | | | | | | 1.700 | | | | | ± | | | | 0.110 | | | | | | |  | |
| **rno-miR-23a*** |  | | | | |  | |  | | | | | | 1.042 | | | | | | | ± | | | | | 0.021 | | | | | | 1.231 | | | | | | | ± | | | 0.141 | | | | | | | | 0.907 | | | | | | ± | | | 0.034 | | | | | | | |  | | | | |  | | | |  | | | | | | |  | |
| **rno-miR-23a** | 0.598 | | | | | ± | | 0.062 | | | | | | 1.003 | | | | | | | ± | | | | | 0.001 | | | | | | 1.397 | | | | | | | ± | | | 0.074 | | | | | | | | 1.004 | | | | | | ± | | | 0.001 | | | | | | | | 2.433 | | | | | ± | | | | 0.056 | | | | | | |  | |
| **rno-miR-24** | 0.726 | | | | | ± | | 0.050 | | | | | | 1.018 | | | | | | | ± | | | | | 0.002 | | | | | | 0.823 | | | | | | | ± | | | 0.041 | | | | | | | | 0.999 | | | | | | ± | | | 0.005 | | | | | | | | 1.486 | | | | | ± | | | | 0.077 | | | | | | |  | |
| **rno-miR-24-1*** | 0.707 | | | | | ± | | 0.020 | | | | | | 1.494 | | | | | | | ± | | | | | 0.018 | | | | | | 1.472 | | | | | | | ± | | | 0.189 | | | | | | | | 1.291 | | | | | | ± | | | 0.053 | | | | | | | | 1.691 | | | | | ± | | | | 0.080 | | | | | | |  | |
| **rno-miR-24-2*** | 0.696 | | | | | ± | | 0.146 | | | | | | 1.209 | | | | | | | ± | | | | | 0.075 | | | | | |  | | | | | | |  | | |  | | | | | | | | 1.685 | | | | | | ± | | | 0.132 | | | | | | | |  | | | | |  | | | |  | | | | | | |  | |
| **rno-miR-25** | 0.558 | | | | | ± | | 0.020 | | | | | | 1.818 | | | | | | | ± | | | | | 0.176 | | | | | | 0.709 | | | | | | | ± | | | 0.100 | | | | | | | | 1.288 | | | | | | ± | | | 0.032 | | | | | | | | 1.499 | | | | | ± | | | | 0.057 | | | | | | |  | |
| **rno-miR-26a** | 0.976 | | | | | ± | | 0.009 | | | | | | 1.012 | | | | | | | ± | | | | | 0.001 | | | | | | 1.909 | | | | | | | ± | | | 0.077 | | | | | | | | 0.997 | | | | | | ± | | | 0.003 | | | | | | | | 1.872 | | | | | ± | | | | 0.071 | | | | | | |  | |
|  | | | **Fold change±SEM (T2D/control)** | | | | | | | | | | | | | | | | | | | | | | | | | | | | | | | | | | | | | | | | | | | | | | | | | | | | | | | | | | | | | | | | | | | | | | | | | | | | | | | | | |
| **Name** | | | **Rat blood** | | | | | | | | | | | | | | | **Rat adipose** | | | | | | | | | | | | | | | | | | | **Rat pancreas** | | | | | | | | | | | | | | | | **Rat skeletal muscle** | | | | | | | | | | | | | | | | | **Rat liver** | | | | | | | | | | | | | | |
| **rno-miR-26b** | | | 0.990 | | | ± | | | | | 0.051 | | | | | | | 1.003 | | | | | | | ± | | | | | | 0.002 | | | | | | 2.414 | | | | | | ± | | | | | 0.100 | | | | | 1.001 | | | | | | | | ± | | | | 0.001 | | | | | 1.426 | | | | | | | | ± | | | 0.045 | | | |
| **rno-miR-27a** | | | 1.431 | | | ± | | | | | 0.708 | | | | | | | 1.660 | | | | | | | ± | | | | | | 0.029 | | | | | | 1.784 | | | | | | ± | | | | | 0.020 | | | | | 1.359 | | | | | | | | ± | | | | 0.044 | | | | | 1.222 | | | | | | | | ± | | | 0.087 | | | |
| **rno-miR-27b** | | |  | | |  | | | | |  | | | | | | | 1.522 | | | | | | | ± | | | | | | 0.139 | | | | | | 3.872 | | | | | | ± | | | | | 0.295 | | | | | 1.213 | | | | | | | | ± | | | | 0.079 | | | | |  | | | | | | | |  | | |  | | | |
| **rno-miR-296** | | |  | | |  | | | | |  | | | | | | | 0.756 | | | | | | | ± | | | | | | 0.092 | | | | | | 2.400 | | | | | | ± | | | | | 0.471 | | | | | 0.890 | | | | | | | | ± | | | | 0.073 | | | | |  | | | | | | | |  | | |  | | | |
| **rno-miR-296*** | | | 0.537 | | | ± | | | | | 0.081 | | | | | | |  | | | | | | |  | | | | | |  | | | | | | 1.404 | | | | | | ± | | | | | 0.123 | | | | | 1.774 | | | | | | | | ± | | | | 0.147 | | | | | 2.297 | | | | | | | | ± | | | 0.114 | | | |
| **rno-miR-29a*** | | |  | | |  | | | | |  | | | | | | | 0.987 | | | | | | | ± | | | | | | 0.013 | | | | | | 0.031 | | | | | | ± | | | | | 0.073 | | | | | 1.260 | | | | | | | | ± | | | | 0.063 | | | | | 2.061 | | | | | | | | ± | | | 0.111 | | | |
| **rno-miR-29a** | | | 1.577 | | | ± | | | | | 0.020 | | | | | | | 2.559 | | | | | | | ± | | | | | | 0.067 | | | | | | 0.999 | | | | | | ± | | | | | 0.001 | | | | | 4.959 | | | | | | | | ± | | | | 0.179 | | | | | 2.359 | | | | | | | | ± | | | 0.044 | | | |
| **rno-miR-29b** | | | 0.712 | | | ± | | | | | 0.041 | | | | | | | 1.621 | | | | | | | ± | | | | | | 0.068 | | | | | | 1.610 | | | | | | ± | | | | | 0.033 | | | | | 2.890 | | | | | | | | ± | | | | 0.332 | | | | | 1.563 | | | | | | | | ± | | | 0.015 | | | |
| **rno-miR-29c*** | | |  | | |  | | | | |  | | | | | | | 1.736 | | | | | | | ± | | | | | | 0.101 | | | | | |  | | | | | |  | | | | |  | | | | | 0.992 | | | | | | | | ± | | | | 0.038 | | | | | 0.365 | | | | | | | | ± | | | 0.057 | | | |
| **rno-miR-29c** | | | 1.483 | | | ± | | | | | 0.143 | | | | | | | 1.707 | | | | | | | ± | | | | | | 0.053 | | | | | | 3.237 | | | | | | ± | | | | | 0.259 | | | | | 1.112 | | | | | | | | ± | | | | 0.022 | | | | | 2.285 | | | | | | | | ± | | | 0.051 | | | |
| **rno-miR-301a** | | | 0.623 | | | ± | | | | | 0.072 | | | | | | | 1.337 | | | | | | | ± | | | | | | 0.060 | | | | | |  | | | | | |  | | | | |  | | | | | 1.214 | | | | | | | | ± | | | | 0.099 | | | | | 1.507 | | | | | | | | ± | | | 0.080 | | | |
| **rno-miR-30a*** | | |  | | |  | | | | |  | | | | | | | 1.484 | | | | | | | ± | | | | | | 0.037 | | | | | |  | | | | | |  | | | | |  | | | | | 1.107 | | | | | | | | ± | | | | 0.035 | | | | |  | | | | | | | |  | | |  | | | |
| **rno-miR-30a** | | | 0.989 | | | ± | | | | | 0.052 | | | | | | | 0.999 | | | | | | | ± | | | | | | 0.001 | | | | | | 0.594 | | | | | | ± | | | | | 0.073 | | | | | 0.995 | | | | | | | | ± | | | | 0.005 | | | | | 0.510 | | | | | | | | ± | | | 0.008 | | | |
| **rno-miR-30b-5p** | | | 0.697 | | | ± | | | | | 0.061 | | | | | | | 1.008 | | | | | | | ± | | | | | | 0.003 | | | | | | 0.590 | | | | | | ± | | | | | 0.044 | | | | | 0.979 | | | | | | | | ± | | | | 0.036 | | | | | 0.788 | | | | | | | | ± | | | 0.030 | | | |
| **rno-miR-30c** | | | 0.793 | | | ± | | | | | 0.119 | | | | | | | 1.003 | | | | | | | ± | | | | | | 0.000 | | | | | | 0.643 | | | | | | ± | | | | | 0.031 | | | | | 0.999 | | | | | | | | ± | | | | 0.001 | | | | | 0.548 | | | | | | | | ± | | | 0.025 | | | |
| **rno-miR-30d** | | | 0.607 | | | ± | | | | | 0.048 | | | | | | | 0.649 | | | | | | | ± | | | | | | 0.011 | | | | | | 0.356 | | | | | | ± | | | | | 0.004 | | | | | 1.026 | | | | | | | | ± | | | | 0.015 | | | | | 0.482 | | | | | | | | ± | | | 0.006 | | | |
| **rno-miR-30e*** | | | 0.858 | | | ± | | | | | 0.377 | | | | | | |  | | | | | | |  | | | | | |  | | | | | | 0.980 | | | | | | ± | | | | | 0.258 | | | | | 1.221 | | | | | | | | ± | | | | 0.046 | | | | | 0.693 | | | | | | | | ± | | | 0.047 | | | |
| **rno-miR-30e** | | | 0.727 | | | ± | | | | | 0.049 | | | | | | | 0.786 | | | | | | | ± | | | | | | 0.021 | | | | | | 1.625 | | | | | | ± | | | | | 0.082 | | | | | 1.000 | | | | | | | | ± | | | | 0.001 | | | | | 0.710 | | | | | | | | ± | | | 0.037 | | | |
| **rno-miR-31** | | |  | | |  | | | | |  | | | | | | |  | | | | | | |  | | | | | |  | | | | | |  | | | | | |  | | | | |  | | | | |  | | | | | | | |  | | | |  | | | | | 1.385 | | | | | | | | ± | | | 0.082 | | | |
| **rno-miR-32** | | | 0.812 | | | ± | | | | | 0.056 | | | | | | | 2.111 | | | | | | | ± | | | | | | 0.190 | | | | | |  | | | | | |  | | | | |  | | | | | 1.111 | | | | | | | | ± | | | | 0.116 | | | | | 1.170 | | | | | | | | ± | | | 0.156 | | | |
| **rno-miR-320** | | | 1.855 | | | ± | | | | | 0.071 | | | | | | | 1.250 | | | | | | | ± | | | | | | 0.033 | | | | | | 1.832 | | | | | | ± | | | | | 0.054 | | | | | 1.473 | | | | | | | | ± | | | | 0.145 | | | | | 2.677 | | | | | | | | ± | | | 0.143 | | | |
| **rno-miR-323** | | |  | | |  | | | | |  | | | | | | | 0.791 | | | | | | | ± | | | | | | 0.149 | | | | | | 0.693 | | | | | | ± | | | | | 0.005 | | | | | 1.013 | | | | | | | | ± | | | | 0.007 | | | | |  | | | | | | | |  | | |  | | | |
| **rno-miR-326** | | | 1.321 | | | ± | | | | | 0.252 | | | | | | | 1.106 | | | | | | | ± | | | | | | 0.236 | | | | | | 1.222 | | | | | | ± | | | | | 0.259 | | | | | 1.275 | | | | | | | | ± | | | | 0.062 | | | | |  | | | | | | | |  | | |  | | | |
| **rno-miR-328** | | | 0.681 | | | ± | | | | | 0.024 | | | | | | | 1.099 | | | | | | | ± | | | | | | 0.049 | | | | | | 2.034 | | | | | | ± | | | | | 0.338 | | | | | 1.573 | | | | | | | | ± | | | | 0.048 | | | | |  | | | | | | | |  | | |  | | | |
| **rno-miR-330** | | | 2.330 | | | ± | | | | | 0.431 | | | | | | | 0.366 | | | | | | | ± | | | | | | 0.009 | | | | | | 2.180 | | | | | | ± | | | | | 0.156 | | | | |  | | | | | | | |  | | | |  | | | | |  | | | | | | | |  | | |  | | | |
| **rno-miR-331** | | | 0.799 | | | ± | | | | | 0.069 | | | | | | | 0.737 | | | | | | | ± | | | | | | 0.028 | | | | | | 2.087 | | | | | | ± | | | | | 0.221 | | | | | 0.994 | | | | | | | | ± | | | | 0.343 | | | | | 1.963 | | | | | | | | ± | | | 0.133 | | | |
| **rno-miR-335** | | |  | | |  | | | | |  | | | | | | | 1.766 | | | | | | | ± | | | | | | 0.090 | | | | | | 2.584 | | | | | | ± | | | | | 0.484 | | | | | 1.008 | | | | | | | | ± | | | | 0.070 | | | | | 1.010 | | | | | | | | ± | | | 0.168 | | | |
| **rno-miR-338** | | |  | | |  | | | | |  | | | | | | | 1.048 | | | | | | | ± | | | | | | 0.079 | | | | | | 1.369 | | | | | | ± | | | | | 0.145 | | | | | 1.322 | | | | | | | | ± | | | | 0.136 | | | | | 1.662 | | | | | | | | ± | | | 0.467 | | | |
| **rno-miR-338*** | | |  | | |  | | | | |  | | | | | | | 0.721 | | | | | | | ± | | | | | | 0.053 | | | | | | 1.622 | | | | | | ± | | | | | 0.305 | | | | | 0.438 | | | | | | | | ± | | | | 0.017 | | | | |  | | | | | | | |  | | |  | | | |
| **rno-miR-339-3p** | | |  | | |  | | | | |  | | | | | | | 1.241 | | | | | | | ± | | | | | | 0.135 | | | | | |  | | | | | |  | | | | |  | | | | |  | | | | | | | |  | | | |  | | | | |  | | | | | | | |  | | |  | | | |
| **rno-miR-339-5p** | | | 1.405 | | | ± | | | | | 0.057 | | | | | | | 1.413 | | | | | | | ± | | | | | | 0.128 | | | | | | 1.941 | | | | | | ± | | | | | 0.477 | | | | | 1.427 | | | | | | | | ± | | | | 0.174 | | | | | 1.314 | | | | | | | | ± | | | 0.023 | | | |
|  | | | **Fold change±SEM (T2D/control)** | | | | | | | | | | | | | | | | | | | | | | | | | | | | | | | | | | | | | | | | | | | | | | | | | | | | | | | | | | | | | | | | | | | | | | | | | | | | | | | | | |
| **Name** | | | **Rat blood** | | | | | | | | | | | | | **Rat adipose** | | | | | | | | | | | | | | | | | | | **Rat pancreas** | | | | | | | | | | | | | | | | | **Rat skeletal muscle** | | | | | | | | | | | | | | | | | **Rat liver** | | | | | | | | | | | | | | | |
| **rno-miR-340-3p** | | 0.925 | | | ± | | | | | | | 0.134 | | | | | | | 1.217 | | | | | ± | | | | | | 0.215 | | | 1.715 | | | | | | | | ± | | | | | | 0.177 | | | | 0.843 | | | | | | ± | | | | | | | 0.013 | | | | 2.578 | | | | | | | ± | | | | | 0.506 | | | | |
| **rno-miR-342-3p** | | 0.653 | | | ± | | | | | | | 0.076 | | | | | | | 1.893 | | | | | ± | | | | | | 0.185 | | |  | | | | | | | |  | | | | | |  | | | | 1.112 | | | | | | ± | | | | | | | 0.063 | | | |  | | | | | | |  | | | | |  | | | | |
| **rno-miR-34a** | | 2.109 | | | ± | | | | | | | 0.198 | | | | | | | 1.249 | | | | | ± | | | | | | 0.037 | | | 2.475 | | | | | | | | ± | | | | | | 0.360 | | | | 1.542 | | | | | | ± | | | | | | | 0.205 | | | | 7.502 | | | | | | | ± | | | | | 0.157 | | | | |
| **rno-miR-361** | | 0.822 | | | ± | | | | | | | 0.110 | | | | | | | 1.098 | | | | | ± | | | | | | 0.095 | | | 0.871 | | | | | | | | ± | | | | | | 0.163 | | | | 0.788 | | | | | | ± | | | | | | | 0.752 | | | | 0.593 | | | | | | | ± | | | | | 0.028 | | | | |
| **rno-miR-363*** | | 0.542 | | | ± | | | | | | | 0.105 | | | | | | | 0.547 | | | | | ± | | | | | | 0.043 | | | 1.835 | | | | | | | | ± | | | | | | 0.214 | | | | 0.801 | | | | | | ± | | | | | | | 0.038 | | | | 1.509 | | | | | | | ± | | | | | 0.108 | | | | |
| **rno-miR-365** | | 0.749 | | | ± | | | | | | | 0.055 | | | | | | | 1.292 | | | | | ± | | | | | | 0.040 | | | 1.253 | | | | | | | | ± | | | | | | 0.645 | | | | 1.288 | | | | | | ± | | | | | | | 0.057 | | | | 1.495 | | | | | | | ± | | | | | 0.386 | | | | |
| **rno-miR-370** | | 0.989 | | | ± | | | | | | | 0.133 | | | | | | | 1.052 | | | | | ± | | | | | | 0.013 | | | 0.300 | | | | | | | | ± | | | | | | 0.013 | | | | 1.036 | | | | | | ± | | | | | | | 0.232 | | | | 0.990 | | | | | | | ± | | | | | 0.553 | | | | |
| **rno-miR-374** | | 2.038 | | | ± | | | | | | | 0.279 | | | | | | | 1.837 | | | | | ± | | | | | | 0.075 | | | 1.582 | | | | | | | | ± | | | | | | 0.184 | | | | 1.267 | | | | | | ± | | | | | | | 0.085 | | | | 2.147 | | | | | | | ± | | | | | 0.141 | | | | |
| **rno-miR-375** | | 0.846 | | | ± | | | | | | | 0.115 | | | | | | | 1.274 | | | | | ± | | | | | | 0.041 | | | 2.104 | | | | | | | | ± | | | | | | 0.098 | | | | 0.691 | | | | | | ± | | | | | | | 0.028 | | | | 2.184 | | | | | | | ± | | | | | 0.273 | | | | |
| **rno-miR-377** | |  | | |  | | | | | | |  | | | | | | | 2.539 | | | | | ± | | | | | | 0.106 | | | 3.051 | | | | | | | | ± | | | | | | 0.571 | | | | 0.906 | | | | | | ± | | | | | | | 0.038 | | | |  | | | | | | |  | | | | |  | | | | |
| **rno-miR-378*** | |  | | |  | | | | | | |  | | | | | | | 1.449 | | | | | ± | | | | | | 0.061 | | |  | | | | | | | |  | | | | | |  | | | | 1.092 | | | | | | ± | | | | | | | 0.757 | | | |  | | | | | | |  | | | | |  | | | | |
| **rno-miR-378** | | 0.690 | | | ± | | | | | | | 0.030 | | | | | | | 1.027 | | | | | ± | | | | | | 0.003 | | | 2.271 | | | | | | | | ± | | | | | | 0.134 | | | | 0.997 | | | | | | ± | | | | | | | 0.002 | | | | 1.948 | | | | | | | ± | | | | | 0.076 | | | | |
| **rno-miR-381** | | 0.625 | | | ± | | | | | | | 0.074 | | | | | | | 1.090 | | | | | ± | | | | | | 0.020 | | | 0.559 | | | | | | | | ± | | | | | | 0.037 | | | | 1.260 | | | | | | ± | | | | | | | 0.054 | | | | 1.990 | | | | | | | ± | | | | | 0.148 | | | | |
| **rno-miR-382** | | 3.288 | | | ± | | | | | | | 0.087 | | | | | | | 1.622 | | | | | ± | | | | | | 0.114 | | | 1.130 | | | | | | | | ± | | | | | | 0.150 | | | | 1.108 | | | | | | ± | | | | | | | 0.054 | | | | 1.738 | | | | | | | ± | | | | | 0.483 | | | | |
| **rno-miR-412** | |  | | |  | | | | | | |  | | | | | | | 0.934 | | | | | ± | | | | | | 0.138 | | | 0.737 | | | | | | | | ± | | | | | | 0.340 | | | | 0.676 | | | | | | ± | | | | | | | 0.026 | | | | 1.095 | | | | | | | ± | | | | | 0.049 | | | | |
| **rno-miR-423** | | 0.831 | | | ± | | | | | | | 0.052 | | | | | | | 0.638 | | | | | ± | | | | | | 0.016 | | | 0.784 | | | | | | | | ± | | | | | | 0.028 | | | | 1.209 | | | | | | ± | | | | | | | 0.031 | | | | 1.759 | | | | | | | ± | | | | | 0.071 | | | | |
| **rno-miR-425** | | 0.610 | | | ± | | | | | | | 0.036 | | | | | | | 0.568 | | | | | ± | | | | | | 0.047 | | |  | | | | | | | |  | | | | | |  | | | | 0.900 | | | | | | ± | | | | | | | 0.032 | | | |  | | | | | | |  | | | | |  | | | | |
| **rno-miR-451** | | 1.454 | | | ± | | | | | | | 0.000 | | | | | | | 2.864 | | | | | ± | | | | | | 0.053 | | |  | | | | | | | |  | | | | | |  | | | | 1.609 | | | | | | ± | | | | | | | 0.001 | | | | 1.750 | | | | | | | ± | | | | | 0.084 | | | | |
| **rno-miR-455** | |  | | |  | | | | | | |  | | | | | | |  | | | | |  | | | | | |  | | | 1.353 | | | | | | | | ± | | | | | | 0.169 | | | | 1.080 | | | | | | ± | | | | | | | 0.120 | | | | 0.949 | | | | | | | ± | | | | | 0.135 | | | | |
| **rno-miR-487b** | | 0.556 | | | ± | | | | | | | 0.039 | | | | | | | 0.964 | | | | | ± | | | | | | 0.049 | | | 0.823 | | | | | | | | ± | | | | | | 0.074 | | | | 0.624 | | | | | | ± | | | | | | | 0.020 | | | | 0.685 | | | | | | | ± | | | | | 0.018 | | | | |
| **rno-miR-494** | | 0.816 | | | ± | | | | | | | 0.024 | | | | | | | 1.098 | | | | | ± | | | | | | 0.013 | | | 0.981 | | | | | | | | ± | | | | | | 0.028 | | | | 1.391 | | | | | | ± | | | | | | | 0.082 | | | | 1.780 | | | | | | | ± | | | | | 0.086 | | | | |
| **rno-miR-497** | |  | | |  | | | | | | |  | | | | | | | 1.550 | | | | | ± | | | | | | 0.062 | | |  | | | | | | | |  | | | | | |  | | | | 1.134 | | | | | | ± | | | | | | | 0.042 | | | | 0.585 | | | | | | | ± | | | | | 0.878 | | | | |
| **rno-miR-499** | |  | | |  | | | | | | |  | | | | | | | 1.905 | | | | | ± | | | | | | 0.077 | | |  | | | | | | | |  | | | | | |  | | | | 3.421 | | | | | | ± | | | | | | | 0.102 | | | | 1.165 | | | | | | | ± | | | | | 0.134 | | | | |
| **rno-miR-505** | | 0.578 | | | ± | | | | | | | 0.057 | | | | | | | 1.179 | | | | | ± | | | | | | 0.061 | | | 0.734 | | | | | | | | ± | | | | | | 0.006 | | | | 0.860 | | | | | | ± | | | | | | | 0.058 | | | | 3.040 | | | | | | | ± | | | | | 0.127 | | | | |
| **rno-miR-532-5p** | |  | | |  | | | | | | |  | | | | | | | 0.727 | | | | | ± | | | | | | 0.035 | | | 1.250 | | | | | | | | ± | | | | | | 0.039 | | | | 0.891 | | | | | | ± | | | | | | | 0.025 | | | | 1.719 | | | | | | | ± | | | | | 0.367 | | | | |
| **rno-miR-542-3p** | | 0.684 | | | ± | | | | | | | 0.047 | | | | | | | 1.407 | | | | | ± | | | | | | 0.183 | | | 2.345 | | | | | | | | ± | | | | | | 0.301 | | | | 0.846 | | | | | | ± | | | | | | | 0.019 | | | | 2.977 | | | | | | | ± | | | | | 0.356 | | | | |
| **rno-miR-542-5p** | |  | | |  | | | | | | |  | | | | | | | 1.725 | | | | | ± | | | | | | 0.090 | | | 0.466 | | | | | | | | ± | | | | | | 0.032 | | | | 1.270 | | | | | | ± | | | | | | | 0.039 | | | |  | | | | | | |  | | | | |  | | | | |
| **rno-miR-551b** | | 0.736 | | | ± | | | | | | | 0.041 | | | | | | | 1.012 | | | | | ± | | | | | | 0.025 | | | 1.845 | | | | | | | | ± | | | | | | 0.152 | | | | 1.058 | | | | | | ± | | | | | | | 0.033 | | | | 1.540 | | | | | | | ± | | | | | 0.024 | | | | |
| **rno-miR-652** | | 0.749 | | | ± | | | | | | | 0.029 | | | | | | | 1.572 | | | | | ± | | | | | | 0.074 | | |  | | | | | | | |  | | | | | |  | | | | 0.921 | | | | | | ± | | | | | | | 0.451 | | | |  | | | | | | |  | | | | |  | | | | |
| **rno-miR-7a** | |  | | |  | | | | | | |  | | | | | | | 1.318 | | | | | ± | | | | | | 0.071 | | | 1.106 | | | | | | | | ± | | | | | | 0.280 | | | | 1.879 | | | | | | ± | | | | | | | 0.067 | | | | 2.136 | | | | | | | ± | | | | | 0.308 | | | | |
| **rno-miR-874** | |  | | |  | | | | | | |  | | | | | | | 0.509 | | | | | ± | | | | | | 0.028 | | | 0.798 | | | | | | | | ± | | | | | | 0.102 | | | | 1.330 | | | | | | ± | | | | | | | 0.165 | | | | 5.129 | | | | | | | ± | | | | | 0.990 | | | | |
|  | | **Fold change±SEM (T2D/control)** | | | | | | | | | | | | | | | | | | | | | | | | | | | | | | | | | | | | | | | | | | | | | | | | | | | | | | | | | | | | | | | | | | | | | | | | | | | | | | | | | | |
| **Name** | | **Rat blood** | | | | | | | | | | | **Rat adipose** | | | | | | | | | | | | | | | | | | | | **Rat pancreas** | | | | | | | | | | | | | | | | | | **Rat skeletal muscle** | | | | | | | | | | | | | | | | | **Rat liver** | | | | | | | | | | | | | | | | |
| **rno-miR-877** | | 0.952 | | | ± | | 0.231 | | | | | | 1.394 | | | | | | | ± | | | | | | | 0.035 | | | | | | 0.570 | | | | | ± | | | | | | 0.095 | | | | | | | 1.797 | | | | ± | | | | | 0.053 | | | | | | | | 2.351 | | | | | ± | | | | 0.075 | | | | | | | |
| **rno-miR-9** | | 1.801 | | | ± | | 0.564 | | | | | | 2.072 | | | | | | | ± | | | | | | | 0.198 | | | | | | 1.363 | | | | | ± | | | | | | 0.489 | | | | | | | 1.007 | | | | ± | | | | | 0.079 | | | | | | | | 1.250 | | | | | ± | | | | 0.224 | | | | | | | |
| **rno-miR-92a** | | 0.556 | | | ± | | 0.049 | | | | | |  | | | | | | |  | | | | | | |  | | | | | | 0.687 | | | | | ± | | | | | | 0.519 | | | | | | |  | | | |  | | | | |  | | | | | | | | 1.192 | | | | | ± | | | | 0.078 | | | | | | | |
| **rno-miR-92b** | | 1.839 | | | ± | | 0.054 | | | | | | 1.569 | | | | | | | ± | | | | | | | 0.102 | | | | | | 1.596 | | | | | ± | | | | | | 0.232 | | | | | | | 0.951 | | | | ± | | | | | 0.075 | | | | | | | | 3.556 | | | | | ± | | | | 0.622 | | | | | | | |
| **rno-miR-93** | | 0.936 | | | ± | | 0.048 | | | | | |  | | | | | | |  | | | | | | |  | | | | | | 0.884 | | | | | ± | | | | | | 0.164 | | | | | | | 1.269 | | | | ± | | | | | 0.091 | | | | | | | | 1.951 | | | | | ± | | | | 0.130 | | | | | | | |
| **rno-miR-98** | | 0.675 | | | ± | | 0.073 | | | | | | 1.282 | | | | | | | ± | | | | | | | 0.116 | | | | | | 3.282 | | | | | ± | | | | | | 0.049 | | | | | | | 1.286 | | | | ± | | | | | 0.095 | | | | | | | | 1.837 | | | | | ± | | | | 0.539 | | | | | | | |
| **rno-miR-99a** | |  | | |  | |  | | | | | | 1.220 | | | | | | | ± | | | | | | | 0.113 | | | | | | 1.949 | | | | | ± | | | | | | 0.270 | | | | | | | 1.845 | | | | ± | | | | | 0.132 | | | | | | | | 1.392 | | | | | ± | | | | 0.104 | | | | | | | |
| **rno-miR-99b*** | | 0.346 | | | ± | | 0.009 | | | | | | 1.396 | | | | | | | ± | | | | | | | 0.024 | | | | | | 0.717 | | | | | ± | | | | | | 0.062 | | | | | | | 0.959 | | | | ± | | | | | 0.010 | | | | | | | | 1.144 | | | | | ± | | | | 0.148 | | | | | | | |
| **rno-miR-99b** | |  | | |  | |  | | | | | | 0.926 | | | | | | | ± | | | | | | | 0.084 | | | | | | 0.975 | | | | | ± | | | | | | 0.113 | | | | | | | 1.224 | | | | ± | | | | | 0.046 | | | | | | | | 2.535 | | | | | ± | | | | 0.295 | | | | | | | |
