# Supplementary material for: MicroRNA 144 Impairs Insulin Signaling by Inhibiting the Expression of Insulin Receptor Substrate 1 in Type 2 Diabetes Mellitus
Source: PLoS One. 2011 Aug 1;6(8):e22839. doi: 10.1371/journal.pone.0022839 (PMC3148231; doi:10.1371/journal.pone.0022839)
Supplement: Table S2 — microRNAs expression in rat blood, liver, pancreas, skeletal muscle and adipose tissues. miRNAs that were filtered using the statistical analysis as described in the “Methods section” were further subjected to fold change analysis. Statistically significant differences are tested using Student's t-test at p<0.05 significance Fold change values are calculated as a ratio of T2D versus respective control ± SEM. Fold change values below 1 are expressed in negative values. miRNAs that exhibited at least±1.5 fold change have been included. miRNAs that are highly up/down regulated in all sources are marked in bold. Only miRNAs conserved in both humans and rats are shown. T2D, type 2 diabetes. (DOC) [file pone.0022839.s002.doc]

**S2: microRNAs expression in rat blood, adipose tissues, pancreas, skeletal muscle and liver.** miRNAs that were filtered using the statistical analysis as described in the “Methods section” were further subjected to fold change analysis. Statistically significant differences are tested using Student’s t-test at p<0.05 significance Fold change values are calculated as a ratio of T2D versus respective control ± SEM. Fold change values below 1 are expressed in negative values. miRNAs that exhibited at least ± 1.5 fold change have been included. miRNAs that are highly up/down regulated in all sources are marked in bold. Only miRNAs conserved in both humans and rats are shown. T2D, type 2 diabetes.

| **Rat blood** | **fold change ± SEM** | | | **p-value** |
| --- | --- | --- | --- | --- |
| rno-miR-122 | -4.005 | ± | 0.010 | 0.022 |
| ***rno-miR-146a*** | -2.785 | ± | 0.085 | 0.013 |
| rno-miR-195 | -2.350 | ± | 0.063 | 0.004 |
| rno-miR-210 | -2.318 | ± | 0.069 | 0.007 |
| rno-let-7b* | -1.898 | ± | 0.057 | 0.020 |
| rno-miR-223 | -1.865 | ± | 0.043 | 0.001 |
| ***rno-miR-182*** | -1.856 | ± | 0.042 | 0.005 |
| rno-miR-487b | -1.799 | ± | 0.039 | 0.003 |
| rno-miR-92a | -1.797 | ± | 0.049 | 0.002 |
| rno-miR-25 | -1.791 | ± | 0.020 | 0.000 |
| rno-miR-505 | -1.729 | ± | 0.057 | 0.006 |
| rno-miR-23a | -1.671 | ± | 0.062 | 0.000 |
| ***rno-miR-30d*** | -1.648 | ± | 0.048 | 0.001 |
| rno-miR-129 | -1.641 | ± | 0.027 | 0.001 |
| rno-miR-425 | -1.638 | ± | 0.036 | 0.000 |
| rno-miR-301a | -1.605 | ± | 0.072 | 0.003 |
| rno-miR-381 | -1.601 | ± | 0.074 | 0.046 |
| rno-miR-101a | -1.599 | ± | 0.069 | 0.019 |
| rno-miR-342-3p | -1.531 | ± | 0.076 | 0.023 |
| ***rno-miR-29a*** | 1.577 | ± | 0.020 | 0.001 |
| rno-miR-146b | 1.594 | ± | 0.123 | 0.001 |
| rno-let-7d | 1.594 | ± | 0.106 | 0.004 |
| rno-miR-19a | 1.633 | ± | 0.042 | 0.001 |
| ***rno-miR-150*** | 1.641 | ± | 0.029 | 0.016 |
| rno-let-7e | 1.794 | ± | 0.047 | 0.001 |
| rno-miR-9 | 1.801 | ± | 0.564 | 0.032 |
| ***rno-miR-144*** | 1.826 | ± | 0.001 | 0.000 |
| ***rno-miR-192*** | 1.848 | ± | 0.126 | 0.014 |
| ***rno-miR-320*** | 1.855 | ± | 0.071 | 0.000 |
| rno-miR-21 | 1.869 | ± | 0.041 | 0.000 |
| rno-miR-19b | 1.949 | ± | 0.010 | 0.000 |
| rno-miR-143 | 2.087 | ± | 0.097 | 0.002 |
| rno-miR-34a | 2.109 | ± | 0.198 | 0.037 |

| **Rat adipose** | **fold change ± SEM** | | | | | **p-value** |
| --- | --- | --- | --- | --- | --- | --- |
| ***rno-miR-146a*** | -4.619 | | ± | | 0.036 | 0.002 |
| ***rno-miR-182*** | -3.522 | | ± | | 0.009 | 0.001 |
| rno-miR-203 | -2.257 | | ± | | 0.017 | 0.001 |
| rno-miR-101a | -1.783 | | ± | | 0.020 | 0.001 |
| rno-miR-222 | -1.777 | | ± | | 0.064 | 0.007 |
| rno-miR-331 | -1.357 | | ± | | 0.028 | 0.018 |
| ***rno-miR-30d*** | -1.540 | | ± | | 0.011 | 0.000 |
| rno-miR-15b | 1.505 | | ± | | 0.031 | 0.001 |
| rno-miR-27b | 1.522 | | ± | | 0.139 | 0.003 |
| rno-miR-497 | 1.550 | | ± | | 0.062 | 0.001 |
| rno-miR-652 | 1.572 | | ± | | 0.074 | 0.003 |
| rno-miR-151 | 1.607 | | ± | | 0.060 | 0.012 |
| rno-miR-29b | 1.621 | | ± | | 0.068 | 0.001 |
| rno-miR-27a | 1.660 | | ± | | 0.029 | 0.015 |
| rno-miR-29c | 1.707 | | ± | | 0.053 | 0.001 |
| rno-miR-542-5p | 1.725 | | ± | | 0.090 | 0.004 |
| rno-miR-196a* | 1.731 | | ± | | 0.078 | 0.008 |
| rno-miR-10b | 1.754 | | ± | | 0.034 | 0.000 |
| rno-miR-335 | 1.766 | | ± | | 0.090 | 0.000 |
| rno-miR-133a | 1.785 | | ± | | 0.115 | 0.008 |
| rno-miR-17-3p | 1.803 | | ± | | 0.076 | 0.014 |
| rno-miR-25 | 1.818 | ± | | 0.176 | | 0.011 |
| rno-miR-145 | 1.821 | ± | | 0.047 | | 0.000 |
| rno-miR-21 | 1.824 | ± | | 0.004 | | 0.000 |
| rno-miR-106b | 1.828 | ± | | 0.070 | | 0.000 |
| rno-miR-374 | 1.837 | ± | | 0.075 | | 0.002 |
| rno-miR-499 | 1.905 | ± | | 0.077 | | 0.001 |
| ***rno-miR-192*** | 1.923 | ± | | 0.084 | | 0.001 |
| rno-miR-126* | 1.945 | ± | | 0.039 | | 0.000 |
| rno-miR-19b | 2.002 | ± | | 0.038 | | 0.000 |
| rno-miR-142-3p | 2.051 | ± | | 0.055 | | 0.000 |
| rno-miR-9 | 2.072 | ± | | 0.198 | | 0.049 |
| rno-miR-32 | 2.111 | ± | | 0.190 | | 0.009 |
| rno-miR-377 | 2.539 | ± | | 0.106 | | 0.004 |
| ***rno-miR-29a*** | 2.559 | ± | | 0.067 | | 0.000 |
| rno-miR-451 | 2.864 | ± | | 0.053 | | 0.000 |
| ***rno-miR-150*** | 3.213 | ± | | 0.197 | | 0.002 |
| rno-miR-205 | 3.921 | ± | | 0.025 | | 0.000 |
| ***rno-miR-144*** | 4.343 | ± | | 0.178 | | 0.000 |

| **Rat pancreas** | **fold change ± SEM** | | | **p-value** |
| --- | --- | --- | --- | --- |
| ***rno-miR-30d*** | ***-2.811*** | ± | 0.004 | 0.023 |
| rno-miR-542-5p | -2.147 | ± | 0.032 | 0.003 |
| rno-miR-381 | -1.788 | ± | 0.037 | 0.009 |
| rno-miR-877 | -1.755 | ± | 0.095 | 0.025 |
| rno-miR-30b-5p | -1.695 | ± | 0.044 | 0.002 |
| rno-miR-30a | -1.684 | ± | 0.073 | 0.047 |
| rno-miR-208 | -1.570 | ± | 0.048 | 0.006 |
| rno-miR-30c | -1.554 | ± | 0.031 | 0.002 |
| rno-miR-125a-3p | -1.554 | ± | 0.050 | 0.015 |
| rno-miR-125b-3p | -1.471 | ± | 0.028 | 0.001 |
| ***rno-miR-146a*** | ***-1.313*** | ± | 0.063 | 0.003 |
| ***rno-miR-182*** | ***-1.259*** | ± | 0.077 | 0.002 |
| rno-miR-19a | 2.082 | ± | 0.280 | 0.029 |
| rno-miR-375 | 2.104 | ± | 0.098 | 0.001 |
| rno-miR-216a | 2.198 | ± | 0.079 | 0.050 |
| rno-miR-26b | 2.414 | ± | 0.100 | 0.000 |
| rno-miR-125b-5p | 2.446 | ± | 0.254 | 0.330 |
| rno-miR-34a | 2.475 | ± | 0.360 | 0.013 |
| rno-miR-152 | 2.890 | ± | 0.417 | 0.010 |
| rno-let-7f | 3.061 | ± | 0.351 | 0.007 |
| rno-miR-16 | 3.082 | ± | 0.923 | 0.039 |
| rno-miR-29c | 3.237 | ± | 0.259 | 0.001 |
| rno-miR-19b | 3.543 | ± | 0.508 | 0.007 |
| rno-miR-200a | 3.603 | ± | 0.106 | 0.000 |
| rno-miR-106b | 4.882 | ± | 0.674 | 0.006 |
| ***rno-miR-192*** | ***5.360*** | ± | 0.089 | 0.016 |
| rno-let-7d | 5.459 | ± | 0.710 | 0.013 |
| rno-miR-101a | 6.964 | ± | 0.706 | 0.004 |
| rno-miR-126 | 7.835 | ± | 0.750 | 0.002 |
| ***rno-miR-144*** | ***7.942*** | ± | 0.171 | 0.046 |
| rno-miR-21 | 9.198 | ± | 0.777 | 0.144 |

| **Rat skeletal muscle** | **fold change ± SEM** | | | | **p-value** |
| --- | --- | --- | --- | --- | --- |
| ***rno-miR-182*** | -4.226 | ± | | 0.548 | 0.023 |
| rno-miR-338* | -2.282 | ± | | 0.017 | 0.000 |
| rno-miR-135b | -1.817 | ± | | 0.036 | 0.034 |
| ***rno-miR-146a*** | -1.654 | ± | | 0.030 | 0.034 |
| rno-miR-487b | -1.603 | ± | | 0.020 | 0.001 |
| rno-miR-181a | -1.543 | ± | | 0.051 | 0.037 |
| rno-miR-412 | -1.480 | ± | | 0.026 | 0.007 |
| rno-miR-199a-3p | -1.458 | ± | 0.005 | | 0.002 |
| rno-miR-375 | -1.447 | ± | 0.028 | | 0.000 |
| rno-miR-183 | -1.410 | ± | 0.029 | | 0.012 |
| rno-miR-208 | -1.251 | ± | 0.029 | | 0.005 |
| rno-miR-363* | -1.249 | ± | 0.038 | | 0.023 |
| rno-miR-100 | 1.493 | ± | 0.067 | | 0.001 |
| rno-miR-191 | 1.499 | ± | 0.028 | | 0.001 |
| rno-miR-34a | 1.542 | ± | 0.205 | | 0.050 |
| ***rno-miR-150*** | 1.567 | ± | 0.015 | | 0.005 |
| rno-miR-10b | 1.577 | ± | 0.065 | | 0.001 |
| rno-miR-146b | 1.581 | ± | 0.101 | | 0.011 |
| ***rno-miR-320*** | 1.604 | ± | 0.145 | | 0.038 |
| rno-miR-451 | 1.609 | ± | 0.001 | | 0.000 |
| rno-miR-24-2* | 1.685 | ± | 0.132 | | 0.020 |
| rno-miR-129 | 1.693 | ± | 0.047 | | 0.005 |
| ***rno-miR-144*** | 1.765 | ± | 0.067 | | 0.001 |
| rno-miR-140* | 1.786 | ± | 0.071 | | 0.003 |
| ***rno-miR-192*** | 1.844 | ± | 0.178 | | 0.043 |
| rno-miR-99a | 1.845 | ± | 0.132 | | 0.008 |
| rno-miR-7a | 1.879 | ± | 0.067 | | 0.003 |
| rno-miR-223 | 2.656 | ± | 0.145 | | 0.000 |
| rno-miR-29b | 2.890 | ± | 0.332 | | 0.008 |
| rno-miR-125b-3p | 2.932 | ± | 0.030 | | 0.000 |
| rno-miR-499 | 3.421 | ± | 0.102 | | 0.000 |
| ***rno-miR-29a*** | 4.959 | ± | 0.179 | | 0.000 |

| **Rat liver** | **fold change ± SEM** | | | | | **p-value** |
| --- | --- | --- | --- | --- | --- | --- |
| ***rno-miR-182*** | -2.613 | | ± | | 0.010 | 0.011 |
| ***rno-miR-146a*** | -2.102 | | ± | | 0.017 | 0.001 |
| ***rno-miR-30d*** | -2.073 | | ± | | 0.006 | 0.001 |
| rno-miR-30a | -1.962 | | ± | | 0.008 | 0.000 |
| rno-miR-30c | -1.825 | | ± | | 0.025 | 0.000 |
| rno-miR-361 | -1.686 | | ± | | 0.028 | 0.003 |
| rno-miR-487b | -1.461 | | ± | | 0.018 | 0.015 |
| rno-miR-30e | -1.408 | | ± | | 0.037 | 0.001 |
| rno-miR-203 | -1.383 | | ± | | 0.044 | 0.003 |
| rno-miR-130a | -1.364 | | ± | | 0.028 | 0.021 |
| rno-miR-30b-5p | -1.269 | | ± | | 0.008 | 0.000 |
| rno-miR-129 | 1.553 | | ± | | 0.044 | 0.005 |
| rno-miR-29b | 1.563 | | ± | | 0.015 | 0.000 |
| rno-miR-142-3p | 1.588 | | ± | | 0.066 | 0.002 |
| ***rno-miR-192*** | 1.591 | | ± | | 0.038 | 0.000 |
| rno-let-7c | 1.616 | | ± | | 0.083 | 0.000 |
| rno-miR-223 | 1.700 | | ± | | 0.110 | 0.011 |
| rno-miR-190 | 1.720 | | ± | | 0.095 | 0.007 |
| rno-let-7i | 1.726 | | ± | | 0.117 | 0.301 |
| rno-miR-125b-5p | 1.726 | | ± | | 0.117 | 0.001 |
| rno-miR-451 | 1.750 | | ± | | 0.084 | 0.001 |
| rno-miR-10a-5p | 1.758 | | ± | | 0.131 | 0.010 |
| rno-miR-423 | 1.759 | | ± | | 0.071 | 0.031 |
| rno-miR-146b | 1.776 | | ± | | 0.195 | 0.013 |
| rno-miR-26a | 1.872 | | ± | | 0.071 | 0.000 |
| rno-miR-191 | 1.889 | | ± | | 0.044 | 0.003 |
| rno-miR-125b* | 1.906 | | ± | | 0.077 | 0.002 |
| rno-miR-21 | 1.916 | | ± | | 0.003 | 0.000 |
| rno-miR-378 | 1.948 | | ± | | 0.076 | 0.001 |
| rno-miR-145 | 2.050 | | ± | | 0.098 | 0.002 |
| rno-miR-148b-3p | 2.053 | | ± | | 0.057 | 0.044 |
| ***rno-miR-150*** | 2.068 | | ± | | 0.388 | 0.028 |
| rno-let-7b | 2.126 | | ± | | 0.238 | 0.011 |
| rno-miR-375 | 2.184 | | ± | | 0.273 | 0.001 |
| rno-miR-100 | 2.221 | ± | | 0.203 | | 0.027 |
| rno-miR-29c | 2.285 | ± | | 0.051 | | 0.000 |
| ***rno-miR-29a*** | 2.359 | ± | | 0.044 | | 0.000 |
| rno-miR-23a | 2.433 | ± | | 0.056 | | 0.000 |
| rno-miR-103 | 2.449 | ± | | 0.280 | | 0.008 |
| rno-miR-125a-5p | 2.508 | ± | | 0.080 | | 0.000 |
| rno-miR-99b | 2.535 | ± | | 0.295 | | 0.012 |
| rno-miR-208 | 2.583 | ± | | 0.361 | | 0.022 |
| rno-miR-214 | 2.636 | ± | | 0.029 | | 0.014 |
| ***rno-miR-320*** | 2.677 | ± | | 0.143 | | 0.001 |
| ***rno-miR-144*** | 4.263 | ± | | 0.174 | | 0.000 |
