# Supplementary material for: MicroRNA 144 Impairs Insulin Signaling by Inhibiting the Expression of Insulin Receptor Substrate 1 in Type 2 Diabetes Mellitus
Source: PLoS One. 2011 Aug 1;6(8):e22839. doi: 10.1371/journal.pone.0022839 (PMC3148231; doi:10.1371/journal.pone.0022839)
Supplement: Table S4 — miRNAs expressed in IFG and T2D from batch A. miRNAs whose significant changes replicated in at least 50% of the subjects in any of the two categories (IFG; n = 6, or T2D; n = 8) as compared to CTL; (n = 7) are shown. Values of miRNAs that were differentially expressed but showed no significant fold change (p>0.05) in at least 50% of the subjects in any of the two categories were not included. Values are fold changes calculated as a ratio of IFG or T2D versus control (CTL) with p-values in 2 decimal places. Fold change values below 1 are expressed as the negative values. Statistically significant differences are tested using Student's t-test at p<0.05 significance. miRNAs that are significantly expressed in both IFG and T2D are shown in bold. CTL, healthy controls; IFG, impaired fasting glucose; T2D, type 2 diabetes. (DOC) [file pone.0022839.s004.doc]

**S4: miRNAs expressed in IFG and T2D from batch A.** miRNAs whose significant changes replicated in at least 50% of the subjects in any of the two categories (IFG; n=6, or T2D; n=8) as compared to CTL; (n=7) are shown. Values of miRNAs that were differentially expressed but showed no significant fold change (p>0.05) in at least 50% of the subjects in any of the two categories were not included. Values are fold changes calculated as a ratio of IFG or T2D versus control (CTL) with p-values in 2 decimal places. Fold change values below 1 are expressed as the negative values. Statistically significant differences are tested using Student’s t-test at p<0.05 significance. miRNAs that are significantly expressed in both IFG and T2D are shown in **bold**. CTL, healthy controls; IFG, impaired fasting glucose; T2D, type 2 diabetes.

| **hsa-miRNA** | **IFG** | **T2D** | **hsa-miRNA** | **IFG** | **T2D** | **hsa-miRNA** | **IFG** | **T2D** |
| --- | --- | --- | --- | --- | --- | --- | --- | --- |
|  | **(p-value)** | **(p-value)** |  | **(p-value)** | **(p-value)** |  | **(p-value)** | **(p-value)** |
| hsa-let-7a | -1.56 (0.00) |  | **miR-1297** | -1.73 (0.00) | -2.25 (0.00) | **miR-190** | -1.72 (0.03) | +2.37 (0.02) |
| hsa-let-7b | -1.99 (0.00) |  | miR-1299 | -1.83 (0.00) |  | **miR-192** | +1.27 (0.05) | +2.48 (0.00) |
| hsa-let-7b* |  | -1.40 (0.03) | miR-1301 |  | +1.87 (0.02) | miR-193a-3p |  | +1.82 (0.00) |
| hsa-let-7d | -2.85 (0.00) | -1.59 (0.01) | **miR-130a** | -2.17 (0.03) | +1.94 (0.01) | miR-194 |  | +2.10 (0.00) |
| hsa-let-7d* | -1.65 (0.00) |  | miR-130b |  | +2.00 (0.00) | miR-195 | -1.97 (0.01) |  |
| hsa-let-7e |  | -1.56 (0.04) | miR-142-3p |  | +1.69 (0.00) | **miR-19a** | +1.51 (0.00) | -1.91 (0.03) |
| hsa-let-7g |  | -2.08 (0.00) | miR-142-5p | +1.87 (0.00) |  | miR-20a |  | -1.67 (0.00) |
| hsa-let-7i |  | -1.57 (0.00) | **miR-144** | +1.95 (0.03) | +2.67 (0.02) | miR-20b |  | -2.33 (0.00) |
| hsa-miR-103 | -1.91 (0.00) |  | **miR-146a** | -1.58 (0.00) | -2.54 (0.01) | miR-221 |  | +2.05 (0.03) |
| **miR-106a** | -1.78 (0.00) | -2.54 (0.00) | miR-146b-5p |  | -2.48 (0.00) | miR-222 |  | +1.86 (0.03) |
| **miR-106b** | +1.84 (0.03) | +1.68 (0.03) | miR-148b | +2.11 (0.04) |  | **miR-23a** | +2.18 (0.00) | +1.86 (0.00) |
| **miR-1184** | -3.19 (0.00) | -2.42 (0.01) | **miR-150** | -2.92 (0.02) | +2.17 (0.01) | miR-23b |  | +1.79 (0.00) |
| miR-125a-5p |  | +2.11 (0.00) | miR-151-3p |  | +2.00 (0.03) | **miR-26a** | -1.65 (0.04) | +2.22 (0.03) |
| miR-125b |  | +1.85 (0.01) | miR-151-5p |  | +1.99 (0.00) | miR-26b |  | +1.60 (0.01) |
| miR-125b-1* | -3.19 (0.00) |  | miR-15a |  | +2.15 (0.02) | **miR-27a** | +2.00 (0.00) | +2.28 (0.02) |
| **miR-126** | +2.29 (0.04) | +1.51 (0.02) | **miR-16-2*** | +2.29 (0.00) |  | miR-299-3p |  | +1.54 (0.03) |
| miR-1261 | -3.76 (0.00) |  | miR-17 |  | -1.59 (0.00) | **miR-29a** | +1.88 (0.04) | +2.46 (0.01) |
| miR-1264 | -2.25 (0.01) |  | **miR-17*** | +1.77 (0.00) | +1.95 (0.01) | miR-29b |  | +2.38 (0.00) |
| miR-1265 | -3.56 (0.00) |  | **miR-182** | +1.34 (0.01) | -2.58 (0.04) | miR-29c |  | +1.71 (0.02) |
| miR-1275 | -1.62 (0.02) |  | **miR-183** | +1.79 (0.01) | +1.74 (0.01) | miR-30b | +2.10 (0.04) |  |
| miR-1280 | -2.88 (0.01) |  | **miR-185** | -1.62 (0.02) | +1.82 (0.00) | **miR-30c** | +2.04 (0.03) | -1.73 (0.01) |
| miR-1285 |  | +1.67 (0.01) | miR-185* |  | -1.65 (0.01) | **miR-30d** | +1.61 (0.00) | -2.89 (0.00) |
| **miR-129-5p** | -2.65 (0.00) | -1.81 (0.01) | **miR-186** | -1.77 (0.00) | -2.33 (0.01) | **miR-30e** | +1.90 (0.01) | -2.91 (0.02) |

| **hsa-miRNA** | **IFG** | **T2D** | **hsa-miRNA** | **IFG** | **T2D** | **hsa-miRNA** | **IFG** | **T2D** |
| --- | --- | --- | --- | --- | --- | --- | --- | --- |
|  | **(p-value)** | **(p-value)** |  | **(p-value)** | **(p-value)** |  | **(p-value)** | **(p-value)** |
| miR-30e* | +1.98 (0.00) |  | miR-527 | -1.63 (0.00) |  | **miR-7** | +1.89 (0.00) | -1.85 (0.04) |
| miR-32* | -2.72 (0.01) |  | miR-519d | -1.57 (0.00) |  | miR-765 | -1.81 (0.01) |  |
| **miR-320a** | -1.47 (0.01) | +1.93 (0.04) | miR-519e |  | -2.10 (0.00) | miR-766 | -1.58 (0.00) |  |
| **miR-320b** | -1.71 (0.01) | +1.98 (0.02) | miR-532-3p |  | -1.54 (0.04) | miR-877 | -2.29 (0.00) |  |
| **miR-320c** | -1.82 (0.00) | +2.05 (0.03) | miR-549 | -1.54 (0.02) |  | miR-886-5p |  | +2.33 (0.01) |
| **miR-320d** | -1.92 (0.02) | +2.23 (0.01) | **miR-550** | -1.91 (0.00) | +1.85 (0.04) | miR-887 | +1.66 (0.00) |  |
| miR-335 |  | +2.29 (0.00) | **miR-550*** | -1.61 (0.00) | +2.06 (0.00) | miR-923 |  | -2.64 (0.03) |
| miR-340 |  | -1.59 (0.01) | miR-551b | -1.53 (0.00) |  | miR-939 | -1.93 (0.00) |  |
| miR-342-3p |  | -1.76 (0.00) | miR-576-5p | -1.89 (0.00) |  | **miR-99b*** | -3.15 (0.00) | +1.61 (0.00) |
| miR-34b |  | -1.71 (0.01) | miR-583 | -2.27 (0.00) |  |  |  |  |
| miR-345 | -2.14 (0.00) |  | miR-589 |  | +1.68 (0.04) |  |  |  |
| **miR-361-3p** | -1.81 (0.00) | +1.57 (0.01) | miR-602 | -1.73 (0.00) |  |  |  |  |
| miR-362-5p |  | -2.14 (0.01) | **miR-620** | -2.66 (0.00) | +2.07 (0.01) |  |  |  |
| miR-363 | -1.80 (0.01) |  | miR-628-3p | -2.32 (0.00) |  |  |  |  |
| miR-374b |  | -1.63 (0.03) | miR-629 |  | +1.94 (0.01) |  |  |  |
| miR-375 |  | +2.00 (0.00) | miR-634 | -2.83 (0.00) |  |  |  |  |
| miR-484 | +1.90 (0.02) |  | **miR-636** | -2.45 (0.00) | -2.37 (0.02) |  |  |  |
| miR-491-3p | -1.99 (0.00) |  | **miR-637** | -1.93 (0.00) | -2.44 (0.00) |  |  |  |
| miR-494 | -2.61 (0.03) |  | miR-642 | -1.74 (0.01) |  |  |  |  |
| miR-500 | +1.59 (0.04) |  | miR-647 | -2.04 (0.04) |  |  |  |  |
| miR-500* | -1.52 (0.00) |  | miR-652 |  | -2.06 (0.04) |  |  |  |
| miR-502-3p |  | +2.30 (0.02) | miR-660 |  | -2.00 (0.03) |  |  |  |
| miR-518a-5p | -1.63 (0.01) |  | miR-665 |  | +1.65 (0.04) |  |  |  |
