# Supplementary material for: MicroRNA 144 Impairs Insulin Signaling by Inhibiting the Expression of Insulin Receptor Substrate 1 in Type 2 Diabetes Mellitus
Source: PLoS One. 2011 Aug 1;6(8):e22839. doi: 10.1371/journal.pone.0022839 (PMC3148231; doi:10.1371/journal.pone.0022839)

S5: Pearson correlation scatter plot of miRNA expressions (BatchA). (A). miRNA profiles of IFG patients do not correlate strongly to those of CTL at *R*=0.78; in white circles while (**B).** the miRNA profiles of T2D patients showed a much weaker correlation to CTL at a further reduced *R*=0.68; in white squares. The scatter plot of~100 detected miRNAs is drawn to provide a snap shot of the distribution and the correlation between the two variables. Points closely clustered to a straightline indicate a stronger correlation between the variables.The plot shows that IFG and CTL has stronger association than T2D and CTL. CTL,healthy controls; IFG,impaired fasting glucose; T2D,type 2 diabetes.

a

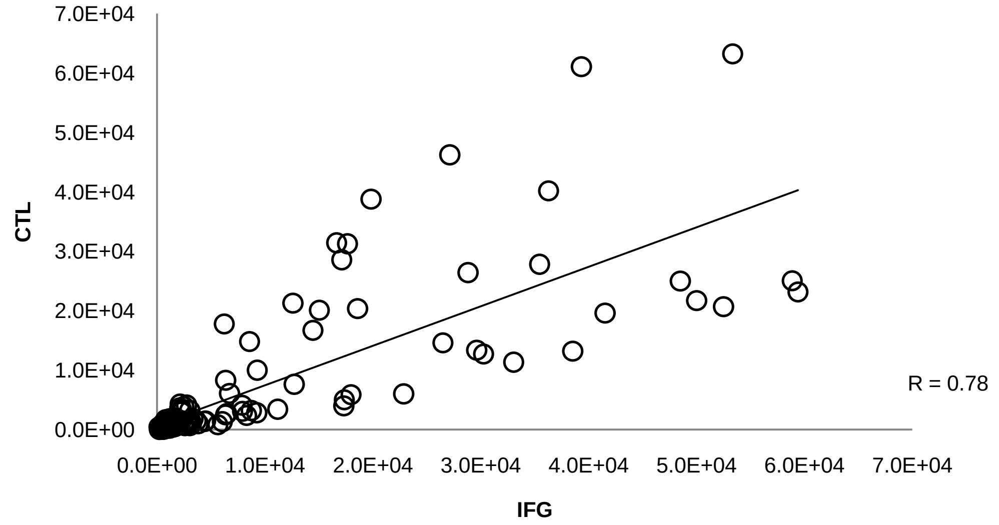

b

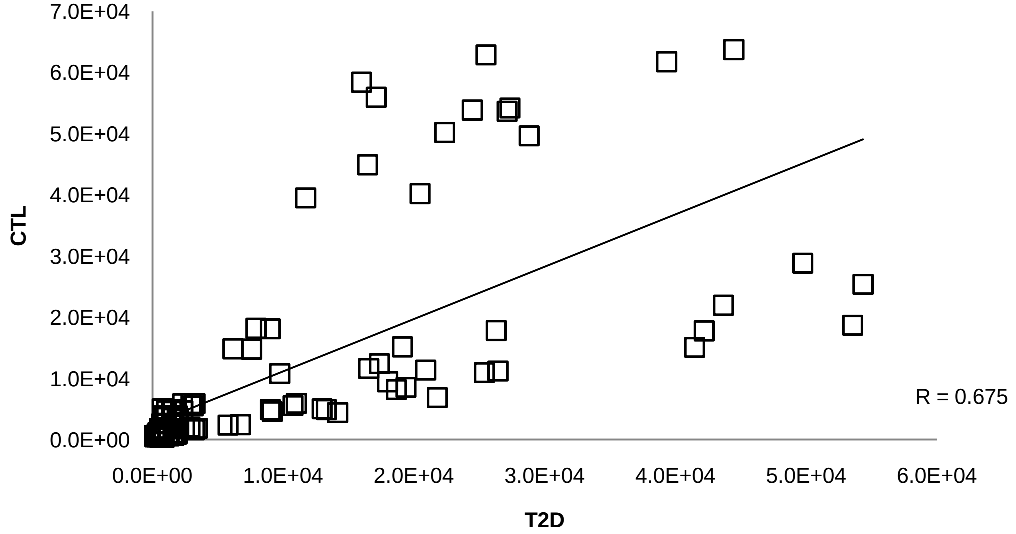

Supplement: Table S5 — Pearson correlation scatter plot of miRNA expressions (BatchA). (A). miRNA profiles of IFG patients do not correlate strongly to those of CTL at R = 0.78; in white circles while (B). the miRNA profiles of T2D patients showed a much weaker correlation to CTL at a further reduced R = 0.68; in white squares. The scatter plot of ∼100 detected miRNAs is drawn to provide a snap shot of the distribution and the correlation between the two variables. Points closely clustered to a straightline indicate a stronger correlation between the variables.The plot shows that IFG and CTL has stronger association than T2D and CTL. CTL,healthy controls; IFG,impaired fasting glucose; T2D,type 2 diabetes. (PDF) [file pone.0022839.s005.pdf]
