# Supplementary material for: MicroRNA 144 Impairs Insulin Signaling by Inhibiting the Expression of Insulin Receptor Substrate 1 in Type 2 Diabetes Mellitus
Source: PLoS One. 2011 Aug 1;6(8):e22839. doi: 10.1371/journal.pone.0022839 (PMC3148231; doi:10.1371/journal.pone.0022839)

S7: PCA plot of mRNA profiles of IFG and T2D patients (Batch A). Principal component analysis (PCA) plot based on mRNA expression showed a less distinguished classification of IFG and T2D patients. Batch A patients consists of six IFG patients (labeled as 1IFG to 6IFG) and eight T2D patients (labeled as 1T2D to 8T2D). IFG,impaired fasting glucose; T2D,type 2 diabetes.

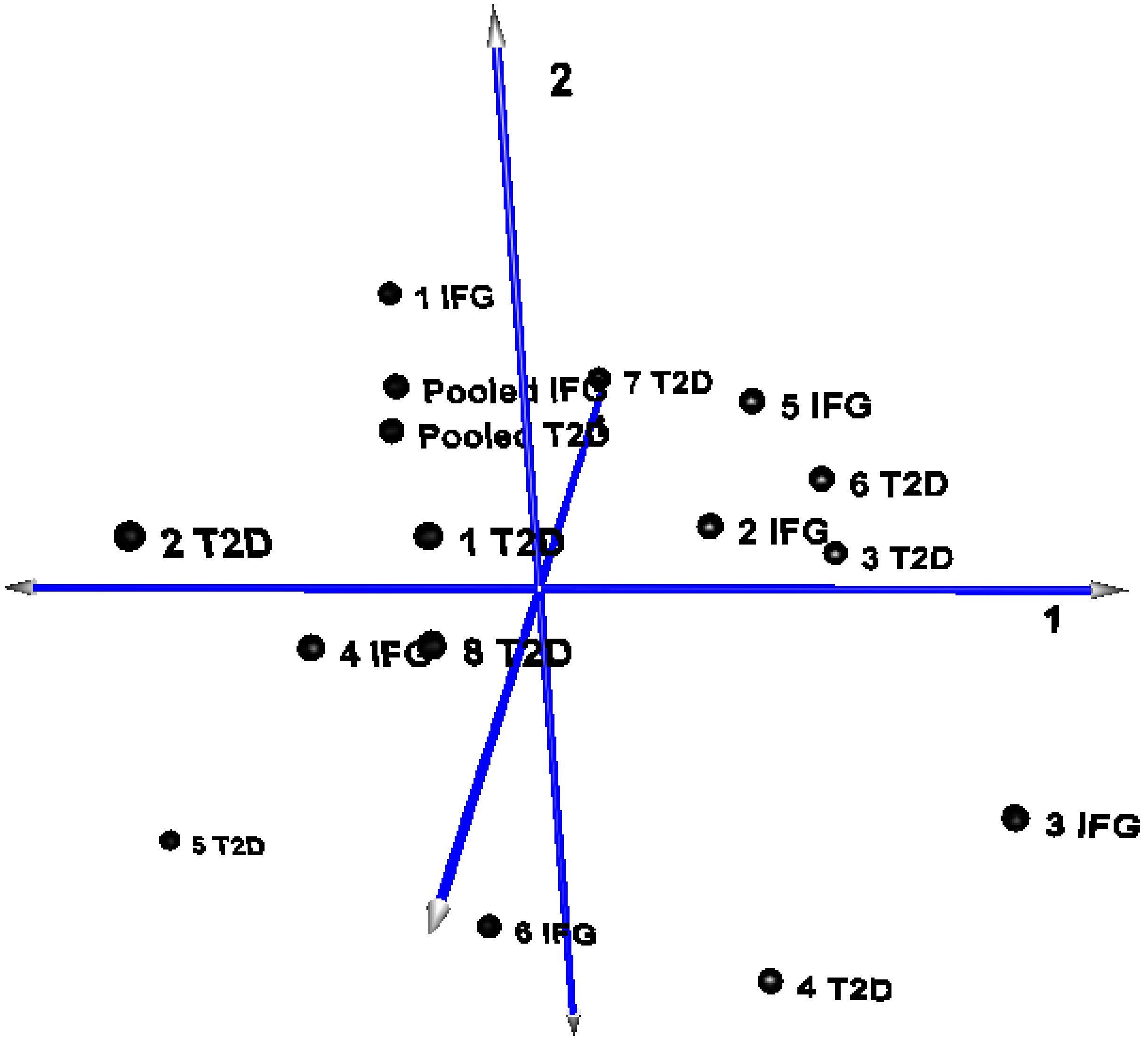

Supplement: Table S7 — PCA plot of mRNA profiles of IFG and T2D patients (Batch A). Principal component analysis (PCA) plot based on mRNA expression showed a less distinguished classification of IFG and T2D patients. Batch A patients consists of six IFG patients (labeled as 1IFG to 6IFG) and eight T2D patients (labeled as 1T2D to 8T2D). IFG,impaired fasting glucose; T2D,type 2 diabetes. (PDF) [file pone.0022839.s007.pdf]
