# Supplementary material for: MicroRNA 144 Impairs Insulin Signaling by Inhibiting the Expression of Insulin Receptor Substrate 1 in Type 2 Diabetes Mellitus
Source: PLoS One. 2011 Aug 1;6(8):e22839. doi: 10.1371/journal.pone.0022839 (PMC3148231; doi:10.1371/journal.pone.0022839)
Supplement: Table S8 — Validation of microRNAs and mRNAs microarray. The microarray data for the expression of the 8 “signature” miRNAs and their respective target mRNAs were validated using quantitative real-time PCR. Each miRNA/mRNA were assayed in triplicates for 3 separate experiments and the relative expression values are stated ± SEM. Fold change for the real-time PCR data was computed from the 2−ΔΔCt values. Statistically significant differences are tested using Student's t-test at p<0.05 significance. CTL, healthy controls; IFG, impaired fasting glucose; T2D, type 2 diabetes. (DOC) [file pone.0022839.s008.doc]

| **microRNA expression** | | | | | **mRNA expression** | | | | |
| --- | --- | --- | --- | --- | --- | --- | --- | --- | --- |
| **miRNA** | **IFG** | | **T2D** | | **mRNA** | **IFG** | | **T2D** | |
|  | Microarray [p-value] | Quantitative stem-loop RT-PCR [p-value] | Microarray [p-value] | Quantitative stem-loop RT-PCR [p-value] |  | Microarray [p-value] | Quantitative real-time PCR [p-value] | Microarray [p-value] | Quantitative real-time PCR [p-value] |
|  |  |  |  |  |  |  |  |  |  |
|  |  |  |  |  |  |  |  |  |  |
|  |  |  |  |  |  |  |  |  |  |
| miR-144 | +1.945±0.16 | +1.385±0.14 | +2.565±0.10 | +3.070±0.13 | *IRS1* | -1.296±0.01 | -1.577±0.06 | -1.305±0.04 | -2.568±0.03 |
|  | [0.03] | [0.03] | [0.02] | [0.00] |  | [0.04] | [0.00] | [0.01] | [0.00] |
| miR-146a | -1.584±0.09 | -1.399±0.16 | -2.544±0.06 | -3.375±0.13 | *PTPN1* | +1.188±0.12 | -1.055±0.09 | +1.920±0.10 | +1.781±0.10 |
|  | [0.00] | [0.02] | [0.01] | [0.00] |  | [0.11] | [0.01] | [0.00] | [0.00] |
| miR-150 |  |  |  |  | *GLUT4* | +1.633±0.15 | +1.787±0.17 | -1.748±0.08 | -2.157±0.06 |
|  | -2.919±0.05 | -1.862±0.09 | +2.169±0.06 | +2.845±0.09 |  | [0.00] | [0.01] | [0.04] | [0.00] |
|  | [0.02] | [0.00] | [0.01] | [0.00] | *CBL* | +1.428±0.05 | +1.592±0.06 | -1.575±0.05 | -1.367±0.15 |
|  |  |  |  |  |  | [0.01] | [0.04] | [0.01] | [0.00] |
| miR-182 | +1.342±0.05 | +1.355±0.09 | -2.577±0.05 | -3.535±0.05 | *FOXO1* | -1.614±0.11 | -1.327±0.13 | +1.723±0.10 | +2.188±0.07 |
|  | [0.01] | [0.02] | [0.04] | [0.00] |  | [0.03] | [0.07] | [0.00] | [0.00] |
| miR-192 | +1.271±0.18 | +1.139±0.14 | +2.481±0.10 | +2.338±0.08 | *INSR* | +1.042±0.13 | +1.025±0.11 | -2.517±0.06 | -2.568±0.07 |
|  | [0.05] | [0.21] | [0.00] | [0.00] |  | [0.09] | [0.18] | [0.00] | [0.00] |
| miR-30d | +1.610±0.14 | +2.672±0.15 | -2.894±0.04 | -1.377±0.10 | *INS* | +1.385±0.06 | +1.722±0.13 | -1.678±0.04 | -1.555±0.09 |
|  | [0.00] | [0.00] | [0.00] | [0.04] |  | [0.01] | [0.04] | [0.02] | [0.01] |
| miR-29a | +1.878±0.09 | +1.323±0.15 | +2.464±0.15 | +2.087±0.14 | *AKT2* |  |  |  |  |
|  | [0.04] | [0.03] | [0.01] | [0.01] |  | +1.038±0.12 | +1.071±0.08 | -1.286±0.10 | -1.692±0.12 |
| miR-320a | -1.470±0.05 | -1.484±0.09 | +1.926±0.11 | +3.607±0.16 |  | [0.15] | [0.24] | [0.01] | [0.01] |
|  | [0.01] | [0.00] | [0.04] | [0.00] |  |  |  |  |  |

**S8: Validation of microRNAs and mRNAs microarray.** The microarray data for the expression of the 8 “signature” miRNAs and their respective target mRNAs were validated using quantitative real-time PCR. Each miRNA/mRNA were assayed in triplicates for 3 separate experiments and the relative expression values are stated ± SEM. Fold change for the real-time PCR data was computed from the 2^-ΔΔCt^ values. Statistically significant differences are tested using Student’s t-test at p<0.05 significance. CTL, healthy controls; IFG, impaired fasting glucose; T2D, type 2 diabetes
