# Supplementary material for: MicroRNA 144 Impairs Insulin Signaling by Inhibiting the Expression of Insulin Receptor Substrate 1 in Type 2 Diabetes Mellitus
Source: PLoS One. 2011 Aug 1;6(8):e22839. doi: 10.1371/journal.pone.0022839 (PMC3148231; doi:10.1371/journal.pone.0022839)
Supplement: Table S9 — Quantitative Real-time PCR results for Batch B individuals ( Fig. 6A&B ). The 8 “signature” miRNAs (A) and their respective target mRNAs (B) were validated using quantitative real-time PCR in the Batch B blood samples. Each miRNA/mRNA were assayed in triplicates for 3 separate experiments and the relative expression values are stated ± SEM. Fold change for the real-time PCR data was computed from the 2−ΔΔCt values. Statistically significant differences are tested using Student's t-test at p<0.05 significance. CTL, healthy controls; IFG, impaired fasting glucose; T2D, type 2 diabetes. (DOC) [file pone.0022839.s009.doc]

**S9: Quantitative Real-time PCR results for Batch B individuals (Fig. 6A&B).** The 8 “signature” miRNAs **(A)** and their respective target mRNAs **(B)** were validated using quantitative real-time PCR in the Batch B blood samples. Each miRNA/mRNA were assayed in triplicates for 3 separate experiments and the relative expression values are stated ± SEM. Fold change for the real-time PCR data was computed from the 2^-ΔΔCt^ values. Statistically significant differences are tested using Student’s t-test at p<0.05 significance. CTL, healthy controls; IFG, impaired fasting glucose; T2D, type 2 diabetes.

**S9**A

|  | **Type 2 Diabetes (T2D)-miRNA Fold change (T2D vs Control)±SEM** | | | | | | | | | |
| --- | --- | --- | --- | --- | --- | --- | --- | --- | --- | --- |
| **Patient**  **/miRNA** | **T2D 9** | **p-value** | **T2D 10** | **p-value** | **T2D 11** | **p-value** | **T2D 12** | **p-value** | **T2D 13** | **p-value** |
| miR-144 | 1.381±0.102 | 0.025 | 1.252±0.034 | 0.001 | 2.000±0.059 | 0.012 | 3.131±0.014 | 0.000 | 1.386±0.019 | 0.000 |
| miR-146a | 0.650±0.110 | 0.004 | 0.218±0.072 | 0.000 | 0.945±0.022 | 0.006 | 0.649±0.139 | 0.004 | 0.532±0.012 | 0.008 |
| miR-150 | 2.433±0.046 | 0.000 | 1.134±0.293 | 0.014 | 1.783±0.012 | 0.122 | 1.516±0.037 | 0.009 | 1.405±0.089 | 0.000 |
| miR-182 | 0.300±0.036 | 0.000 | 0.044±0.035 | 0.000 | 0.434±0.010 | 0.000 | 0.307±0.023 | 0.000 | 0.064±0.050 | 0.000 |
| miR-192 | 3.046±0.042 | 0.000 | 1.768±0.043 | 0.000 | 4.724±0.028 | 0.000 | 3.069±0.010 | 0.000 | 2.362±0.237 | 0.000 |
| miR-29a | 1.419±0.176 | 0.036 | 1.074±0.028 | 0.028 | 2.082±0.205 | 0.019 | 1.265±0.038 | 0.154 | 1.022±0.067 | 0.077 |
| miR-30d | 0.889±0.028 | 0.028 | 0.120±0.026 | 0.000 | 0.769±0.028 | 0.009 | 0.450±0.010 | 0.000 | 0.171±0.111 | 0.000 |
| miR-320 | 3.487±0.033 | 0.000 | 1.490±0.009 | 0.000 | 2.478±0.024 | 0.000 | 1.196±0.016 | 0.034 | 1.064±0.024 | 0.121 |
| **Patient**  **/miRNA** | **T2D 14** | **p-value** | **T2D 15** | **p-value** | **T2D 16** | **p-value** | **T2D 17** | **p-value** | **T2D 18** | **p-value** |
| miR-144 | 1.668±0.022 | 0.000 | 2.800±0.030 | 0.000 | 1.880±0.010 | 0.010 | 1.499±0.009 | 0.028 | 2.383±0.036 | 0.000 |
| miR-146a | 0.386±0.078 | 0.000 | 0.714±0.039 | 0.016 | 0.604±0.345 | 0.000 | 0.464±0.080 | 0.000 | 0.250±0.120 | 0.000 |
| miR-150 | 1.092±0.017 | 0.137 | 1.751±0.023 | 0.001 | 1.648±0.180 | 0.035 | 1.708±0.017 | 0.005 | 1.228±0.158 | 0.015 |
| miR-182 | 0.275±0.019 | 0.000 | 0.579±0.025 | 0.027 | 0.409±0.021 | 0.000 | 0.262±0.032 | 0.000 | 0.051±0.046 | 0.000 |
| miR-192 | 2.713±0.016 | 0.000 | 3.130±0.020 | 0.000 | 3.567±0.089 | 0.000 | 3.515±0.045 | 0.000 | 1.599±0.036 | 0.000 |
| miR-29a | 1.299±0.004 | 0.011 | 1.919±0.045 | 0.000 | 1.165±0.166 | 0.091 | 1.479±0.242 | 0.019 | 1.143±0.027 | 0.205 |
| miR-30d | 0.224±0.027 | 0.000 | 0.651±0.019 | 0.006 | 0.649±0.006 | 0.000 | 1.141±0.011 | 0.096 | 0.157±0.017 | 0.000 |
| miR-320 | 1.652±0.023 | 0.000 | 3.377±0.006 | 0.000 | 2.372±0.018 | 0.028 | 2.520±0.030 | 0.000 | 0.975±0.017 | 0.103 |
| **Patient**  **/miRNA** | **T2D 19** | **p-value** | **T2D 20** | **p-value** | **T2D 21** | **p-value** |  |  |  |  |
| miR-144 | 2.355±0.044 | 0.000 | 1.713±0.034 | 0.000 | 2.279±0.021 | 0.000 |  |  |  |  |
| miR-146a | 0.571±0.168 | 0.001 | 0.993±0.137 | 0.588 | 0.902±0.048 | 0.172 |  |  |  |  |
| miR-150 | 1.035±0.020 | 0.079 | 1.111±0.054 | 0.141 | 1.258±0.101 | 0.001 |  |  |  |  |
| miR-182 | 0.113±0.057 | 0.000 | 0.180±0.038 | 0.000 | 0.242±0.036 | 0.000 |  |  |  |  |
| miR-192 | 1.470±0.032 | 0.016 | 1.631±0.090 | 0.001 | 1.979±0.011 | 0.000 |  |  |  |  |
| miR-29a | 1.126±0.044 | 0.054 | 1.666±0.127 | 0.000 | 1.595±0.121 | 0.000 |  |  |  |  |
| miR-30d | 0.164±0.014 | 0.000 | 0.503±0.023 | 0.001 | 0.748±0.031 | 0.042 |  |  |  |  |
| miR-320 | 0.961±0.006 | 0.168 | 2.293±0.004 | 0.000 | 1.309±0.009 | 0.000 |  |  |  |  |
|  | **Impaired fasting glucose (IFG) – miRNA Fold change (IFG vs Control)±SEM** | | | | | | | | | |
|  |
| **Patient**  **/miRNA** | **IFG 7** | **p-value** | **IFG 8** | **p-value** | **IFG 9** | **p-value** | **IFG 10** | **p-value** | **IFG 11** | **p-value** |
| miR-144 | 1.383±0.033 | 0.151 | 1.148±0.004 | 0.000 | 1.352±0.012 | 0.009 | 0.755±0.075 | 0.098 | 0.996±0.081 | 0.137 |
| miR-146a | 1.326±0.108 | 0.051 | 0.944±0.038 | 0.053 | 0.857±0.022 | 0.005 | 0.587±0.036 | 0.009 | 0.820±0.057 | 0.006 |
| miR-150 | 0.596±0.029 | 0.000 | 0.598±0.023 | 0.020 | 0.711±0.023 | 0.000 | 0.883±0.063 | 0.021 | 1.452±0.053 | 0.031 |
| miR-182 | 1.197±0.027 | 0.183 | 0.726±0.030 | 0.009 | 1.584±0.004 | 0.053 | 1.135±0.043 | 0.106 | 0.948±0.036 | 0.235 |
| miR-192 | 0.886±0.030 | 0.046 | 1.019±0.029 | 0.120 | 1.987±0.011 | 0.016 | 1.270±0.028 | 0.002 | 1.029±0.028 | 0.150 |
| miR-29a | 1.488±0.176 | 0.013 | 1.249±0.038 | 0.000 | 1.804±0.027 | 0.034 | 1.881±0.055 | 0.000 | 1.422±0.057 | 0.040 |
| miR-30d | 1.536±0.025 | 0.012 | 0.964±0.007 | 0.061 | 1.301±0.007 | 0.008 | 1.466±0.024 | 0.011 | 1.006±0.028 | 0.125 |
| miR-320 | 0.753±0.008 | 0.000 | 0.464±0.029 | 0.001 | 0.896±0.017 | 0.042 | 0.983±0.039 | 0.151 | 0.755±0.420 | 0.003 |
| **Patient**  **/miRNA** | **IFG 12** | **p-value** | **IFG 13** | **p-value** | **IFG 14** | **p-value** |  |  |  |  |
| miR-144 | 1.141±0.016 | 0.176 | 0.929±0.110 | 0.170 | 1.340±0.023 | 0.000 |  |  |  |  |
| miR-146a | 0.870±0.148 | 0.001 | 0.547±0.020 | 0.012 | 0.627±0.068 | 0.047 |  |  |  |  |
| miR-150 | 1.093±0.038 | 0.172 | 0.851±0.012 | 0.103 | 0.839±0.026 | 0.015 |  |  |  |  |
| miR-182 | 1.482±0.045 | 0.002 | 1.068±0.006 | 0.091 | 1.496±0.039 | 0.006 |  |  |  |  |
| miR-192 | 1.319±0.025 | 0.051 | 1.319±0.058 | 0.000 | 1.644±0.009 | 0.000 |  |  |  |  |
| miR-29a | 1.310±0.146 | 0.038 | 0.740±0.251 | 0.061 | 0.886±0.220 | 0.096 |  |  |  |  |
| miR-30d | 1.217±0.012 | 0.153 | 2.467±0.009 | 0.000 | 1.533±0.022 | 0.000 |  |  |  |  |
| miR-320 | 0.793±0.018 | 0.050 | 0.854±0.033 | 0.044 | 1.163±0.018 | 0.000 |  |  |  |  |

**S9B**

|  | **Type 2 Diabetes (T2D)-mRNA Fold change (T2D vs Control)±SEM** | | | | | | | | | |
| --- | --- | --- | --- | --- | --- | --- | --- | --- | --- | --- |
| **Patient**  **/mRNA** | **T2D 9** | **p-value** | **T2D 10** | **p-value** | **T2D 11** | **p-value** | **T2D 12** | **p-value** | **T2D 13** | **p-value** |
| *IRS1* | 0.359±0.020 | 0.000 | 0.126±0.026 | 0.000 | 0.520±0.013 | 0.048 | 0.370±0.017 | 0.001 | 0.480±0.033 | 0.039 |
| *PTPN1* | 1.684±0.067 | 0.000 | 1.424±0.026 | 0.000 | 2.728±0.040 | 0.000 | 1.107±0.050 | 0.000 | 2.702±0.031 | 0.000 |
| *GLUT4* | 0.733±0.043 | 0.041 | 0.278±0.057 | 0.033 | 0.876±0.038 | 0.028 | 0.487±0.035 | 0.047 | 0.800±0.019 | 0.011 |
| *CBL* | 0.397±0.021 | 0.072 | 0.404±0.029 | 0.037 | 0.747±0.017 | 0.110 | 0.362±0.027 | 0.071 | 0.654±0.013 | 0.048 |
| *FOXO* | 1.123±0.123 | 0.069 | 2.15±0.129 | 0.000 | 1.893±0.057 | 0.015 | 1.024±0.102 | 0.083 | 1.624±0.083 | 0.011 |
| *INSR* | 0.300±0.004 | 0.001 | 0.104±0.026 | 0.000 | 0.425±0.013 | 0.194 | 0.262±0.106 | 0.001 | 0.400±0.024 | 0.052 |
| *INS* | 0.647±0.065 | 0.605 | 0.445±0.016 | 0.048 | 0.507±0.033 | 0.038 | 0.229±0.031 | 0.000 | 0.582±0.047 | 0.019 |
| *AKT2* | 0.562±0.081 | 0.702 | 0.279±0.051 | 0.000 | 0.796±0.020 | 0.001 | 0.308±0.076 | 0.000 | 0.992±0.006 | 0.000 |
| **Patient**  **/mRNA** | **T2D 14** | **p-value** | **T2D 15** | **p-value** | **T2D 16** | **p-value** | **T2D 17** | **p-value** | **T2D 18** | **p-value** |
| *IRS1* | 0.696±0.034 | 0.042 | 0.684±0.022 | 0.066 | 0.577±0.033 | 0.000 | 0.375±0.028 | 0.005 | 0.525±0.010 | 0.000 |
| *PTPN1* | 2.191±0.054 | 0.000 | 1.398±0.018 | 0.000 | 1.667±0.021 | 0.000 | 1.954±0.008 | 0.000 | 2.069±0.012 | 0.000 |
| *GLUT4* | 0.860±0.011 | 0.019 | 0.555±0.022 | 0.048 | 0.359±0.041 | 0.000 | 0.645±0.057 | 0.026 | 0.526±0.007 | 0.008 |
| *CBL* | 1.291±0.012 | 0.053 | 0.442±0.009 | 0.007 | 0.477±0.023 | 0.005 | 0.499±0.006 | 0.009 | 0.524±0.016 | 0.006 |
| *FOXO* | 1.534±0.085 | 0.006 | 2.106±0.022 | 0.003 | 1.646±0.138 | 0.026 | 2.745±0.063 | 0.000 | 2.015±0.029 | 0.009 |
| *INSR* | 0.443±0.018 | 0.001 | 0.304±0.056 | 0.000 | 0.373±0.023 | 0.000 | 0.349±0.039 | 0.039 | 0.399±0.039 | 0.000 |
| *INS* | 0.823±0.027 | 0.000 | 0.279±0.021 | 0.000 | 0.356±0.002 | 0.001 | 1.039±0.005 | 0.080 | 0.708±0.013 | 0.010 |
| *AKT2* | 0.846±0.015 | 0.049 | 0.368±0.123 | 0.000 | 0.398±0.031 | 0.000 | 0.583±0.040 | 0.018 | 0.978±0.009 | 0.094 |
| **Patient /mRNA** | **T2D 19** | **p-value** | **T2D 20** | **p-value** | **T2D 21** | **p-value** |  |  |  |  |
| *IRS1* | 0.577±0.032 | 0.061 | 0.469±0.020 | 0.000 | 0.163±0.021 | 0.000 |  |  |  |  |
| *PTPN1* | 2.357±0.046 | 0.000 | 1.776±0.126 | 0.000 | 1.582±0.040 | 0.000 |  |  |  |  |
| *GLUT4* | 0.980±0.079 | 0.012 | 0.630±0.053 | 0.061 | 0.346±0.013 | 0.002 |  |  |  |  |
| *CBL* | 1.096±0.009 | 0.004 | 0.943±0.030 | 0.166 | 0.277±0.015 | 0.000 |  |  |  |  |
| *FOXO* | 1.900±0.035 | 0.022 | 2.952±0.112 | 0.003 | 0.943±0.029 | 0.038 |  |  |  |  |
| *INSR* | 0.483±0.010 | 0.039 | 0.568±0.007 | 0.021 | 0.134±0.005 | 0.000 |  |  |  |  |
| *INS* | 0.554±0.001 | 0.013 | 0.526±0.098 | 0.059 | 0.846±0.145 | 0.125 |  |  |  |  |
| *AKT2* | 0.998±0.002 | 0.000 | 0.875±0.405 | 0.156 | 0.269±0.048 | 0.000 |  |  |  |  |
|  |  |  |  |  |  |  |  |  |  |  |
|  |  | | | | | | | | | |
|  | **Impaired fasting glucose (IFG) – mRNA Fold change (IFG vs Control)±SEM** | | | | | | | | | |
| **Patient**  **/mRNA** | **IFG 7** | **p-value** | **IFG 8** | **p-value** | **IFG 9** | **p-value** | **IFG 10** | **p-value** | **IFG 11** | **p-value** |
| *IRS1* | 0.744±0.127 | 0.003 | 0.901±0.031 | 0.000 | 0.762±0.157 | 0.085 | 0.637±0.005 | 0.020 | 1.058±0.021 | 0.001 |
| *PTPN1* | 0.720±0.016 | 0.000 | 0.959±0.006 | 0.000 | 0.432±0.005 | 0.000 | 1.021±0.039 | 0.015 | 0.777±0.022 | 0.000 |
| *GLUT4* | 0.874±0.003 | 0.039 | 2.684±0.050 | 0.045 | 1.138±0.020 | 0.031 | 1.330±0.031 | 0.030 | 0.806±0.050 | 0.007 |
| *CBL* | 2.016±0.028 | 0.001 | 2.490±0.028 | 0.007 | 1.091±0.044 | 0.140 | 1.134±0.021 | 0.114 | 1.583±0.018 | 0.009 |
| *FOXO* | 0.433±0.004 | 0.000 | 1.013±0.002 | 0.000 | 0.182±0.010 | 0.000 | 0.516±0.016 | 0.000 | 0.929±0.078 | 0.000 |
| *INSR* | 1.322±0.004 | 0.022 | 0.940±0.010 | 0.000 | 0.546±0.020 | 0.007 | 1.055±0.022 | 0.148 | 0.902±0.007 | 0.006 |
| *INS* | 1.585±0.022 | 0.042 | 1.143±0.007 | 0.001 | 1.371±0.024 | 0.044 | 1.041±0.010 | 0.186 | 2.595±0.012 | 0.011 |
| *AKT2* | 1.225±0.006 | 0.157 | 1.314±0.007 | 0.000 | 1.035±0.038 | 0.243 | 0.915±0.009 | 0.214 | 1.212±0.053 | 0.128 |
| **Patient**  **/mRNA** | **IFG 12** | **p-value** | **IFG 13** | **p-value** | **IFG 14** | **p-value** |  |  |  |  |
| *IRS1* | 0.875±0.021 | 0.000 | 1.111±0.003 | 0.014 | 0.366±0.066 | 0.006 |  |  |  |  |
| *PTPN1* | 0.557±0.035 | 0.000 | 0.396±0.068 | 0.000 | 1.057±0.001 | 0.000 |  |  |  |  |
| *GLUT4* | 2.124±0.003 | 0.007 | 1.278±0.015 | 0.069 | 1.301±0.019 | 0.000 |  |  |  |  |
| *CBL* | 1.277±0.008 | 0.044 | 1.009±0.013 | 0.201 | 1.047±0.011 | 0.004 |  |  |  |  |
| *FOXO* | 0.230±0.021 | 0.000 | 0.814±0.004 | 0.003 | 0.404±0.012 | 0.001 |  |  |  |  |
| *INSR* | 0.945±0.014 | 0.000 | 0.618±0.067 | 0.070 | 1.153±0.029 | 0.000 |  |  |  |  |
| *INS* | 2.109±0.024 | 0.008 | 1.289±0.041 | 0.039 | 1.013±0.020 | 0.001 |  |  |  |  |
| *AKT2* | 1.544±0.197 | 0.038 | 0.946±0.001 | 0.131 | 0.929±0.028 | 0.000 |  |  |  |  |
