# Supplementary material for: MicroRNA 144 Impairs Insulin Signaling by Inhibiting the Expression of Insulin Receptor Substrate 1 in Type 2 Diabetes Mellitus
Source: PLoS One. 2011 Aug 1;6(8):e22839. doi: 10.1371/journal.pone.0022839 (PMC3148231; doi:10.1371/journal.pone.0022839)
Supplement: Table S10 — Expression of miRNAs and mRNAs in rat models. (A) Endogenous expression levels of (abundance) of miRNAs and mRNAs in control tissues (adipose, pancreas, skeletal muscle and liver), blood and exosomes of rat model were assayed using quantitative real-time PCR analysis and expressed as delta threshold cycle (ΔCt) value with respect to 18S rRNA. Lower ΔCt value indicates higher expression levels. (B) Stem-loop RT-PCR results for T2D rat model induced by a combination of low dose STZ (40 mg/kg) and high-fat diet (Fig. 7A) (C) Quantitative Real-time PCR results for the 8 “signature” miRNA (B) respective target mRNA in T2D rat model (Fig. 7B). Expression values (B & C) are tabulated as fold change calculated as a ratio of T2D versus control, 2−ΔΔCt ± SEM. Fold change below 1 are expressed as the negative reciprocal value. Validation for each miRNA/mRNA were assayed in triplicates for 3 separate experiments. T2D, type 2 diabetes. (DOC) [file pone.0022839.s010.doc]

**S10: Expression of miRNAs and mRNAs in rat models.** **(A)** Endogenous expression levels of (abundance) of miRNAs and mRNAs in control tissues (adipose, pancreas, skeletal muscle and liver), blood and exosomes of rat model were assayed using quantitative real-time PCR analysis and expressed as delta threshold cycle (∆Ct) value with respect to 18S rRNA. Lower ∆Ct value indicates higher expression levels. **(B)** Stem-loop RT-PCR results for T2D rat model induced by a combination of low dose STZ (40mg/kg) and high-fat diet (Fig. 7A). **(C)** Quantitative Real-time PCR results for the 8 “signature” miRNA (B) respective target mRNA in T2D rat model (Fig. 7B). Expression values (B & C) are tabulated as fold change calculated as a ratio of T2D versus control, 2^-ΔΔCt^ ± SEM . Fold change below 1 are expressed as the negative reciprocal value. Validation for each miRNA/mRNA were assayed in triplicates for 3 separate experiments. T2D, type 2 diabetes.

**S10**A

|  | **∆Ct values** | | | | | |
| --- | --- | --- | --- | --- | --- | --- |
| **miRNA** | **Rat blood** | **Rat adipose** | **Rat pancreas** | **Rat skeletal muscle** | **Rat liver** | **Rat exosomes** |
| **miR-144** | 13.22 | 15.67 | 16.18 | 14.74 | 14.44 | 12.64 |
| **miR-146a** | 16.02 | 11.89 | 14.89 | 13.11 | 12.28 | 15.44 |
| **miR-150** | 12.26 | 10.41 | 15.18 | 11.25 | 14.43 | 13.12 |
| **miR-182** | 16.88 | 17.8 | 16.24 | 15.68 | 16.19 | 17.54 |
| **miR-192** | 16.87 | 15.6 | 15.84 | 16.12 | 9.96 | 15.49 |
| **miR-29a** | 13.87 | 9.19 | 10.38 | 8.94 | 9.58 | 14.45 |
| **miR-30d** | 12.57 | 11.14 | 13.29 | 9.93 | 11.72 | 15.32 |
| **miR-320** | 12.09 | 11.31 | 12.78 | 12.17 | 15.11 | 13.23 |
|  | **∆Ct values** | | | | | |
| **mRNA** | **Rat blood** | **Rat adipose** | **Rat pancreas** | **Rat skeletal muscle** | **Rat liver** | **Rat exosomes** |
| ***IRS1*** | 13.88 | 16.25 | 15.41 | 13.75 | 10.57 | 16.36 |
| ***PTPN1*** | 10.36 | 17.09 | 13.56 | 13.37 | 10.14 | 14.39 |
| ***GLUT4*** | 12.33 | 15.51 | 15.51 | 12.01 | 14.70 | 15.51 |
| ***CBL*** | 11.38 | 16.17 | 13.41 | 12.68 | 11.36 | 15.11 |
| ***FOXO*** | 13.35 | 16.39 | 16.42 | 13.65 | 11.98 | 16.42 |
| ***INS1*** | 15.94 | 16.82 | 12.60 | 17.91 | 14.32 | 17.36 |
| ***INS2*** | 11.51 | 17.21 | 11.03 | 16.71 | 14.49 | 17.73 |
| ***AKT2*** | 9.80 | 16.10 | 13.52 | 12.64 | 9.65 | 14.27 |

**S10**B

|  | **Fold change±SEM** | | | | | | | | | | | |
| --- | --- | --- | --- | --- | --- | --- | --- | --- | --- | --- | --- | --- |
|  | **Rat**  **blood** | ***p-value*** | **Rat**  **adipose** | ***p-value*** | **Rat**  **pancreas** | ***p-value*** | **Rat skeletal muscle** | ***p-value*** | **Rat**  **liver** | ***p-value*** | **Rat exosomes** | ***p-value*** |
| **miR-144** | 6.291±0.219 | *0.001* | 1.312±0.165 | *0.049* | 4.020±0.018 | *0.001* | 2.214±0.108 | *0.004* | 3.312±0.038 | *0.000* | 5.537±0.182 | *0.000* |
| **miR-146a** | -1.577±0.092 | *0.011* | -2.770±0.053 | *0.000* | -3.279±0.018 | *0.000* | -1.883±0.028 | *0.017* | -1.531±0.113 | *0.016* | -3.937±0.022 | *0.000* |
| **miR-150** | 2.140±0.164 | *0.000* | 1.560±0.253 | *0.009* | 2.941±0.020 | *0.016* | 3.400±0.018 | *0.001* | 1.747±0.061 | *0.021* | 2.892±0.173 | *0.001* |
| **miR-182** | -2.475±0.092 | *0.000* | -2.915±0.088 | *0.000* | -1.531±0.138 | *0.044* | -1.742±0.039 | *0.021* | -2.933±0.149 | *0.000* | -1.422±0.022 | *0.027* |
| **miR-192** | 3.351±0.165 | *0.000* | 1.693±0.217 | *0.016* | 3.079±0.097 | *0.000* | 2.454±0.072 | *0.003* | 1.228±0.272 | *0.051* | 2.115±0.012 | *0.018* |
| **miR-29a** | 4.350±0.262 | *0.007* | 1.500±0.184 | *0.021* | 1.009±0.118 | *0.141* | 3.030±0.120 | *0.002* | 3.390±0.087 | *0.010* | 2.353±0.194 | *0.014* |
| **miR-30d** | -1.802±0.222 | *0.041* | -1.290±0.261 | *0.051* | -1.175±0.171 | *0.100* | 1.144±0.242 | *0.182* | -1.553±0.187 | *0.029* | -9.901±0.208 | *0.000* |
| **miR-320** | 2.830±0.062 | *0.000* | 1.358±0.019 | *0.048* | 1.319±0.199 | *0.050* | 1.979±0.019 | *0.032* | 1.330±0.078 | *0.047* | 2.181±0.065 | *0.041* |

**S10**C

|  | **Fold change±SEM** | | | | | | | | | | | |
| --- | --- | --- | --- | --- | --- | --- | --- | --- | --- | --- | --- | --- |
| **mRNA** | **Rat blood** | **p-value** | **Rat adipose** | ***p-value*** | **Rat pancreas** | ***p-value*** | **Rat skeletal muscle** | ***p-value*** | **Rat liver** | ***p-value*** | **Rat exosomes** | ***p-value*** |
| ***IRS1*** | -1.499±0.118 | 0.047 | -1.206±0.220 | *0.061* | -1.479±0.118 | *0.041* | -1.466±0.117 | *0.004* | -1.511±0.137 | *0.029* | -2.370±0.019 | *0.031* |
| ***PTPN1*** | 1.307±0.082 | 0.049 | 5.408±0.158 | *0.021* | 2.550±0.247 | *0.000* | 1.530±0.050 | *0.002* | 3.099±0.096 | *0.001* | 6.501±0.127 | *0.000* |
| ***GLUT4*** | -1.862±0.143 | 0.021 | -1.149±0.062 | *0.063* | -1.167±0.077 | *0.173* | -1.435±0.077 | *0.045* | -1.366±0.228 | *0.041* | -2.105±0.093 | *0.009* |
| ***CBL*** | -1.600±0.062 | 0.050 | -1.199±0.048 | *0.107* | -1.565±0.194 | *0.050* | -3.030±0.179 | *0.003* | -1.295±0.193 | *0.051* | -1.931±0.218 | *0.013* |
| ***FOXO*** | 1.546±0.126 | 0.011 | 1.278±0.220 | *0.051* | 2.180±0.076 | *0.021* | 1.495±0.118 | *0.048* | 3.820±0.187 | *0.004* | 4.283±0.079 | *0.005* |
| ***INSR*** | -1.808±0.092 | 0.009 | -1.353±0.227 | *0.050* | -1.309±0.092 | *0.044* | -1.202±0.208 | *0.073* | -1.931±0.052 | *0.011* | -1.739±0.198 | *0.021* |
| ***INS1*** | -1.730±0.198 | 0.050 | -1.230±0.197 | *0.051* | -3.096±0.116 | *0.001* | -1.414±0.173 | *0.021* | -1.202±0.148 | *0.053* | -2.000±0.139 | *0.006* |
| ***INS2*** | -2.045±0.092 | 0.041 | -1.264±0.091 | *0.047* | -3.185±0.072 | *0.002* | -1.992±0.088 | *0.011* | -1.325±0.039 | *0.017* | -2.088±0.052 | *0.000* |
| ***AKT2*** | -2.000±0.171 | 0.011 | -2.012±0.158 | *0.011* | 1.261±0.026 | *0.050* | -1.805±0.174 | *0.006* | -1.420±0.019 | *0.010* | -2.288±0.062 | *0.017* |
