# Supplementary material for: MicroRNA 144 Impairs Insulin Signaling by Inhibiting the Expression of Insulin Receptor Substrate 1 in Type 2 Diabetes Mellitus
Source: PLoS One. 2011 Aug 1;6(8):e22839. doi: 10.1371/journal.pone.0022839 (PMC3148231; doi:10.1371/journal.pone.0022839)
Supplement: Table S11 — Quantitative Real-time PCR miRNA (A) and mRNA (B) analysis for islet primary culture ( Fig. 8A&B ). Expression values are tabulated as fold change calculated as a ratio of high glucose (25 mM) versus basal glucose control (5 mM) ratio of T2D versus control, 2−ΔΔCt ± SEM. Fold change below 1 are expressed as the negative reciprocal value. Validation for each miRNA/mRNA was assayed in triplicates for 3 separate experiments. (DOC) [file pone.0022839.s011.doc]

**S11: Quantitative Real-time PCR miRNAs and mRNAs analysis for islet primary culture (as shown in Fig. 8A&B).** Expression values are tabulated as fold change calculated as a ratio of high glucose (25mM) versus basal glucose control (5mM), 2^-ΔΔCt^ ± SEM . Fold change below 1 are expressed as the negative reciprocal value. Validation for each miRNA/mRNA was assayed in triplicates for 3 separate experiments.

| **miRNA** | **Fold change ± SEM** | ***p-value*** | **mRNA** | **Fold change ± SEM** | ***p-value*** |
| --- | --- | --- | --- | --- | --- |
| **miR-144** | 3.605±0.311 | *0.009* | ***IRS1*** | -3.257±0.291 | *0.011* |
| **miR-146a** | -1.905±0.267 | *0.047* | ***PTPN1*** | 5.175±0.281 | *0.008* |
| **miR-150** | 2.904±0.192 | *0.010* | ***GLUT4*** | -2.604±0.251 | *0.054* |
|  |  |  | ***CBL*** | -4.673±0.316 | *0.049* |
| **miR-182** | -1.267±0.291 | *0.115* | ***FOXO*** | 3.438±0.216 | *0.050* |
| **miR-192** | 1.989±0.204 | *0.045* | ***INSR*** | 1.150±0.281 | *0.105* |
| **miR-30d** | 2.969±0.184 | *0.014* | ***INS1*** | 7.757±0.193 | *0.014* |
|  |  |  | ***INS2*** | 5.500±0.208 | *0.011* |
| **miR-29a** | 1.351±0.226 | *0.140* | **A*KT2*** | -3.300±0.238 | *0.044* |
| **miR-320** | 1.692±0.251 | *0.053* |  |  |  |
