# Supplementary material for: MicroRNA 144 Impairs Insulin Signaling by Inhibiting the Expression of Insulin Receptor Substrate 1 in Type 2 Diabetes Mellitus
Source: PLoS One. 2011 Aug 1;6(8):e22839. doi: 10.1371/journal.pone.0022839 (PMC3148231; doi:10.1371/journal.pone.0022839)
Supplement: Table S12 — Binding sites of miR-144 at 3′UTR of IRS1 and mutated constructs of the binding sites. 3′UTR of IRS1 contains two miR-144 binding sites (highlighted). Fragment with 1st binding site of miR-144 alone includes 3′UTR of IRS1 from 3781-4201bp, while fragment with 2nd binding site of miR144 alone includes 3′UTR of IRS1 from 4501-4921bp. Fragment with both binding sites of miR-144 includes 3′UTR of IRS1 from 3781-4921bp. Seed region if miR-144 is shown in bold italics. Mutations are marked with asterisks. (DOC) [file pone.0022839.s012.doc]

**S12: 3’UTR of IRS1 contains two miR-144 binding sites (highlighted).** Fragment with 1^st^ binding site of miR-144 alone includes 3’UTR of IRS1 from 3781-4201bp, while fragment with 2^nd^ binding site of miR144 alone includes 3’UTR of IRS1 from 4501-4921bp. Fragment with both binding sites of miR-144 includes 3’UTR of IRS1 from 3781-4921bp. Seed region if miR-144 is shown in ***bold italics***. Mutations are marked with asterisks.

3’UTR1 of IRS1 (NM_005544.2)

| 3781 gctcaactgg acatcacagc … … … … … tcctcaggat ttcattgact ga***actgcacg*** ***ttctatattg*** ***tg***ccaagcga aaaaaaaaaa tgcactgtga caccagaata atgagtctgc ataaacttca tcttcaacct taaggactta gctggccaca gtgagctgat gtgcccacca  ccgtgtcatg agagaatggg tttactctca atgcattttc aagatacatt tcatctgctg ctgaaactgt gtacgacaaa gcatcattgt aaattatttc atacaaaact gttcacgttg ggtggagaga gtattaaata tttaacatag gttttgattt atatgtgtaa ttttttaaat gaaaatgtaa cttttcttac agcacatctt ttttttggat gtgggatgga ggtatacaat gttctgttgt aaagagtgga gcaaatgctt aaaacaaggc ttaaaagagt agaatagggt atgatccttg ttttaagatt gtaattcaga aaacataata taagaatcat agtgccatag atggttctca attgtatagt tatatttgct gatactatct cttgtcatat aaacctgatg ttgagctgag ttccttataa gaattaatct taattttgta ttttttcctg taagacaata ggccatgtta attaaactga agaaggatat atttggctgg gtgttttcaa atgtcagctt aaaattggta attgaatgga agcaaaatta taagaagagg aaattaaagt cttccattgc atgtattgta aacagaagga gatgggtgat tccttcaatt caaaagctct ctttggaatg aacaatgtgg gcgtttgtaa attctggaaa tgtctttcta ttca***taataa* *actagatact*** ***gt***tgatcttt tcttctgtcc cctcccccca ccacttctgt … … … … … ctgtctaaat gaattcatgg atgtaaatat tagtggtcct taatgtcttt gattgctgga catgaaacaa  8701 actgccaatt aaattttgcg gagacaaaaa aaa |
| --- |

| **Fragment** | **Binding of miR-144 to IRS1** |
| --- | --- |
| Site 1 | 3' UCAUGUAGU***AGAUAU***GACAU 5' hsa-miR-144 |
|  | \| \|:\|\| \|\|\|\|\|\|:\|\|\|: |
|  | 3973:5' ACUGCACGU**UCUAUA**UUGUG 3' IRS1 |
| Site 1-mut | 3' UCAUGUAGU***AGAUAU***GACAU 5' hsa-miR-144 |
|  | \| \|:\|\| ******:\|\|\|: |
|  | 3973:5' ACUGCACGU**GGGCGC**UUGUG 3' IRS1 |
| Site 2 | 3' ucAUGUAGUAGA--***UAUGACA***u 5' hsa-miR-144 |
|  | \|\| \|\| \| \|\| \|\|\|\|\|\|\| |
|  | 4805:5' caUA-AUAAACUAG**AUACUGU**u 3' IRS1 |
| Site 2- mut | 3' ucAUGUAGUAGA--***UAUGACA***u 5' hsa-miR-144 |
|  | \|\| \|\| \| \|\| ******* |
|  | 4805:5' caUA-AUAAACUAG**CGCGGCG**T 3' IRS1 |

| **Fragment** | **Binding of miR-144 to IRS1** |
| --- | --- |
| Site 1 & 2-intact | 3' UCAUGUAGU***AGAUAU***GACAU 5' hsa-miR-144 |
|  | \| \|:\|\| \|\|\|\|\|\|:\|\|\|: |
|  | 3973:5' ACUGCACGU**UCUAUA**UUGUG 3' IRS1 |
|  | 3' ucAUGUAGUAGA--***UAUGACA***u 5' hsa-miR-144 |
|  | \|\| \|\| \| \|\| \|\|\|\|\|\|\| |
|  | 4805:5' caUA-AUAAACUAG**AUACUGU**u 3' IRS1 |
| Site 1-intact & site 2-mut | 3' UCAUGUAGU***AGAUAU***GACAU 5' hsa-miR-144 |
|  | \| \|:\|\| \|\|\|\|\|\|:\|\|\|: |
|  | 3973:5' ACUGCACGU**UCUAUA**UUGUG 3' IRS1 |
|  | 3' ucAUGUAGUAGA--***UAUGACA***u 5' hsa-miR-144 |
|  | \|\| \|\| \| \|\| ******* |
|  | 4805:5' caUA-AUAAACUAG**CGCGGCG**T 3' IRS1 |
| Site 1-mut & site 2-intact | 3' UCAUGUAGU***AGAUAU***GACAU 5' hsa-miR-144 |
|  | \| \|:\|\| ******:\|\|\|: |
|  | 3973:5' ACUGCACGU**GGGCGC**UUGUG 3' IRS1 |
|  | 3' ucAUGUAGUAGA--***UAUGACA***u 5' hsa-miR-144 |
|  | \|\| \|\| \| \|\| \|\|\|\|\|\|\| |
|  | 4805:5' caUA-AUAAACUAG**AUACUGU**u 3' IRS1 |
| Both sites 1 & site 2-mut | 3' UCAUGUAGU***AGAUAU***GACAU 5' hsa-miR-144 |
|  | \| \|:\|\| ******:\|\|\|: |
|  | 3973:5' ACUGCACGU**GGGCGC**UUGUG 3' IRS1 |
|  | 3' ucAUGUAGUAGA--***UAUGACA***u 5' hsa-miR-144 |
|  | \|\| \|\| \| \|\| ******* |
|  | 4805:5' caUA-AUAAACUAG**CGCGGCG**T 3' IRS1 |
